# Design of thiazole orange oligonucleotide probes for detection of DNA and RNA by fluorescence and duplex melting†
†Electronic supplementary information (ESI) available. See DOI: 10.1039/c9ob00885c


**DOI:** 10.1039/c9ob00885c

**Published:** 2019-06-03

**Authors:** Piotr Klimkowski, Sara De Ornellas, Daniel Singleton, Afaf H. El-Sagheer, Tom Brown

**Affiliations:** a Department of Chemistry , University of Oxford , 12 Mansfield Road , Oxford , OX1 3TA , UK . Email: tom.brown@chem.ox.ac.uk; b Weatherall Institute of Molecular Medicine , John Radcliffe Hospital , Headley Way , Oxford , OX3 9DS , UK; c ATDBio , School of Chemistry University of Southampton , SO17 1BJ , UK; d Chemistry Branch , Department of Science and Mathematics , Faculty of Petroleum and Mining Engineering , Suez University , Suez 43721 , Egypt

## Abstract

We have synthesised a range of thiazole orange (TO) functionalised oligonucleotides for nucleic acid detection in which TO is attached to the nucleobase or sugar of thymidine. The properties of duplexes between TO-probes and their DNA and RNA targets strongly depend on the length of the linker between TO and the oligonucleotide, the position of attachment of TO to the nucleotide (major or minor groove) and the mode of attachment of thiazole orange (*via* benzothiazole or quinoline moiety). This information can be used to design probes for detection of target nucleic acids by fluorescence or duplex melting. With cellular imaging in mind we show that 2′-OMe RNA probes with TO at the 5-position of uracil or the 2′-position of the ribose sugar are particularly effective, exhibiting up to 44-fold fluorescence enhancement against DNA and RNA, and high duplex stability. Excellent mismatch discrimination is achieved when the mispaired base is located adjacent to the TO-modified nucleotide rather than opposite to it. The simple design, ease of synthesis and favourable properties of these TO probes suggest applications in fluorescent imaging of DNA and RNA in a cellular context.

## Abstract 2

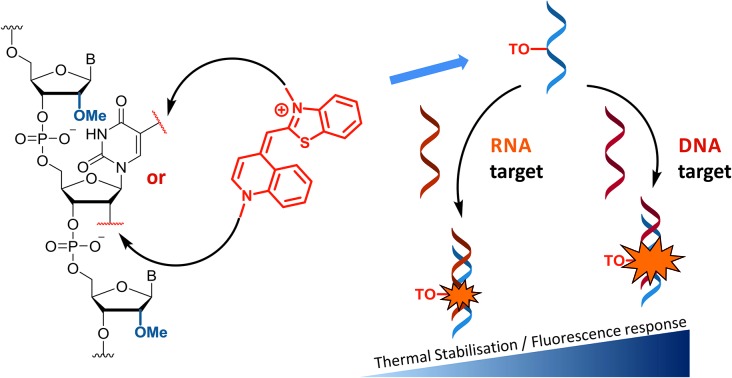
We report fluorogenic duplex-stabilising thiazole orange (TO) functionalised oligonucleotides for nucleic acid detection in which TO is attached to the nucleobase or sugar of thymidine.

## Introduction

Thiazole orange (TO) is an unsymmetrical cyanine dye which can be conjugated to oligonucleotides (ONs) to create fluorogenic hybridisation probes.^1^–^3^ TO becomes highly fluorescent upon binding to nucleic acids due to restriction of rotation about the methine bridge, hence TO–ON conjugates have been used for the detection of target nucleic acids.^4^–^10^ An important property of TO–ON probes is the significant increase in duplex stability imparted by intercalation of the TO moiety.^11^ A number of different designs of TO probe systems have been studied (Table S13†), for example PNA-based forced intercalation (FIT) probes containing TO have been shown to produce quantum yields (*Φ*) up to 0.32 on binding to complementary nucleic acids.^3^,^12^ A DNA version of FIT probes has been developed in which the quantum yield increases from 0.02 to 0.23 upon target binding,^13^,^14^ and ECHO probes, containing thymidine modified with two TO units, give low background in the single stranded form and quantum yields up to 0.44 while bound to matched RNA targets.^15^ Investigations on the relationship between the fluorogenic properties of thiazole orange and probe structure have been carried out previously. In a study in which TO was linked to the DNA phosphodiester backbone *via* its quinoline moiety, differences in fluorescence (*F*_ds_/*F*_ss_) were moderate; 2.4-fold for the TO-minor groove isomer and 0.8-fold the major groove isomer. Attachment of TO *via* its benzothiazole moiety in the major groove gave a 2.7-fold increase in fluorescence upon duplex formation and 1.3-fold increase when directed into minor groove.^7^ In a study on PNA in which TO was employed as a surrogate base the fluorescence increase upon duplex formation was large (26-fold) when TO was attached by its quinoline moiety, but was small when it was attached by its benzothiazole moiety.^16^

Recently we described a novel fluorogenic probe system (combination probes) that contains a nucleotide modified with both TO and a FRET-compatible second dye.^17^ This study, along with an awareness of the numerous potential diagnostic applications, prompted us to examine the properties of thiazole orange oligonucleotides in more detail with the aim of developing effective TO-probes that are simple to synthesise.

## Material and methods

### TO-NHS ester labelling (method 1)

To a solution of thiazole orange TO_Q/B6_ (20 eq.) in DMF (120 μL), DSC^18^,^19^ or PyBop^20^ (20 eq.) and NMM (60 eq.) were added and the mixture was shaken for 30 min at 30 °C (DSC) or 10 min at 37 °C (PyBOP) at 400 rpm. The mixture was added to an equal volume of the amino-modified oligonucleotide (200 nmol, 1.0 eq.) dissolved in carbonate buffer (NaHCO_3_/Na_2_CO_3_, 0.5 M, pH = 8.75) in an Eppendorf tube and shaken for 4 h at 37 °C. The mixture was desalted by NAP gel filtration column and purified by RP-HPLC to obtain desired pure labelled oligonucleotide, isolated yields with DSC were 40–50%, and with PyBop were 50–70%. Preparation of the amino-modified oligonucleotides is described in the ESI† and a summary of all oligonucleotide sequences is in Tables S1 and S2.†

PyBOP was found to give higher coupling efficiencies (conversion calculated by crude HPLC chromatograms at 260 nm after gel filtration, Fig. S2†) and higher isolated yields (amount of nmol obtained after HPLC purification) than DSC.

### TO-NHS ester labelling (method 2)

A solution of thiazole orange TO_Q/B6_ NHS ester (5 eq.) in DMF (80 μL) was added to an oligonucleotide (200 nmol) dissolved in carbonate buffer (NaHCO_3_/Na_2_CO_3_, 0.5 M, pH = 8.75, 120 μL) and shaken for 5 min at 20 °C. The mixture was desalted by NAP gel filtration column and purified by RP-HPLC to obtain the desired labelled oligonucleotide. Complete conversion to the product was observed by HPLC, and isolated yields were 60–75% (Fig. S3†).

## Results and discussion

### Thiazole orange probe design and synthesis

**Fig. 1:**
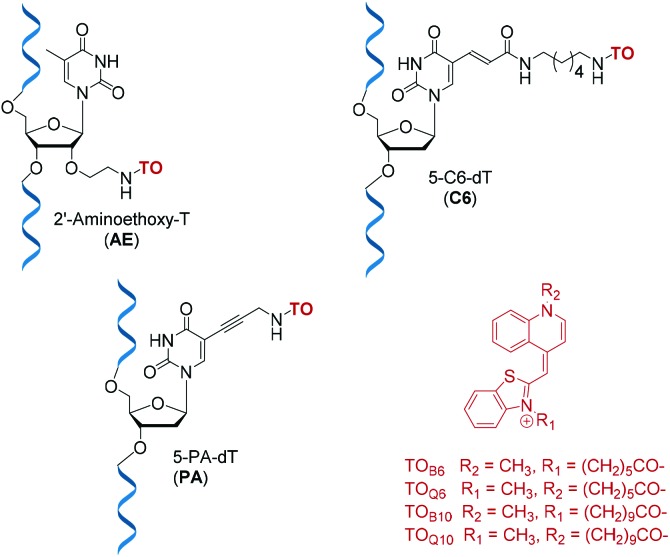
Structures of TO-modified nucleotides AE, C6, PA and the TO moieties used as carboxylic acids or NHS esters for oligonucleotide labelling.

Detection and imaging of cellular DNA and RNA are increasingly important fields of study^21^,^22^ and fluorogenic intercalators are potentially valuable in this context. Hence we undertook investigations on the fluorescent and duplex stabilising properties of a number of TO probe designs. For synthetic convenience we focused on attachment of thiazole orange to the thymidine nucleotide, but linking it to other nucleotides should also be feasible. ON probes can be modified with TO at their nucleobase, ribose sugar or the phosphodiester backbone.^23^ Another key variable is the position on thiazole orange where the linker between TO and the probe is attached; this can be *via* its benzothiazole or quinoline nitrogen.^2^,^7^ In order to investigate the effects of these alternative modes of attachment on fluorescence and duplex stability, three nucleoside monomers, 5-propargylamino-dT (PA), amino-C6-dT (C6) and 2′-aminoethoxy-T (AE) were used to introduce TO into oligonucleotides by labelling their aliphatic amino groups (Fig. 1). To achieve this, thiazole orange was functionalised with a hexanoic or decanoic acid linker on either the benzothiazole (TO_B6_/TO_B10_) or the quinoline moiety (TO_Q6_/TO_Q10_). In nucleic acid duplexes PA and C6 locate the TO dye in the vicinity of the major groove,^24^ while AE positions it in the minor groove region, thereby providing structural diversity.^25^

The phosphoramidite monomers of PA and AE, were synthesised according to published procedures,^26^–^28^ and C6 phosphoramidite (amino-C6-dT) was purchased from Glen Research. All three monomers were incorporated into a series of 10-mer oligonucleotides (ONs), which were post-synthetically labelled with TO_B6_ or TO_Q6_ in high yield using (benzotriazol-1-yloxy)tripyrrolidinophosphonium hexafluorophosphate (PyBOP) and *N*-methylmorpholine (NMM) in DMF.^29^ PyBOP was found to give superior yields to previously reported coupling methods which employed *N*,*N*′-disuccinimidyl carbonate (DSC), the reaction being complete in 4 hours (Fig. S2†).^30^,^31^ An optimised labelling procedure using the NHS esters of TO_B6_ or TO_Q6_ remarkably gave almost 100% conversion to product in just 5 minutes (Fig. S3†). These highly efficient coupling strategies were also successful in a more challenging case; the synthesis of probes with double additions of TO (Table S2†).

### DNA duplex stabilisation by TO-ODN probes

To identify the effects of base stacking on TO fluorescence and duplex stabilisation, four different ODNs sequences were synthesised with varied base sequence around the TO-modified nucleotide (Table 1A, Table S3†). Addition of TO increased duplex stability (melting temperature, *T*_m_) to quite varying degrees when the ODNs were hybridised to fully complementary DNA strands (Δ*T*_m_ = +1.3 to 14.6 °C) as previously reported in different probe systems.^4^,^32^ In the major groove (the 5-position of the thymine nucleobase), the TO_B6_ modification is more stabilising and the short linker (PA) is more effective than the long linker (C6). In the minor groove (2′-position of the sugar, AE) TO_B6_ and TO_Q6_ have similar stabilising effects. In general, a combination of PA with TO_B6_ gave the best Δ*T*_m_. There are some strong sequence-dependent effects. Most notably, in fully matched DNA duplexes, placing the TO-modified nucleotide between thymine bases (TXT) gives the least stable duplexes, whereas the other stacking environments are significantly more stabilising and are similar to each other. The TO-modified ODNs were also evaluated in mismatched duplexes by replacing the adenine base opposite to the TO-modified T with thymine to create a T : T mismatch directly opposite the TO-T nucleotide. As expected, melting temperatures were significantly lower than those with the fully complementary strands, PA-TO_B6_ showing the greatest mismatch discrimination of the series, and the TXT sequence (ODN1) was the most sensitive to the mismatch. This study indicates that thiazole orange does not prevent thermal mismatch discrimination, although it does reduce it relative to unmodified mismatched duplexes. However, locating the mispaired base directly opposite thiazole orange is not the optimum strategy for detection of point mutations or single nucleotide polymorphisms (SNPs). A superior strategy is discussed later.

**Table 1:** Changes in melting temperature (Δ*T*_m_) for DNA duplexes formed by TO-labelled ODN1–4 (A) and changes in fluorescence emission (*F*_ds_/*F*_ss_) before and after formation of matched and mismatched DNA duplexes (B). *T*_m_, *F*_ss_, *F*_ds_ were obtained in a buffer containing 10 mM phosphate 200 mM NaCl at pH 7.0. DNA concentrations for *T*_m_ studies were 3.0 μM of ODN1–4 and 3.3 μM of target strand. For *F*_ds_/*F*_ss_ 0.25 μM of ODN1–4 and 0.28 μM of target were used. *T*_m_ values are an average of 4 measurements, fluorescence data were measured at least as a duplicate. TO_Q/B6_: *λ*_ex_ = 484 nm, *λ*_em_ = 510 nm, slit ex = 7 nm, slit em = 7 nm at 20 °C

1A											
	Sequence	ODN1		ODN2		ODN3		ODN4		Average Δ*T*_m_	Average Δ*T*_m_ m–mm^*c*^
		dGCATX[combining low line]TTACG		dGCAAX[combining low line]ATACG		dGCACX[combining low line]CTACG		dGCAGX[combining low line]GTACG			
X =	Duplex	Δ*T*_m_	Δ*T*_m_ m–mm^*c*^	Δ*T*_m_	Δ*T*_m_ m–mm^*c*^	Δ*T*_m_	Δ*T*_m_ m–mm^*c*^	Δ*T*_m_	Δ*T*_m_ m–mm^*c*^		
PA-TO_Q6_	Match^*a*^	1.3	15.7	6.0	10.5	7.1	11.5	6.3	12.5	5.2	12.6
	Mismatch^*b*^	2.7		9.8		9.4		10.5		8.1	
PA-TO_B6_	Match^*a*^	6.6	14.7	12.8	12.5	14.6	13.3	12.3	14.4	11.6	13.7
	Mismatch^*b*^	9.0		14.6		15.1		14.6		13.3	

AE-TO_Q6_	Match^*a*^	7.7	11.9	10.8	12.8	9.3	8.6	9.5	12.7	9.3	11.5
	Mismatch^*b*^	12.9		12.3		14.5		13.5		13.3	
AE-TO_B6_	Match^*a*^	6.3	11.2	11.3	12.9	7.9	10.3	8.9	12.0	8.6	11.6
	Mismatch^*b*^	12.2		12.7		11.4		13.6		12.5	

C6-TO_Q6_	Match^*a*^	2.3	7.6	4.8	5.6	4.7	0.5	4.0	5.4	4.0	4.8
	Mismatch^*b*^	11.8		13.5		18.0		15.3		14.7	
C6-TO_B6_	Match^*a*^	8.4	13.3	9.9	10.4	11.5	9.7	10.3	12.0	10.0	11.4
	Mismatch^*b*^	12.2		13.8		15.6		15.0		14.2	

Average values	Match	5.4	12.4	9.3	10.8	9.2	9.0	8.6	11.5		
	Mismatch	10.1		12.8		14.0		13.8			

*T*_m_ of T*	Match	41.6	17.1	39.6	14.3	47.6	13.8	49.1	16.7		
	Mismatch	24.5		25.3		33.8		32.4			

1B											
	Sequence	ODN1		ODN2		ODN3		ODN4		Average *F*_ds_/*F*_ss_	Average *I*_m_/*I*_mm_
		dGCATX[combining low line]TTACG		dGCAAX[combining low line]ATACG		dGCACX[combining low line]CTACG		dGCAGX[combining low line]GTACG			
X =	Duplex	*F*_ds_/*F*_ss_^*d*^	*I*_m_/*I*_mm_^*e*^	*F*_ds_/*F*_ss_	*I*_m_/*I*_mm_^*e*^	*F*_ds_/*F*_ss_	*I*_m_/*I*_mm_^*e*^	*F*_ds_/*F*_ss_	*I*_m_/*I*_mm_^*e*^		
PA-TO_Q6_	Match	1.0	1.1	1.4	1.2	1.5	0.7	0.9	0.9	1.2	1.0
	Mismatch	0.9		1.3		2.6		1.3		1.5	
PA-TO_B6_	Match	3.7	3.4	5.5	1.8	5.3	1.3	2.7	2.1	4.3	2.1
	Mismatch	1.1		3.8		3.3		1.3		2.4	

AE-TO_Q6_	Match	2.8	2.0	7.4	2.7	4.1	3.5	1.3	2.1	3.9	2.6
	Mismatch	1.5		3.2		1.5		0.6		1.7	
AE-TO_B6_	Match	0.8	0.9	1.0	0.8	1.9	1.4	0.8	1.5	1.1	1.1
	Mismatch	0.9		1.2		1.3		0.6		1.0	

C6-TO_Q6_	Match	1.0	1.5	3.7	1.3	3.1	3.4	1.4	2.1	2.3	2.1
	Mismatch	0.5		2.5		0.9		0.6		1.1	
C6-TO_B6_	Match	6.6	3.3	8.6	1.9	6.9	2.8	3.0	3.8	6.3	3.0
	Mismatch	2.3		5.5		2.6		1.1		2.9	

Average values	Match	2.7	2.0	4.6	1.6	3.8	2.2	1.7	2.1		
	mismatch	1.2		2.9		2.0		0.9			

^*a*^In comparison to the matched unmodified duplex.

^*b*^In comparison to the mismatched unmodified duplex.

^*c*^Difference between *T*_m_ of matched (m) and mismatched (mm) target in °C, * = *T*_m_ value of unmodified matched and mismatched duplexes.

^*d*^Ratio of integrated fluorescence intensity for *F*_ds_ and *F*_ss_ of ODN probes with matched or mismatched targets.

^*e*^Ratio of fluorescence intensity of matched (*I*_m_) and mismatched (*I*_mm_) intensity of probes-targets duplexes at *λ*_em, max_.

### Fluorescence properties of TO-ODN probes with DNA targets

Fluorescence emission spectroscopy of the TO-modified ODNs as single strands and with their matched and mismatched complementary DNA strands showed clear trends (Table 1B, Fig. S5†).‡
‡In this study the ratio of double to single stranded fluorescence (*F*_ds_/*F*_ss_) was calculated on the basis of area under the fluorescence emission spectra from 510 nm to 650 nm. More favourable *F*_ds_/*F*_ss_ values are obtained if a narrower band is selected, *e.g.* For ODN3 C6-TO_B6_ against its complementary DNA target between 510 nm and 555 nm *F*_ds_/*F*_ss_ = 7.7 compared to 6.9 in the region 510 nm to 650 nm, and in case of 2′-OMe–(ORN3) AE-TO_Q6_ against its complementary DNA target (discussed later) the relevant values are 43.9 compared to 33.9. Both the position and mode of attachment of the dye strongly influence the fluorescence properties. C6-TO_B6_ and PA-TO_B6_ provided good discrimination between single strand (ss) and double strand (ds), and C6 is better than PA with an average 6.3-fold increase in fluorescence on duplex formation. Fluorescence-based mismatch discrimination of TO-ODNs (1–4) against DNA targets was best achieved by PA and C6 attached to TO_B6_, *i.e.* with attachment of thiazole orange in the major groove. In contrast, for AE-TO; when the dye is situated in the minor groove, the greatest changes in fluorescence occur for attachment *via* the quinoline moiety (TO_Q6_) (Fig. S5, Table S8†). In terms of the base stacking environment, the weakest fluorescence enhancement was observed with TO adjacent to guanine bases (ODN4) as previously reported.^18^,^33^ The reason for this is not because the TO moiety is less fluorescent (*i.e.* strongly quenched) in this environment, but rather that the single stranded probe is poorly quenched (Fig. S5, Tables S8 and S10†). This could be due to strong single stranded stacking between TO and guanine bases. Attempts to reduce single stranded fluorescence (*F*_ss_) of ODN4 by replacing guanine with inosine^34^ resulted in decreased *F*_ss_ but unfortunately sequence specificity and duplex stabilisation were compromised (Fig. S19 and S20†).

### Duplex stabilisation by 2′-*O*-methyl–RNA TO (2′-OMe–(ORNs)) probes with DNA and RNA targets

An important application of fluorogenic oligonucleotide probes is detection of DNA and RNA in both live and fixed cells.^35^ A common approach is to use 2′-*O*-methyl oligoribonucleotide probes (2′-OMe-ORN) instead of DNA probes, as the former are much more stable in live cells, and the latter can also cause degradation of the target mRNA *via* the action of endogenous RNase H.^13^,^36^ 2′-OMe probes have been used previously in combination with TO using a different design of the TO monomer.^37^ In order to elucidate the effects of the 2′-OMe modification on the properties of TO probes, 2′-OMe probes (2′-OMe–(ORN1–4)) with the same sequences as ODN1–4, containing AE or C6 modifications, were synthesised and labelled with TO_Q6_ and TO_B6_. These probes were tested against both DNA and RNA targets (Table 2). Increases in melting temperature were greater against DNA than RNA, and the minor groove (AE) modification was superior to the major groove (C6). For AE there were no great differences between benzothiazole and quinoline attachment (Table 2, Fig. S15 and S16†). The highest increases in *T*_m_ were observed for 2′-OMe–(ORN1) with DNA targets (AE-TO_Q6_ +18.0 °C and AE-TO_B6_ +19.3 °C). These increases are quite remarkable for a single intercalating fluorophore, and double labelled 2 × AE-TO_Q6_ provided even greater duplex stabilisation (Δ*T*_m_ = + 29.1 °C, Table S4†). Probes of this design with multiple TO insertions have great potential in duplex stabilisation and importantly are simple to synthesise. It is significant that the ability of 2′-OMe–(ORN1–4) to discriminate matched from mismatched DNA targets was preserved, and on average Δ*T*_m_ was improved slightly in comparison to ODNs probes.

**Table 2:** Changes in melting temperature (Δ*T*_m_) ODNs against RNA targets and 2′-OMe–(ORNs) against DNA and RNA targets. Conditions: see Table 1

Probe	Target (matched)	X = AE		X = C6		X = T/U
		TO_Q6_	TO_B6_	TO_Q6_	TO_B6_	—
		Δ*T*_m_^*a*^				*T*_m_
ODN1	RNA	5.7	—^*b*^	3.3	8.2	35.9
ODN2		—^*b*^	—^*b*^	1.3	5.2	32.0
ODN3		—^*b*^	—^*b*^	0.6	5.9	48.9
ODN4		—^*b*^	—^*b*^	0.8	6.3	44.6

2′-OMe–(ORN1)	RNA	7.8	7.7	4.7	7.4	42.6
2′-OMe–(5′-GCAU 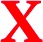 UUACG)	DNA	18.0	19.3	9.7	14.3	25.2
2′-OMe–(ORN2)	RNA	6.2	4.9	0.2	0.7	44.6
2′-OMe–(5′-GCAA 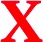 AUACG)	DNA	10.3	8.8	4.3	6.8	35.8
2′-OMe–(ORN3)	RNA	4.0	4.8	0.5	4.6	60.1
2′-OMe–(5′-GCAC 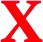 CUACG)	DNA	11.1	15.0	8.0	12.7	43.2
2′-OMe–(ORN4)	RNA	6.5	5.3	0.4	3.9	58.9
2′-OMe–(5′-GCAG 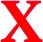 GUACG)	DNA	8.4	8.5	3.7	9.7	49.1

^*a*^In comparison to the unmodified duplex.

^*b*^No data obtained.

**Fig. 2:**
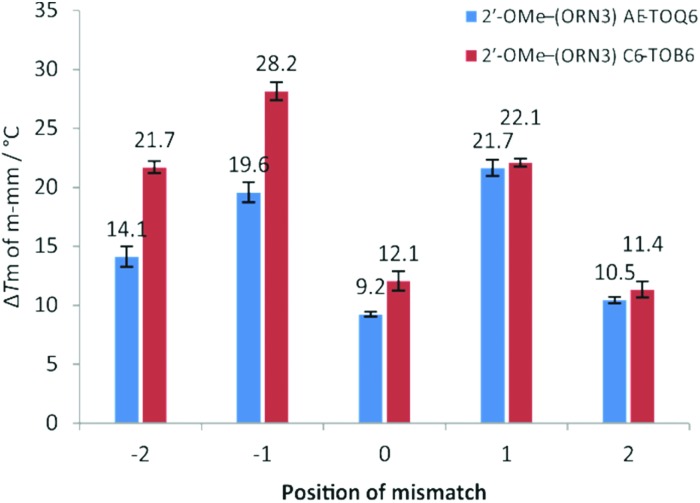
UV melting for 2′-OMe–(ORN3) (2′-OMe–(5′-GCAC
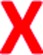
CUACG)) TO-modified oligonucleotides probes against various DNA targets. Position of mismatch refers to a mismatch in the target strand counting from the TO-modified base, *e.g.*: position “0” is directly opposite modification and “–2” is two nucleobases towards 5′ end of the probe sequence. Number above bar = Δ*T*_m_ to match target, *T*_m_ for fully matched targets 2′-OMe–(ORN3) AE-TO_Q6_: 51.7 °C; 2′-OMe–(ORN3) C6-TO_B6_: 52.5 °C, all samples measured at 1.0 μM with 1.1 eq. of target. Mismatch sequences see: Table S1,† ODN3 – targets from –2 to +2.

Next we examined the effect of mismatch position on Δ*T*_m_ (*i.e. T*_m_ of duplex containing all Watson–Crick base pairs – *T*_m_ of duplex containing a single mismatch) by constructing five different DNA hybrids that gave a single mismatch against TO-2′-OMe–(ORN3) in various positions from –2 (2 bases to the 5′-side of TO) to +2 (2 bases to the 3′-side of TO) (Fig. 2, Table S7†). A similar study was performed by Okamoto with TO probes of a different chemical composition.^38^ For both AE-TO_Q6_ and C6-TO_B6_ mismatch discrimination at positions –1 and +1 was excellent (Δ*T*_m_ > 20 °C) and far superior to the case when the mispaired base is directly opposite TO (Δ*T*_m_ = 9–12 °C). This position-dependence of mismatch discrimination is a critical consideration when designing TO probes to detect mutations or SNPs.

### Fluorescence properties of 2′-OMe–(ORNs) probes against DNA and RNA targets

**Fig. 3:**
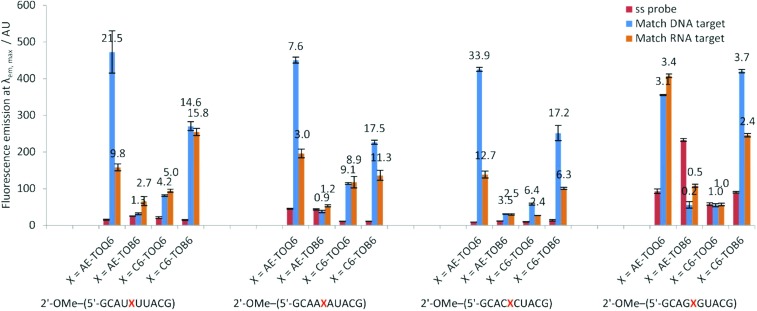
Fluorescence emission intensities at *λ*_em, max_ of a single stranded 2′-OMe–(ORN1–4) probes and DNA and RNA matched targets duplexes. Conditions: see Table 1.

**Fig. 4:**
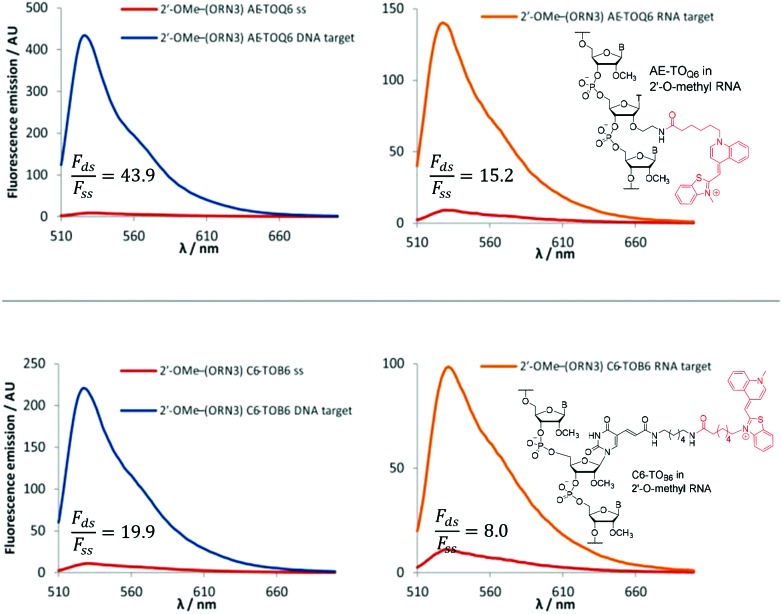
Examples of fluorescence emission spectra of single stranded (ss) probes and DNA or RNA matched target duplexes. Probes: 2′-OMe–(ORN3) (5′-GCAC
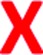
CUACG) were X = AE-TO_Q6_ or C6-TO_B6_ with structures of modification and TO isomer with optimum fluorescence. Examples of *F*_ds_/*F*_ss_ are calculated in range 510–555 nm. Conditions: see Table 1.

The 2′-OMe–(ORN1–4) series of probes show great potential as fluorogenic sensors of complementary DNA and RNA. The fluorescence enhancement upon duplex formation for AE-TO_Q6_ and C6-TO_B6_ is excellent, and in the best cases much better than the equivalent deoxyribose (DNA) versions. The probes showed the same structural preference for dye orientation as the DNA probes; the most impressive fluorescence enhancement was observed for AE-TO_Q6_ and C6-TO_B6_ against DNA targets. Limited duplex fluorescence was observed when the dye was attached in the alternative orientation (AE-TO_B6_ or C6-TO_Q6_, Fig. 3, Table 3). Surprisingly in one case (AE-TO_B6_ in 2′-OMe–ORN4) the single stranded oligonucleotide was four times as fluorescent as its duplex with complementary DNA, and twice as fluorescent as its duplex with RNA. This sequence 2′-OMe–(5′-GCAG[combining low line]X[combining low line]G[combining low line]UACG) places the thiazole orange between guanine bases and it is likely that the resulting stacking interactions are sufficiently strong to allow the dye molecule to adopt a planar fluorescent conformation. The same effect is observed with the equivalent DNA probe (AE-TO_B6_ in ODN4, Fig. S12†) but to a smaller extent, and it is unclear why in this single case the 2′-*O*-methyl sugars promote fluorescence in the single strand whereas in other cases they suppress it. Nevertheless this result shows the importance of evaluating different modes of attachment between TO and DNA/RNA. The C6-TO_B6_ modification in 2′-*O*-methyl RNA gave good *F*_ds_/*F*_ss_ ratios with DNA targets, for example 2′-OMe–(ORN3) and 2′-OMe–(ORN1) gave fluorescence enhancements of 17-fold and 15-fold respectively (Fig. 4 and S22†). Somewhat smaller increases were observed with RNA targets. With complementary DNA targets, the AE-TO_Q6_ modification attached to 2′-OMe–(ORN3) gave an impressive 34-fold enhancement (Fig. 4) (44-fold if calculated in the 510–555 nm range), and when attached to 2′-OMe–(ORN1) the enhancement was 22-fold (27-fold for the 510–555 nm range). For double TO-labelled probes, *F*_ds_/*F*_ss_ of 2 × AE-TO_Q6_ 2′-OMe–(ORN1) was reduced to 14-fold (Table S9†) which may be due to mutual quenching of TO dyes in the double strand.^14^ However, we have only so far studied a single example of dual-labelled TO probes, and it may be possible to optimise the design to improve their fluorescent properties.

**Table 3:** Changes in fluorescence emission (*F*_ds_/*F*_ss_) for 2′-OMe–(ORN1–4) with DNA targets before and after formation of matched and mismatched duplexes. Conditions: see Table 1. In this study *F*_ds_/*F*_ss_ was calculated on the basis of area under the fluorescence emission spectra from 510 nm to 650 nm. More favourable values are obtained if a narrower band in the emission spectra is selected, *e.g.* For 2′-OMe–(ORN3) AE-TO_Q6_ against its complementary DNA target between 510 nm and 555 nm; *F*_ds_/*F*_ss_ = 43.9 compared to 33.9 integrated in the region from 510 nm to 650 nm

Sequence		2′-OMe–(ORN1)		2′-OMe–(ORN2)		2′-OMe–(ORN3)		2′-OMe–(ORN4)		Average	Average
		rGCAU**X[combining low line]**UUACG		rGCAA**X[combining low line]**AUACG		rGCAC**X[combining low line]**CUACG		rGCAG**X[combining low line]**GUACG		*F*_ds_/*F*_ss_	*I*_m_/*I*_mm_
X =	Duplex	*F*_ds_/*F*_s s_^*a*^	*I*_m_/*I*_mm_^*b*^	*F*_ds_/*F*_ss_^*a*^	*I*_m_/*I*_mm_^*b*^	*F*_ds_/*F*_ss_^*a*^	*I*_m_/*I*_mm_^*b*^	*F*_ds_/*F*_ss_^*a*^	*I*_m_/*I*_mm_^*b*^		
AE-TO_Q6_	Match	21.5	2.8	7.6	2.6	33.9	2.2	3.1	2.1	16.5	2.4
	Mismatch	8.9		3.0		17.0		1.4		7.6	
AE-TO_B6_	Match	1.3	0.8	0.9	0.4	3.5	1.3	0.2	0.7	1.5	0.8
	Mismatch	1.6		1.6		2.6		0.3		1.5	

C6-TO_Q6_	Match	4.2	2.3	9.1	3.4	6.4	2.9	1.0	1.3	5.2	2.5
	Mismatch	1.7		2.8		2.0		0.7		1.8	
C6-TO_B6_	Match	14.6	6.0	17.5	1.9	17.2	3.8	3.7	3.9	13.2	3.9
	Mismatch	2.7		8.6		3.9		1.2		4.1	

Average values	Match	10.4	2.4	8.8	1.7	15.2	2.1	2.0	1.6		
	Mismatch	3.7		4.0		6.4		0.9			

^*a*^Ratio of integrated fluorescence intensity for *F*_ds_ and *F*_ss_ of 2′-OMe–(ORN) probe with matched or mismatched target.

^*b*^Ratio of fluorescence intensity of matched (*I*_m_) and mismatched (*I*_mm_) intensity at *λ*_em, max_.

Fluorescence quantum yields of AE-TO_Q6_ of 2′-OMe–(ORN3) and 2′-OMe–(ORN1) were 0.318 and 0.580 respectively against DNA targets (Table 4).^39^ This pleasingly high fluorescence enhancement is in part due to the low background fluorescence observed in the single stranded 2′-OMe RNA TO-oligonucleotides (*Φ* = 0.013–0.022, 2′-OMe–(ORN1) and 2′-OMe–(ORN3)) in comparison to equivalent ODN probes (*Φ* = 0.050–0.089, Table 4). This puts our simplified design on par with other TO probe systems.^13^,^15^ If a mispaired base is positioned opposite to TO, discrimination on the basis of fluorescence intensity (*I*_matched_/*I*_mismatched_) of 2′-*O*-methyl RNA TO probes against DNA targets is significant, *e.g.* for 2′-OMe–(ORN1) and 2′-OMe–(ORN3) with C6-TO_B6_, 6.0 and 3.8-fold differences respectively were observed, whereas for ODN1 and ODN3 probes, the corresponding differences were 3.3 and 2.8-fold. Varying the mismatch position relative to thiazole orange in the 2′-OMe–(ORN3) sequence gave significant fluorescence discrimination at positions –1 and +1 (3.4 to 3.6-fold for C_6_-TO_B6_ and 2.0 to 2.4 for AE-TO_Q6_), *i.e.* the positions at which mismatch discrimination by *T*_m_ is maximum (Fig. S21†). With further development, the favourable fluorescence properties of these simple 2′-OMe TO probes may prove useful in detection of DNA in cells.

**Table 4:** Fluorescence quantum yields of ODN1 (5′-GCAT
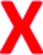
TTACG), ODN3 (5′-GCAC
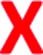
CTACG), 2′-OMe–(ORN1) (2′-OMe–(5′-GCAU
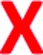
UUACG)) and 2′-OMe–(ORN3) (2′-OMe–(5′-GCAC
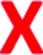
CUACG)) oligonucleotides probes with matched DNA and RNA targets. Samples were measured at *λ*_ex_ = 484 nm, *λ*_em_ = 490 nm, ex slit width = 3 nm, em slit width = 3 nm, 20 °C. Reference dye: fluorescein in 0.1 M NaOH

		*Φ*				
		ss	DNA	RNA	DNA/ss	RNA/ss
ODN1	AE-TO_Q6_	0.089	0.279	0.278	3.1	3.1
	AE-TO_B6_	0.054	0.067	0.172	1.2	3.2
	C6-TO_Q6_	0.115	0.118	0.208	1.0	1.8
	C6-TO_B6_	0.056	0.469	0.477	8.4	8.5

2′-OMe–(ORN1)	AE-TO_Q6_	0.016	0.580	0.215	36.3	13.4
	AE-TO_B6_	0.035	0.065	0.113	1.9	3.2
	C6-TO_Q6_	0.023	0.152	0.147	6.6	6.4
	C6-TO_B6_	0.019	0.493	0.477	25.9	25.1

ODN3	AE-TO_Q6_	0.051	0.061	0.043	1.2	0.8
	AE-TO_B6_	0.034	0.053	0.062	1.6	1.8
	C6-TO_Q6_	0.036	0.045	0.050	1.3	1.4
	C6-TO_B6_	0.050	0.073	0.063	1.5	1.3

2′-OMe–(ORN3)	AE-TO_Q6_	0.013	0.318	0.162	24.6	12.5
	AE-TO_B6_	0.013	0.086	0.035	6.9	2.7
	C6-TO_Q6_	0.014	0.096	0.044	7.1	3.1
	C6-TO_B6_	0.022	0.382	0.190	17.3	8.6

### Effect of linker length on the properties of 2′-OMe–(ORN1) AE-TO probes

As shown above, the properties of the TO probes are somewhat altered when they are attached to the 5-position of the nucleobase by different linkers (C6 or PA) presumably due to differences in linker length. We also explored the effects of the length of the linker between the TO and the 2′-position of the sugar (AE modification). For this study TO_B10_ and TO_Q10_ NHS esters were synthesized and used to label the 2′-OMe–(ORN1) AE and C6 probes (Table S2†). UV melting studies with fully matched DNA and RNA targets showed the same trends as previously observed for AE and C6-TO_B6/Q6_ with slightly lower increases in *T*_m_ (Fig. S18†). The fluorescence changes also followed the same trends as the C6 and AE versions against DNA and RNA targets, but with no advantage over the shorter linker (Fig. S17†). Fluorescence-based mismatch discrimination was compromised, and taking the above results into account there is no advantage to be gained by increasing linker length from 6 to 10 atoms.

Circular dichroism spectroscopy of the modified TO-containing DNA duplexes (TO-ODNs) indicated in general the formation of B-like DNA with no major perturbation from the unmodified structures (Fig. S27B, S28 and S30†). CD spectra of the 2′-OMe-(ORN) duplexes are very similar to the equivalent unmodified duplexes (Fig. S27A, S29 and S31†).

## Conclusions

In summary, we have synthesised a range of thiazole orange (TO) probes in which TO is attached to the nucleobase or sugar of a thymidine nucleotide using various linkers. The properties of duplexes between TO-probes and their DNA and RNA targets depend on the length of the linker between TO and the oligonucleotide, the position of attachment of thiazole orange to the nucleotide (nucleobase – major groove or sugar-minor groove) and the position of attachment of the linker to thiazole orange (*via* the benzothiazole or quinoline moiety). Attachment of thiazole orange *via* its benzothiazole moiety to the major groove of oligonucleotides (C6-TO_B6_) and *via* the quinoline moiety in the minor groove (AE-TO_Q6_) provides significant fluorescence enhancement on duplex formation with DNA or RNA targets. Duplex melting experiments showed higher stabilisation by C6-TO_B6_ than C6-TO_Q6_ whereas the AE modification showed similar effects for B6 and Q6 attachment. Preliminary experiments with double labelled 2′-OMe RNA (2 × AE-TO_Q6_) hybridised to complementary DNA indicate that multiple additions of TO can provide significantly enhanced duplex stability. However, the effects of multiple TO insertions on sequence specificity in a genomic context remain to be investigated. For both AE-TO_Q6_ and C6-TO_B6_ modifications, as previously described in different systems,^18^ mismatch discrimination against DNA targets at positions –1 and +1 was excellent and far superior to the case when the mispaired base is directly opposite TO. This is a crucial consideration when designing TO probes to detect mutations or SNPs. Similar effects were observed previously in other TO probe configurations.^38^ The fluorogenic properties of DNA TO-probes are limited by residual fluorescence in the single stranded state due to the cationic TO interacting with anionic DNA, causing single stranded TO-probes to adopt stable secondary structures in which the dye is fluorescent. This has been addressed previously by the use of PNA probes and ECHO probes. Here we confirm that 2′-*O*-Me RNA probes also reduce the background fluorescence^15^ whilst maintaining simplicity of design and convenience of synthesis. Anomalous fluorescence results were obtained when TO in an AE-TO_Q6_ context is placed between two guanine bases and this highlights the importance of studying the system in detail. In conclusion, the 2′-OMe-RNA TO-probes presented here have potential for applications in cell imaging of DNA and RNA and warrant further investigation, particularly in FRET systems in combination with other fluorophores.

## Conflicts of interest

There are no conflicts of interest to declare.

## Supplementary Material

Supplementary information
