# Supplementary material for: Design of thiazole orange oligonucleotide probes for detection of DNA and RNA by fluorescence and duplex melting
Source: Org Biomol Chem. 2019 Jun 3;17(24):5943–50. doi: 10.1039/c9ob00885c (PMC6686645; doi:10.1039/c9ob00885c)
Supplement: Supplementary file 1 [file OB-017-C9OB00885C-s001.pdf]

Electronic Supplementary Material (ESI) for Organic & Biomolecular Chemistry.
This journal is © The Royal Society of Chemistry 2019

Supporting Information

Design of thiazole orange oligonucleotide probes for detection of DNA and RNA by fluorescence and duplex melting

---

P. Klimkowski,[a] S. De Ornellas,[a,b] D. Singleton[c], A. H. El-Sagheer[a,d] and T. Brown[a*]

[a]*Department of Chemistry, University of Oxford, 12 Mansfield Road, Oxford, OX1 3TA, UK;* [b]*Weatherall Institute of Molecular Medicine, John Radcliffe Hospital, Headley Way, Oxford, OX3 9DS, UK;* [c]*ATDBio, School of Chemistry University of Southampton, SO17 1BJ, UK;* [d]*Chemistry Branch, Department of Science and Mathematics, Faculty of Petroleum and Mining Engineering, Suez University, Suez 43721, Egypt.*

Email: tom.brown@chem.ox.ac.uk

Table of content

General synthetic procedures 2
Synthesis of thiazole orange derivatives 3
Synthesis and purification of oligonucleotides 14
Labelling oligonucleotides with TOXY - examples 16
Steady state fluorescence measurements 22
UV melting studies 22
Quantum yield measurements 23
CD spectroscopy 24
Oligonucleotide duplex melting temperatures 25
Fluorescence data 30
Examples of fluorescence and UV melting curves 53
CD spectra 58
NMR spectra 61
References 72

General synthetic procedures

All reagents were purchased from Sigma-Aldrich, Acros Organics, Lonza, Invitrogen or Fisher Scientific and used without further purification, or dried as described below.

3 Å molecular sieves (beads, 4 – 8 mesh, Sigma-Aldrich) were used to dry MeOH and dry CH_2Cl_2 was collected from a Grubbs-type SPS. Thin layer chromatography (TLC) was performed using Merck TLC silica gel 60 F_{254} plates (0.22 mm thickness, aluminium backed) and the compounds were visualized by irradiation at 254/365 nm and stained with *p*-anisaldehyde or potassium permanganate.

^{1}H NMR spectra were measured at 400 MHz on a Bruker DPX400 (AVIIIHD 400) spectrometer. ^{13}C NMR spectra were measured at 101 MHz on a Bruker DPX400 spectrometer. ^{1}H were internally referenced to the appropriate residual undeuterated solvent signal; ^{13}C NMR spectra were referenced to the deuterated solvent. Assignment of the signals was aided by COSY (^{1}H - ^{1}H), HSQC-DEPT, HSQC (^{1}H - ^{13}C) and HMBC (^{1}H - ^{13}C) experiments.

Low-resolution mass spectra (LRMS) were recorded using electrospray ionisation (ESI^+ or ESI^-) on a Waters ZMD quadrupole mass spectrometer in HPLC grade methanol. High-resolution mass spectra (HRMS) were recorded in HPLC grade methanol using electrospray ionisation (EI) on a Bruker APEX III FT-ICR mass spectrometer.

Synthesis of thiazole orange derivatives

*Synthesis of 3-(5-carboxypentyl)-2-methylbenzothiazol-3-ium bromide (**1**)*[1]

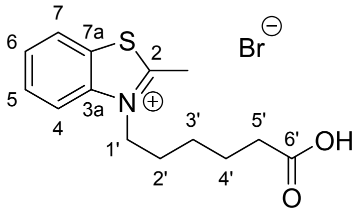

To a stirred solution of 2-methylbenzothiazole (9.3 mL, 73.2 mmol, 1.0 eq) in anhydrous MeCN (10 mL) under an argon atmosphere, 6-bromohexanoic acid (10.0 g, 51.2 mmol, 0.7 eq) was added. The solution was heated under reflux for 48 h, cooled to RT, filtered and washed with Et_2O (2 × 15 mL), to give the product as an off-white solid (7.2 g, 20.1 mmol, 39%).

^{1}H NMR (400 MHz, DMSO-d_6) δ 12.04 (br s, 1H, OH), 8.49 (dd, *J* = 8.3, 1.3 Hz, 1H, Ar), 8.36 (d, *J* = 8.5 Hz, 1H, Ar), 7.88 (ddd, *J* = 8.5, 7.2, 1.3 Hz, 1H, Ar), 7.80 (ddd, *J* = 8.3, 7.2, 1.0 Hz, 1H, Ar), 4.72 (t, *J* = 7.9 Hz, 2H, CH_2), 3.23 (s, 3H, Me), 2.22 (t, *J* = 7.2 Hz, 2H, CH_2), 1.85 (p, *J* = 7.9 Hz, 2H, CH_2), 1.56 (p, *J* = 7.2 Hz, 2H, CH_2), 1.50 – 1.40 (m, 2H, CH_2); LRMS (ESI$^+$) *m/z* (%): 264.1 $[M]^+$ (100).

Spectral data were in agreement with literature values.[1]

*Synthesis of 4-chloro-1-methylquinolin-1-ium trifluoromethanesulfonate (**2**)*

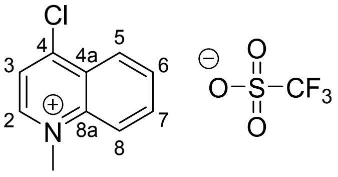

To a stirred solution of 4-chloroquinoline (0.51 g, 3.0 mmol, 1.0 eq) in anhydrous MeCN (6 mL), methyl trifluoromethanesulfonate was added (0.35 mL, 3.0 mmol, 1.0 eq). The mixture was stirred for 3 h under an argon atmosphere at RT. The reaction mixture was precipitated with Et_2O (10 mL), filtered and washed with Et_2O (2 × 5 mL) to give the product as a white solid (0.89 g, 2.7 mmol, 87%).

^{1}H NMR (400 MHz, DMSO-d_6) δ 9.51 (d, J = 6.4, 1H, H^2), 8.64 – 8.56 (m, 2H, H^5 and H^8), 8.51 (d, J = 6.4 Hz, 1H, H^3), 8.38 (ddd, J = 8.8, 7.0, 1.4 Hz, 1H, H^7), 8.18 (ddd, J = 8.1, 7.0, 0.9 Hz, 1H, H^6), 4.62 (s, 3H, N-Me); ^{13}C NMR (101 MHz, DMSO-d_6) δ 151.8, 150.1, 139.1, 136.3, 131.3, 126.9, 126.1, 122.7, 120.2, 45.4; LRMS (ESI$^+$) m/z (%): 178.0 [M]$^+$ (100); HRMS (ESI$^+$) [M]$^+$ for $C_{10}H_9N_1Cl_1^+$ calc. 178.0418 found: 178.0419.

*Synthesis of N-(5-carboxypentyl)-2-[(1,4-dihydro-1-methylquinolin-4-ylidene) methyl]benzothiazol-3-ium hexafluorophosphate (**3**, **TO$_{B6}$**)* [2]

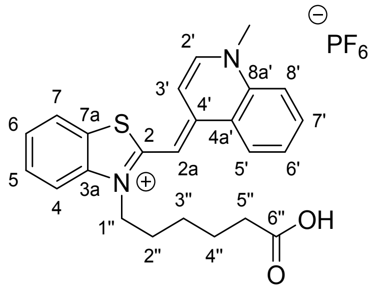

To a stirred solution of **1** (0.93 g, 2.7 mmol, 1.0 eq) and **2** (1.08 g, 3.3 mmol, 1.2 eq) in a mixture of anhydrous MeOH (34 mL) and anhydrous CH_2Cl_2 (34 mL) under an argon atmosphere at RT, was added anhydrous Et_3N (0.94 mL, 6.75 mmol, 2.5 eq). The solution turned red instantly and was then stirred for 15 min, precipitated with Et_2O (400 mL), filtered and washed with Et_2O (2 x 20 mL). The crude red solid was dissolved in THF (26 mL) and H_2O (24 mL). LiOH (207 mg, 8.6 mmol, 2.5 eq) was added and the reaction mixture was stirred for 4 h. To the stirring suspension was added HCl (37%, 1.1 mL in 15 mL H_2O) to give a red solution, the aqueous layer was extracted with CH_2Cl_2:2-propanol (v:v = 4:1; 2 × 40 mL). The organic layer was dried with $MgSO_4$, filtered and evaporated to give the product as a red solid. Salt metathesis was performed: $NaPF_6$ (0.8 g, 4.8 mmol, 2.8 eq) was added to a stirred solution of the crude product (0.97 g, 1.8 mmol, 1.0 eq) in H_2O:2-propanol (v:v = 3.4:1.0; 800 mL). A red solid precipitated and was then filtered and washed with CH_2Cl_2:2-propanol (v:v = 8:2, 2 × 10 mL) and H_2O (2 × 15 mL) to give the product as a red solid (0.59 g, 1.1 mmol, 41%).

^{1}H NMR (400 MHz, DMSO-d_6) δ 8.69 (d, *J* = 8.4 Hz, 1H, $H^{8'}$), 8.57 (d, *J* = 7.2 Hz, 1H, $H^{2'}$), 8.10 – 7.93 (m, 3H, H^4 or H^7, $H^{5'}$, $H^{6'}$), 7.78 (ddd, *J* = 8.4, 6.5, 1.5 Hz, 1H, $H^{7'}$), 7.72 (d, *J* = 8.3 Hz, 1H, H^4 or H^7), 7.62 – 7.54 (m, 1H, H^5 or H^6), 7.39 (app t, *J* = 7.6 Hz, 1H, H^5 or H^6), 7.34 (d, *J* = 7.2 Hz, 1H, $H^{3'}$), 6.88 (s, 1H, H^{2a}), 4.56 (t, *J* = 7.5 Hz, 2H, $H^{1''}$), 4.15 (s, 3H, N-Me), 2.15 (t, *J* = 7.2 Hz, 2H, $H^{5''}$), 1.77 (p, *J* = 7.5 Hz, 2H, $H^{2''}$), 1.58 (p, *J* = 7.2 Hz, 2H, $H^{4''}$), 1.51 – 1.41 (m, 2H, $H^{3''}$); ^{13}C NMR (101 MHz, DMSO-d_6) δ 174.7, 159.1, 148.6, 145.0, 139.9, 138.0, 133.2, 128.2, 127.1, 125.3, 124.4, 124.0, 123.8, 122.9, 118.2, 112.9, 107.9, 87.4, 45.6, 42.3, 34.6, 26.7, 25.8, 24.6; LRMS (ESI$^+$): *m/z* (%): 405.2 $[M]^+$ (100); LRMS (ESI$^-$): *m/z* (%): 145.0 $[PF_6]^-$ (100); HRMS (ESI$^+$) $[M]^+$ for $C_{24}H_{25}N_2O_2S^+$ calc. 405.1631 found: 405.1629.

Spectral data were in agreement with literature values.[2]

*Synthesis of N-Hydroxysuccinimide ester of N-(5-carboxypentyl)-2-[(1,4-dihydro-1-methylquinolin-4-ylidene)methyl]benzothiazol-3-ium hexafluorophosphate (**4**, **TO_{B6}-NHS**)*

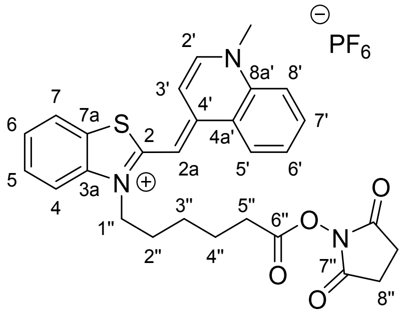

To a stirred solution of **3** (200 mg, 0.36 mmol, 1.0 eq) and DIPEA (0.32 mL, 1.84 mmol, 5.1 eq) in anhydrous DMF (3.5 mL), under an argon atmosphere at RT, was added *N*,*N*,*N*′,*N*′-tetramethyl-*O*-(*N*-succinimidyl)uronium tetrafluoroborate (TSTU) (229 mg, 0.76 mmol, 2.1 eq). The solution was stirred for 0.5 h and evaporated, CH_2Cl_2 (15 mL) was added and the solution was washed with 5% HCl (25 mL) and H_2O (25 mL). The organic layer was dried with Na_2SO_4 and evaporated, followed by purification by column chromatography on silica gel (5-60% MeCN/CH_2Cl_2) to give the product as a red solid (110 mg, 0.17 mmol, 47%).

^{1}H NMR (400 MHz, DMSO-d_6) δ 8.68 (dd, *J* = 8.5, 1.2 Hz, 1H, $H^{8'}$), 8.57 (d, *J* = 7.2 Hz, 1H, $H^{2'}$), 8.08 – 7.95 (m, 3H, $H^{5'}$, $H^{6'}$, H^4 or H^7), 7.77 (ddd, *J* = 8.5, 6.4, 1.7 Hz, 1H, $H^{7'}$), 7.72 (d, *J* = 8.4 Hz, 1H, H^4 or H^7), 7.58 (ddd, *J* = 8.4, 7.4, 1.2 Hz, 1H, H^5 or H^6), 7.40 (t, *J* = 7.4 Hz, 1H, H^5 or H^6), 7.35 (d, *J* = 7.2 Hz, 1H, $H^{3'}$), 6.88 (s, 1H, H^{2a}), 4.58 (t, *J* = 7.5 Hz, 2H, $H^{1''}$), 4.15 (s, 3H, N-Me), 2.80 (s, 4H, $H^{8''}$), 2.69 (t, *J* = 7.3 Hz, 2H, $H^{5''}$), 1.83 (p, *J* = 7.5 Hz, 2H, $H^{2''}$), 1.73 (p, *J* = 7.3 Hz,

2H, H4″), 1.57 (m, 2H, H3″); ^{13}C NMR (101 MHz, DMSO-d_6) δ 170.2, 168.9, 159.2, 148.7, 145.0, 139.9, 138.0, 133.2, 128.2, 127.0, 125.4, 124.5, 124.0, 123.8, 122.9, 118.2, 112.9, 108.0, 87.5, 45.5, 42.4, 30.0, 26.4, 25.4, 25.2, 24.0; LRMS (ESI$^+$): *m/z* (%): 502.2 [M]$^+$ (100); HRMS (ESI$^+$) [M]$^+$ for $C_{28}H_{28}N_3O_4S^+$ calc. 502.1795 found: 502.1795.

*Synthesis of 3-methyl-2-(methylthio)benzothiazol-3-ium iodide (**5**)*[3]

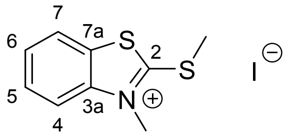

2-(Methylthio)benzothiazole (1.0 g, 5.5 mmol, 1.0 eq) was dissolved in anhydrous MeCN (3 mL) in a pressure vial under an argon atmosphere. Iodomethane (0.70 mL, 11.3 mmol, 2.1 eq) was added to the stirring solution. The mixture was stirred at 50 °C for 24 h, cooled to RT, precipitated with Et_2O (10 mL) and filtered and washed with Et_2O (2 × 5 mL) to give the product as a yellow solid (844 mg, 2.6 mmol, 47%).

^1H NMR (400 MHz, DMSO-d_6) δ 8.40 (dd, *J* = 8.2, 1.0 Hz, 1H, Ar), 8.20 (d, *J* = 8.5 Hz, 1H, Ar), 7.84 (ddd, *J* = 8.5, 7.3, 1.0 Hz, 1H, Ar), 7.73 (ddd, *J* = 8.2, 7.3, 1.0 Hz, 1H, Ar), 4.11 (s, 3H, N-Me), 3.13 (s, 3H, N-Me); LRMS (ESI$^+$) *m/z* (%): 196.0 [M]$^+$ (100).

Spectral data were in agreement with literature values.[3]

*Synthesis of 1-(5-carboxypentyl)-4-methylquinolin-1-ium bromide (**6**)*[4]

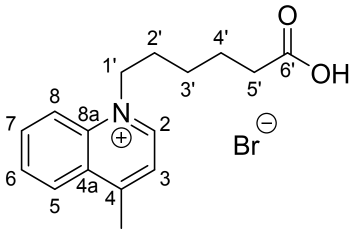

A mixture of lepidine (1.86 mL, 14.0 mmol, 1.0 eq) and 6-bromohexanoic acid (3.0 g, 15.4 mmol, 1.1 eq) was heated at 130 °C for 20 h under an argon atmosphere. The mixture was cooled to RT and stirred with acetone (30 mL) for 0.5 h. The obtained solid was filtered off and washed with Et_2O to give the product as a white solid (2.9 g, 8.6 mmol, 61%).

^{1}H NMR (400 MHz, DMSO-d_6) δ 9.47 (d, *J* = 6.0 Hz, 1H, H^2), 8.61 (d, *J* = 8.9 Hz, 1H, H^5 or H^8), 8.54 (dd, *J* = 8.5, 1.4 Hz, 1H, H^5 or H^8), 8.25 (ddd, *J* = 8.9, 6.9, 1.4 Hz, 1H, H^6 or H^7), 8.11 – 8.01 (m, 2H, H^3 and H^6 or H^7), 5.02 (t, *J* = 7.5 Hz, 2H, $H^{1'}$), 3.00 (s, 3H, Me), 2.21 (t, *J* = 7.3 Hz, 2H, $H^{5'}$), 2.01 – 1.88 (m, 2H, $H^{2'}$), 1.54 (p, *J* = 7.3 Hz, 2H, $H^{4'}$), 1.45 – 1.33 (m, 2H, $H^{3'}$); ^{13}C NMR (101 MHz, DMSO-d_6) δ 174.3, 158.6, 148.4, 136.7, 135.1, 129.6, 129.0, 127.2, 122.7, 119.4, 56.7, 33.4, 29.2, 25.3, 23.9, 19.7; LRMS (ESI$^+$): *m/z* (%): 258.2 $[M]^+$ (100).

Spectral data were in agreement with literature values.[4]

*Synthesis of N-(methyl)-2-[(1,4-dihydro-1-(5-carboxypentyl)quinolin-4-ylidene) methyl]benzothiazol-3-ium iodide (**7**, **TO$_{Q6}$**)* [5, 6]

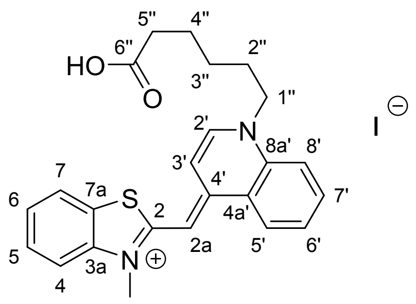

To a stirred solution of **5** (1.1g, 3.4 mmol, 1.4 eq) and **6** (0.8 g, 2.4 mmol, 1.0 eq) in anhydrous DMF (8.0 mL), under an argon atmosphere at RT, was added anhydrous Et_3N (1.0 mL, 7.2 mmol, 3.0 eq). The solution turned red instantly and stirring was continued for 0.5 h. H_2O (80 mL) was added and the mixture was acidified with 37% HCl to pH = 2. The obtained precipitate was filtered and washed with H_2O to give the product as red solid (1.0 g, 1.9 mmol, 80%).

^{1}H NMR (400 MHz, DMSO-d_6) δ 12.0 (br s, 1H, OH), 8.73 (d, *J* = 8.7 Hz, 1H, $H^{5'}$ or $H^{8'}$), 8.60 (d, *J* = 7.2 Hz, 1H, $H^{2'}$), 8.05 (d, *J* = 8.7 Hz, 1H, $H^{5'}$ or $H^{8'}$), 7.98 (dd, *J* = 8.1, 1.3 Hz, 1H, H^4), 7.94 (ddd, *J* = 8.7, 6.9, 1.2 Hz, 1H, $H^{6'}$ or $H^{7'}$), 7.74 – 7.66 (m, 2H, H^7 and $H^{6'}$ or $H^{7'}$), 7.54 (ddd, *J* = 8.4, 7.2, 1.3 Hz, 1H, H^6), 7.33 (ddd, *J* = 8.1, 7.2, 1.0 Hz, 1H, H^5), 7.24 (d, *J* = 7.2 Hz, 1H, $H^{3'}$), 6.82 (s, 1H, H^{2a}), 4.53 (t, *J* = 7.5 Hz, 2H, $H^{1''}$), 3.96 (s, 3H, N-Me), 2.22 (t, *J* = 7.3 Hz, 2H, $H^{5''}$), 1.83 (p, *J* = 7.5 Hz, 2H, $H^{2''}$), 1.55 (p, *J* = 7.3 Hz, 2H, $H^{4''}$), 1.42 – 1.32 (m, 1H, $H^{3''}$); ^{13}C NMR (101 MHz, DMSO-d_6) δ = 174.3, 159.8, 148.3, 144.2, 140.3, 136.8, 133.1, 128.0, 126.7, 125.7, 124.3, 124.1, 123.8, 122.8, 117.9, 112.8, 107.7, 88.0, 53.9, 33.9, 33.5, 28.5, 25.4, 24.0; LRMS

(ESI$^+$): *m/z* (%): 405.2 [M]$^+$ (100); HRMS (ESI$^+$) for $C_{24}H_{25}N_2O_2S^+$ [M]$^+$ calc. 405.1631 found: 405.1630.

*Synthesis of N-Hydroxysuccinimide ester of N-(methyl)-2-[(1,4-dihydro-1-(5-carboxypentyl)quinolin-4-ylidene)methyl]benzothiazol-3-ium iodide (**8, TO$_{Q6}$-NHS**)*

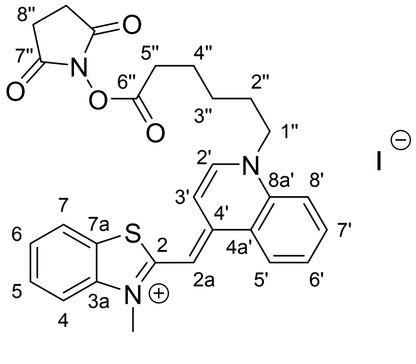

To a stirred solution of **7** (300 mg, 0.56 mmol, 1.0 eq) and DIPEA (0.49 mL, 2.78 mmol, 5.0 eq) in anhydrous DMF (5.0 mL), under an argon atmosphere at RT, was added TSTU (339 mg, 1.12 mmol, 2.0 eq). The solution was stirred for 0.5 h and 5% HCl (20 mL) was added. The mixture was then stirred for an additional 10 min. The obtained precipitate was filtered and washed with H_2O (10 mL) to give product as red solid (285 mg, 0.45 mmol, 80%).

^{1}H NMR (400 MHz, DMSO-d_6) δ 8.80 (d, *J* = 8.7 Hz, 1H, $H^{5'}$ or $H^{8'}$), 8.63 (d, *J* = 7.3 Hz, 1H, $H^{2'}$), 8.14 (d, *J* = 8.7 Hz, 1H, $H^{5'}$ or $H^{8'}$), 8.05 (dd, *J* = 7.6, 1.2 Hz, 1H, H^4), 7.99 (ddd, *J* = 8.7, 6.9, 1.3 Hz, 1H, $H^{6'}$ or $H^{7'}$), 7.85 – 7.68 (m, 2H, H^7 and $H^{6'}$ or $H^{7'}$), 7.62 (ddd, *J* = 8.4, 7.6, 1.2 Hz, 1H, H^6), 7.42 (td, *J* = 7.6, 1.0 Hz, 1H, H^5), 7.37 (d, *J* = 7.3 Hz, 1H, $H^{3'}$), 6.93 (s, 1H, H^{2a}), 4.60 (t, *J* = 7.4 Hz, 2H, $H^{1''}$), 4.02 (s, 3H, N-Me), 2.80 (s, 4H, $H^{8''}$), 2.71 (t, *J* = 7.2 Hz, 2H, $H^{5''}$), 1.90 (p, *J* = 7.6 Hz, 2H, $H^{2''}$), 1.70 (p, *J* = 7.3 Hz, 2H, $H^{4''}$), 1.58 – 1.42 (m, 2H, $H^{3''}$); ^{13}C NMR (101 MHz, DMSO-d_6) δ 170.3, 168.9, 160.0, 148.5, 144.3, 140.4, 137.0, 133.3, 128.2, 126.8, 125.8, 124.5, 124.2, 123.9, 122.9, 118.1, 113.0, 107.9, 88.1, 53.9, 33.8, 30.0, 28.3, 25.4, 25.0, 23.7; LRMS (ESI$^+$): *m/z* (%): 502.2 [M]$^+$ (100); HRMS (ESI$^+$) [M]$^+$ for $C_{28}H_{28}N_3O_4S^+$ calc. 502.1795 found: 502.1793.

*Synthesis of 3-(9-carboxynonyl)-2-methylbenzothiazol-3-ium bromide (**9**)*

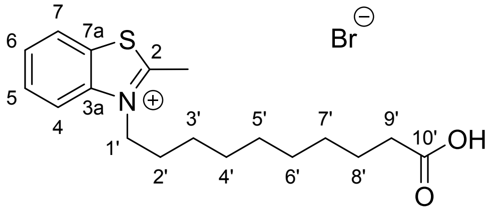

A mixture of 2-methylbenzothiazole (2.3 mL, 18.0 mmol, 1.0 eq) and 10-bromodecanoic acid (5.0 g, 20.0 mmol, 1.1 eq) was heated to 110 °C for 5 h under an argon atmosphere. The reaction mixture was dissolved in MeOH (5 mL) and precipitated with Et_2O (60 mL), filtered and washed with Et_2O (30 mL). The crude product was then purified by column chromatography on silica gel (1-15% MeOH/CH_2Cl_2) to give the product as a white solid (1.3 g, 3.2 mmol, 17%).

^{1}H NMR (400 MHz, DMSO-d_6) δ 11.94 (br s, 1H, OH), 8.49 (dd, J = 8.2, 1.3 Hz, 1H, Ar), 8.35 (d, J = 8.6 Hz, 1H, Ar), 7.88 (ddd, J = 8.6, 7.3, 1.3 Hz, 1H, Ar), 7.80 (ddd, J = 8.2, 7.3, 1.0 Hz, 1H, Ar), 4.72 (t, J = 7.8 Hz, 2H, $H^{1'}$), 3.22 (s, 3H, Me), 2.17 (t, J = 7.4 Hz, 2H, $H^{9'}$), 1.84 (p, J = 7.8 Hz, 2H, $H^{2'}$), 1.51 – 1.38 (m, 4H, CH_2), 1.35 – 1.19 (m, 8H, CH_2); ^{13}C NMR (101 MHz, DMSO-d_6) δ 177.0, 174.4, 140.8, 129.3, 129.1, 128.0, 124.7, 116.9, 49.2, 33.6, 28.7, 28.6, 28.49, 28.45, 27.8, 25.8, 24.4, 16.9; LRMS (ESI$^+$): m/z (%): 320.2 [M]$^+$ (100); HRMS (ESI$^+$) [M]$^+$ for $C_{18}H_{26}NO_2^+$ calc. 320.1679 found: 320.1677.

*Synthesis of N-(9-carboxynonyl))-2-[(1,4-dihydro-1-methylquinolin-4-ylidene)methyl] benzothiazol-3-ium trifluoromethanesulfonate (**10, TO$_{B10}$**)*

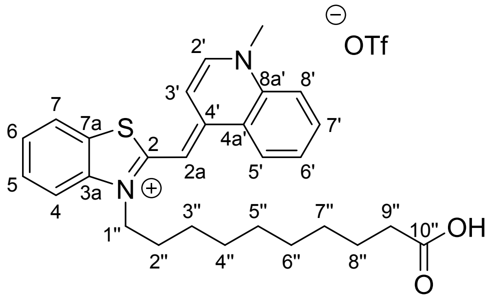

To a stirred solution of **9** (95 mg, 0.24 mmol, 1.0 eq) and **2** (80 mg, 0.24 mmol, 1.0 eq) in a mixture of anhydrous MeCN (1.0 mL) and anhydrous CH_2Cl_2 (2.0 mL) under an argon atmosphere at RT, was added anhydrous Et_3N (0.1 mL, 0.72 mmol, 3.0 eq). The solution turned red instantly and was stirred for 0.5 h. 5% HCl (10 mL) was added and the mixture

was stirred for a further 10 min. The reaction mixture was extracted with CH_2Cl_2 (2 x 10 mL), dried with Na_2SO_4 and evaporated, followed by purification by column chromatography on silica gel (1-10% MeOH/CH_2Cl_2) to give the product as a red solid (90 mg, 0.15 mmol, 63%).

^{1}H NMR (400 MHz, DMSO-d_6) δ 8.67 (t, J = 6.5 Hz, 1H, $H^{8'}$), 8.58 – 8.53 (m, 1H, $H^{2'}$), 8.04 – 7.95 (m, 3H, Ar), 7.80 – 7.73 (m, 1H, $H^{7'}$), 7.72 – 7.67 (m, 1H, Ar), 7.57 (t, J = 7.8, 1H, Ar), 7.38 (t, J = 7.8, 1H, Ar), 7.34 – 7.30 (m, 1H, $H^{3'}$), 6.87 (s, 1H, H^{2a}), 4.65 – 4.50 (m, 2H, $H^{1''}$), 4.14 (s, 3H, N-Me), 2.12 (t, J = 7.4 Hz, 2H, $H^{9''}$), 1.79 – 1.72 (m, 2H, $H^{2''}$), 1.48 – 1.36 (m, 4H, CH_2), 1.36 – 1.27 (m, 2H, CH_2), 1.25 – 1.17 (m, 6H, CH_2); ^{13}C NMR (101 MHz, DMSO-d_6) δ 174.5, 159.1, 148.5, 144.9, 139.8, 137.9, 133.1, 128.1, 126.9, 125.2, 124.4, 123.9, 123.8, 122.8, 118.2, 112.8, 107.8, 87.5, 45.6, 42.3, 33.8, 28.7, 28.58, 28.56, 28.5, 26.8, 25.9, 24.5; LRMS (ESI$^+$): m/z (%): 461.2 [M]$^+$ (100); HRMS (ESI$^+$) [M]$^+$ for $C_{28}H_{33}N_2O_2S^+$ calc. 461.2257 found: 461.2258.

*Synthesis of N-hydroxysuccinimide ester of N-(9-carboxynonyl))-2-[(1,4-dihydro-1-methylquinolin-4-ylidene)methyl]benzothiazol-3-ium trifluoromethanesulfonate (**11, TO$_{B10}$-NHS**)*

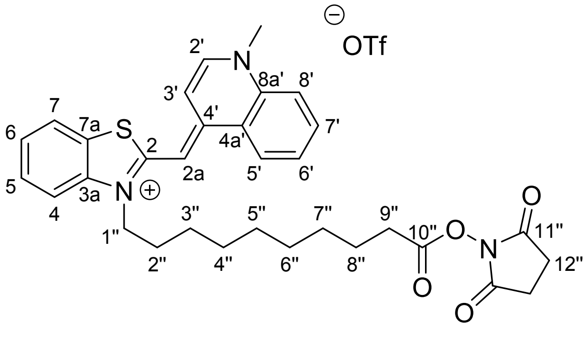

To a stirred solution of **10** (45 mg, 0.074 mmol, 1.0 eq) and DIPEA (70 µL, 0.41 mmol, 5.9 eq) in anhydrous DMF (1.0 mL), under an argon atmosphere at RT, was added TSTU (55 mg, 0.18 mmol, 2.6 eq). The solution was stirred for 0.5 h and then evaporated, CH_2Cl_2 (5 mL) was added and extracted with 5% HCl (10 mL) and H_2O (10 mL). The organic layer was dried with Na_2SO_4 and evaporated, followed by purification by column chromatography on silica gel (5-60% MeCN/CH_2Cl_2) to give the product as a red solid (40 mg, 0.064 mmol, 86%).

^{1}H NMR (400 MHz, DMSO-d_6) δ 8.71 (d, J = 8.6 Hz, 1H, $H^{8'}$), 8.59 (d, J = 7.2 Hz, 1H, $H^{2'}$), 8.10 – 7.97 (m, 3H, $H^{5'}$, $H^{6'}$, H^4 or H^7), 7.78 (ddd, J = 8.6, 6.9, 1.7 Hz, 1H, $H^{7'}$), 7.75 (d, J = 8.4 Hz, 1H, H^4 or H^7), 7.60 (ddd, J = 8.4, 7.5, 1.3 Hz, 1H, H^5 or H^6), 7.41 (t, J = 7.5 Hz, 1H, H^5 or H^6), 7.38

(d, J = 7.2 Hz, 1H, $H^{3'}$), 6.92 (s, 1H, H^{2a}), 4.61 (t, J = 7.5 Hz, 2H, $H^{1''}$), 4.17 (s, 3H, N-Me), 2.81 (s, 4H, $H^{12''}$), 2.60 (t, J = 7.2 Hz, 2H, $H^{9''}$), 1.80 (p, J = 7.5 Hz, 2H, $H^{2''}$), 1.56 (p, J = 7.2 Hz, 2H, $H^{8''}$), 1.45 (p, J = 7.5, Hz, 2H, $H^{3''}$), 1.38 – 1.18 (m, 8H, CH_2); ^{13}C NMR (101 MHz, DMSO-d_6) δ 170.2, 168.9, 159.2, 148.6, 145.0, 140.0, 138.0, 133.2, 128.2, 127.0, 125.3, 124.5, 124.0, 123.9, 122.9, 118.3, 112.9, 107.9, 87.5, 45.6, 42.4, 30.1, 28.6, 28.5, 28.3, 27.9, 26.9, 25.9, 25.4, 24.2; LRMS (ESI$^+$): *m/z* (%): 558.2 $[M]^+$ (100); HRMS (ESI$^+$) $[M]^+$ for $C_{32}H_{36}N_3O_4S^+$ calc. 558.2421 found: 558.2421.

*Synthesis of 1-(9-carboxynonyl)-4-methylquinolin-1-ium bromide (**12**)*

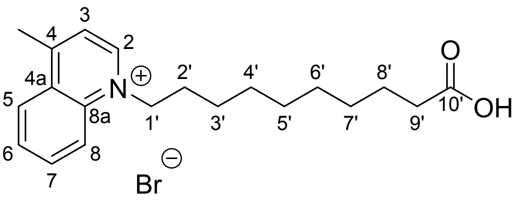

A mixture of lepidine (1.85 mL, 14.0 mmol, 1.0 eq) and 10-bromodecanoic acid (3.90 g, 15.5 mmol, 1.1 eq) was heated to 110 °C for 3 h under an argon atmosphere. The mixture was cooled to RT and stirred with acetone (30 mL) for 0.5 h. The obtained solid was filtered off and washed with Et_2O, followed by purification by column chromatography on silica gel (1-15% MeOH/CH_2Cl_2) to give the product as a white solid (1.0 g, 2.5 mmol, 18%).

^1H NMR (400 MHz, DMSO-d_6) δ 11.97 (br s, 1H, OH), 9.49 (d, J = 6.4 Hz, 1H, H^2), 8.62 (d, J = 8.9 Hz, 1H, H^8), 8.55 (dd, J = 8.4, 1.4 Hz, 1H, H^5), 8.26 (ddd, J = 8.9, 7.0, 1.4 Hz, 1H, H^7), 8.09 (d, J = 6.4 Hz, 1H, H^3), 8.06 (ddd, J = 8.4, 7.0, 0.8 Hz, 1H, H^6), 5.03 (t, J = 7.5 Hz, 2H, $H^{1'}$), 3.01 (s, 3H, Me), 2.17 (t, J = 7.4 Hz, 2H, $H^{9'}$), 1.94 (p, J = 7.5 Hz, 2H, $H^{2'}$), 1.46 (app p, J = 6.8 Hz, 2H, $H^{8'}$), 1.40 – 1.33 (m, 2H, $H^{3'}$), 1.33 – 1.20 (m, 8H, CH_2); ^{13}C NMR (101 MHz, DMSO-d_6) δ 174.5, 158.5, 148.4, 136.7, 135.1, 129.6, 129.0, 127.2, 122.7, 119.4, 56.9, 33.6, 29.4, 28.7, 28.6, 28.5, 28.4, 25.7, 24.4, 19.7; LRMS (ESI$^+$): *m/z* (%): 314.2 $[M]^+$ (100); HRMS (ESI$^+$) $[M]^+$ for $C_{20}H_{28}N_1O_2^+$ calc. 314.2115 found: 314.2110.

*Synthesis of N-(methyl)-2-[(1,4-dihydro-1-(9-carboxynonyl)quinolin-4-ylidene)methyl] benzothiazol-3-ium iodide (**13**, **TO$_{Q10}$**)*

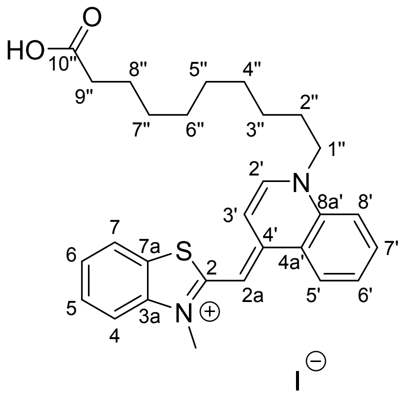

To a stirred solution of **12** (187 mg, 0.47 mmol, 1.0 eq) and **5** (153 mg, 0.47 mmol, 1.0 eq) in anhydrous DMF (3.0 mL), under an argon atmosphere at RT, was added anhydrous Et_3N (200 µL, 1.4 mmol, 3.0 eq). The solution turned red instantly and stirring was continued for 0.5 h. H_2O (16 mL) was added and the mixture was then acidified with 37% HCl to pH 2. The obtained precipitate was filtered off and washed with H_2O to give the product as a red solid (143 mg, 0.24 mmol, 51%).

^{1}H NMR (400 MHz, DMSO-d_6) δ 12.00 (br s, 1H, OH), 8.77 (d, J = 8.6 Hz, 1H, $H^{8'}$), 8.65 (d, J = 7.2 Hz, 1H, $H^{2'}$), 8.09 (dd, J = 8.7, 1.1 Hz, 1H, $H^{5'}$), 8.01 (dd, J = 7.6, 1.2 Hz, 1H, H^4), 7.96 (ddd, J = 8.7, 6.9, 1.2 Hz, 1H, $H^{6'}$), 7.77 – 7.67 (m, 2H, H^7, $H^{7'}$), 7.57 (ddd, J = 8.3, 7.6, 1.2 Hz, 1H, H^6), 7.38 (td, J = 7.6, 1.0 Hz, 1H, H^5), 7.29 (d, J = 7.2 Hz, 1H, $H^{3'}$), 6.87 (s, 1H, H^{2a}), 4.56 (t, J = 7.4 Hz, 2H, $H^{1''}$), 3.99 (s, 3H, N-Me), 2.17 (t, J = 7.2 Hz, 2H, $H^{9''}$), 1.82 (p, J = 7.4 Hz, 2H, $H^{2''}$), 1.45 (p, J = 7.2 Hz, 2H, $H^{8''}$), 1.39 – 1.25 (m, 4H, CH_2), 1.26 – 1.18 (m, 6H, CH_2); ^{13}C NMR (101 MHz, DMSO-d_6) δ 174.4, 159.9, 148.4, 144.3, 140.4, 136.9, 133.2, 128.1, 126.7, 125.8, 124.4, 124.2, 123.8, 122.8, 118.0, 112.9, 107.8, 88.1, 54.1, 33.8, 33.7, 28.8, 28.7, 28.6, 28.5, 28.4, 25.8, 24.5; LRMS (ESI$^+$): m/z (%): 461.2 [M]$^+$ (100); HRMS (ESI$^+$) [M]$^+$ for $C_{28}H_{33}N_2O_2S^+$ calc. 461.2257 found: 461.2255.

*Synthesis of N-Hydroxysuccinimide ester of N-(methyl)-2-[(1,4-dihydro-1-(9-carboxynonyl)quinolin-4-ylidene)methyl]benzothiazol-3-ium iodide (**14, TO$_{Q10}$-NHS**)*

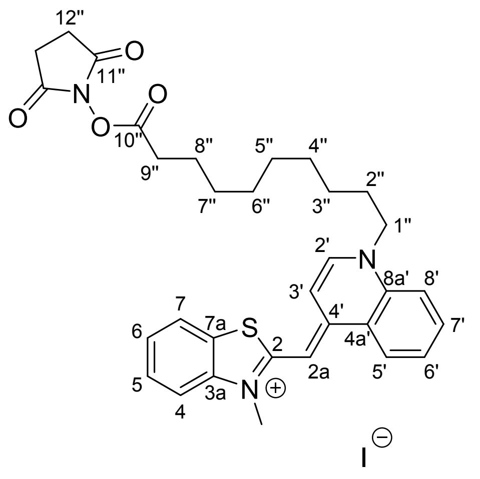

To a stirred solution of **13** (135 mg, 0.23 mmol, 1.0 eq) and DIPEA (0.18 mL, 1.2 mmol, 5.0 eq) in anhydrous DMF (2.0 mL), under an argon atmosphere at RT, was added TSTU (145 mg, 0.48 mmol, 2.1 eq). The solution was stirred for 0.5 h and 5% HCl (20 mL) was added. The mixture was then stirred for an additional 10 min. The obtained precipitate was filtered and washed with H_2O to give the product as a red solid (136 mg, 0.20 mmol, 87%).

^{1}H NMR (400 MHz, DMSO-d_6) δ 8.76 (d, J = 8.5 Hz, 1H, $H^{8'}$), 8.61 (d, J = 7.2 Hz, 1H, $H^{2'}$), 8.08 (d, J = 8.7 Hz, 1H, $H^{5'}$), 8.00 (d, J = 7.8 Hz, 1H, H^{4}), 7.96 (app t, J = 7.8 Hz, 1H, $H^{6'}$), 7.77 – 7.69 (m, 2H, H^{7}, $H^{7'}$), 7.57 (t, J = 7.8 Hz, 1H, H^{6}), 7.38 (t, J = 7.8 Hz, 1H. H^{5}), 7.28 (d, J = 7.2 Hz, 1H, $H^{3'}$), 6.86 (s, 1H, H^{2a}), 4.55 (t, J = 7.4 Hz, 2H. $H^{1''}$), 3.98 (s, 3H, N-Me), 2.81 (s, 4H, $H^{12''}$), 2.64 (t, J = 7.2 Hz, 2H, $H^{9''}$), 1.82 (p, J = 7.4 Hz, 2H, $H^{2''}$), 1.59 (p, J = 7.2 Hz, 2H, $H^{8''}$), 1.42 – 1.15 (m, 10H, CH_2); ^{13}C NMR (101 MHz, DMSO-d_6) δ 170.3, 169.0, 159.9, 148.4, 144.3, 140.4, 136.9, 133.2, 128.1, 126.8, 125.8, 124.4, 124.2, 123.8, 122.8, 118.0, 112.9, 107.8, 88.0, 54.1, 33.8, 30.2, 28.8, 28.6, 28.4, 28.3, 27.9, 25.8, 25.5, 24.3; LRMS (ESI$^+$): m/z (%): 558.2 $[M]^+$ (100); HRMS (ESI$^+$) $[M]^+$ for $C_{32}H_{36}N_3O_4S^+$ calc. 558.2421 found: 558.2421.

Synthesis and purification of oligonucleotides

Synthesis of DNA oligonucleotides

Standard DNA phosphoramidites, solid supports and additional reagents including amino-C6 dT phosphoramidite (C6, Figure S1) were purchased from Link Technologies and Applied Biosystems Ltd. Oligonucleotides were synthesized using an Applied Biosystems 394 automated DNA/RNA synthesizer using a standard 1.0 µmol phosphoramidite cycle of acid-catalyzed detritylation, coupling, capping and iodine oxidation. Stepwise coupling efficiencies and overall yields were determined by an automated trityl cation conductivity monitoring facility and in all cases were >98.0%. β-Cyanoethyl phosphoramidite monomers were dissolved in anhydrous acetonitrile to a concentration of 0.1 M immediately prior to use. The coupling time for normal A, G, C and T monomers was 35 s and the coupling time for the modified monomers (PA[7], AE[8, 9], C6, Figure S1) was 360 s. Cleavage of the oligonucleotides from the solid support and deprotection was achieved by exposure to concentrated aqueous ammonia solution (1 h, RT) followed by heating in a sealed tube (5 h, 55 °C). After evaporation of the ammonia *in vacuo*, the fully deprotected oligonucleotides were purified by reverse-phase HPLC (RP-HPLC) on a Gilson system using a Luna 10 µm C8 100 Å pore Phenomenex 10x250 mm column with a gradient of acetonitrile (HPLC grade) in water (Sterile, Millipore system) with TEAA buffer (buffer A: 0.1 M triethylammonium acetate, pH 7.0; buffer B: 0.1 M triethylammonium acetate with 50% acetonitrile, pH 7.0; gradient: 0% to 100% buffer B over 20 min, flow rate: 4 mL/min) or TEAB buffer (buffer A: 0.1 M triethylammonium bicarbonate, pH 7.5; buffer B: 0.1 M triethylammonium bicarbonate, pH 7.5, with 50% acetonitrile) were used (0% to 100% buffer B over 20 min). Elution was monitored by UV absorption at 260-299 nm. Oligonucleotides purified with TEAA buffers were desalted with GE Healthcare Life Sciences illustra NAP 25 then NAP 10 gel filtration columns. After RP-HPLC purification, all oligonucleotides were characterised by electrospray mass spectrometry using a Bruker micrOTOF II focus ESI-TOF MS or UPLC-MS Waters XEVO G2-QTOF instrument in ESI^- mode. Data were processed using MaxEnt or MassLynx v4.1.

Synthesis of RNA oligonucleotides

2′-TBDMS (Tert-Butyldimethylsilyl) protected RNA phosphoramidite monomers with *t*-butylphenoxyacetyl protection of the A, G and C nucleobases were used purchased from Sigma-Aldrich and used to assemble RNA oligonucleotides. Benzylthiotetrazole (BTT) was used as the coupling agent, *t*-butylphenoxyacetic anhydride as the capping agent and 0.1 M iodine as the oxidizing agent (Sigma-Aldrich). Coupling time of 10 min was used and coupling efficiencies of >97% were obtained. Cleavage of oligonucleotides from the solid support and protecting groups from the nucleobase and backbone were removed by exposure to concentrated aqueous ammonia : ethanol (*v*:*v* = 3:1) for 2 h at room temperature followed by heating in a sealed tube for 2 h at 55 °C.

Removal of 2′-TBDMS protection of RNA oligonucleotides

After cleavage from the solid support and removal of the protecting groups from the nucleobases and phosphodiesters in ammonia/ethanol as described above, oligonucleotides were concentrated to a small volume in vacuo, transferred to 15 mL plastic tubes and freeze dried (lyophilised). The residue was dissolved in DMSO (300 µL) and triethylamine trihydrofluoride (300 µL) was added after which the reaction mixtures were kept at 65 °C for 2.5 h. Sodium acetate (3 M, 50 µL) and butanol (3 mL) were added with vortexing and the samples were kept at -80 °C for 30 min then centrifuged at 13000 rpm at 4 °C for 10 min. The supernatant was decanted and the precipitate was washed twice with ethanol (0.75 mL) then dried under vacuum. The fully deprotected oligonucleotides were purified in similar matter as DNA equivalents.

Synthesis of 2′-OMe–RNA oligonucleotides

Phosphoramidites, solid supports and additional reagents were purchased from Link Technologies, the procedure was identical to the DNA oligonucleotides apart from extended coupling time, 10 min, for each step including modifications.

Labelling oligonucleotides with TO_{XY} - examples

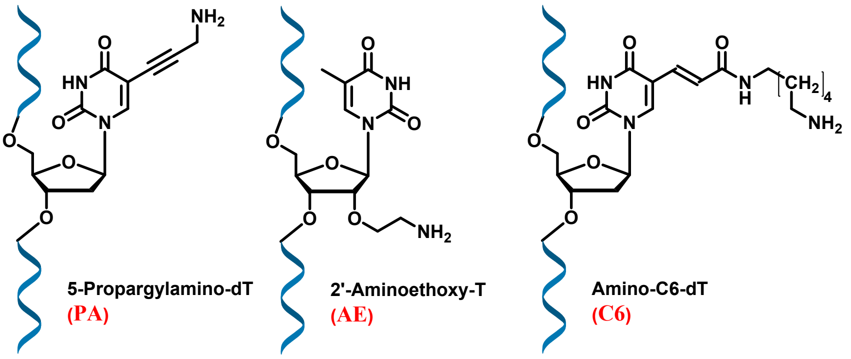

Figure S1 Artificial backbones used in this study.

A

B

C

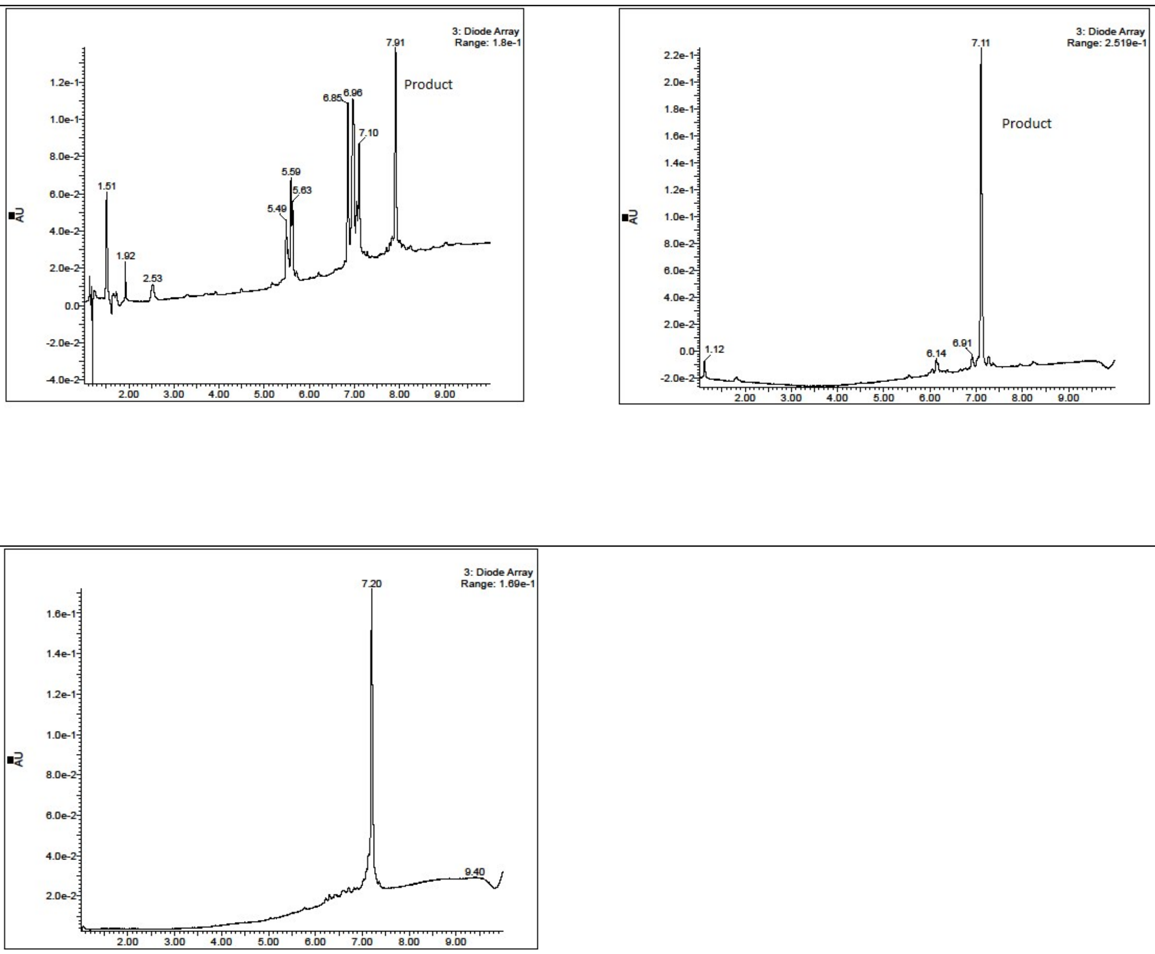

Figure S2 HPLC chromatograms at 260 nm showing labelling reactions of 5′-C**PA**GAA**PA**ATGC with TO_{B6} before HPLC purification **A**. Method 1, using DSC as the coupling reagent; **B**. Method 1, using PyBOP as the coupling reagent; **C**. HPLC purified oligonucleotide 5′-C**PA-TO$_{B6}$**GAA**PA-TO$_{B6}$**ATGC generated using DSC. Sequence was chosen as an example of more challenging double incorporation of TO and as representative case of optimised conditions for labelling. Sequence 5′-C**PA**GAA**PA**ATGC mass calculated: 3105 mass found: 3106; sequence 5′-C**PA-TO$_{B6}$**GAA**PA-TO$_{B6}$**ATGC mass calculated: 3879 mass found: 3879.

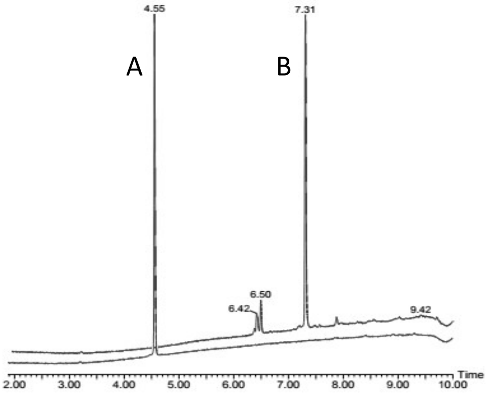

Figure S3 HPLC chromatograms at 260 nm showing efficient (5 min coupling time) labelling reaction of 2'-OMe–(ORN1) (5'-GCA**AE**UU**AE**ACG) with TO_{Q6}-NHS using Method 2 as described above. A – Unlabelled oligonucleotide: starting material, B – crude labelled product before HPLC purification.

A	B
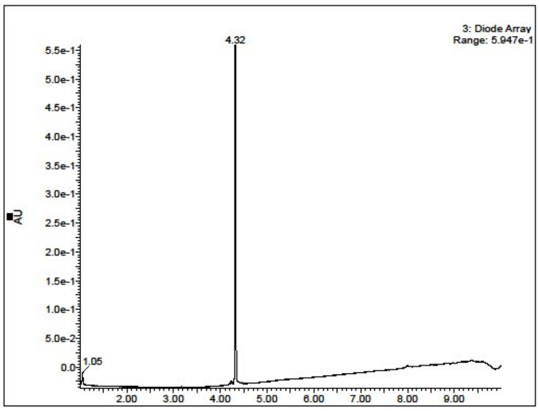	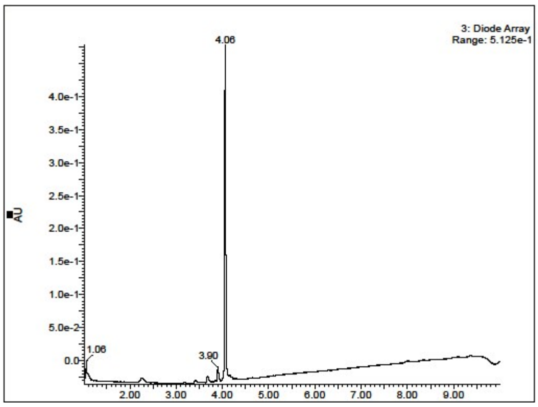

C	D
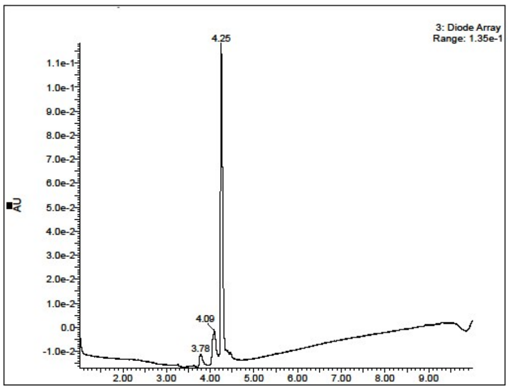	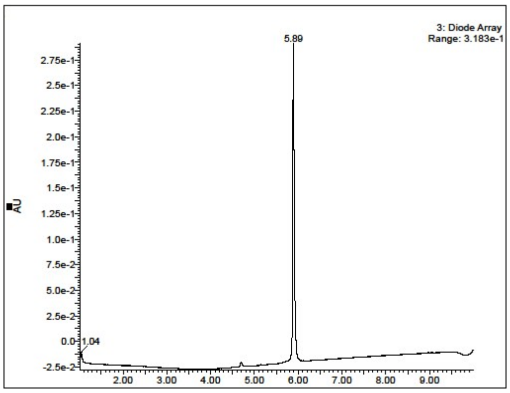

E	F
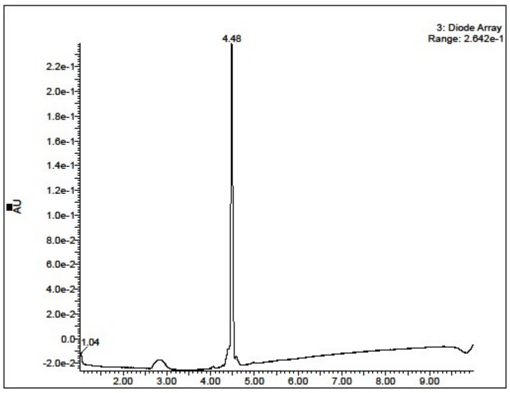	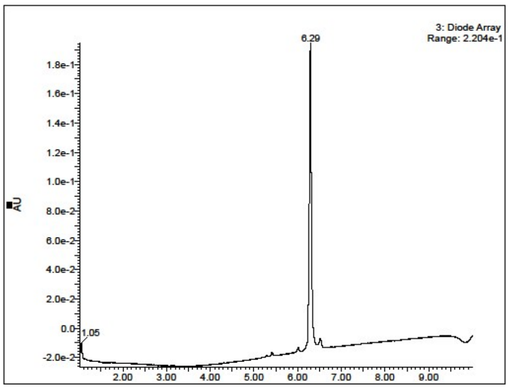

Figure S4 Examples of HPLC chromatograms at 260 nm of purified oligonucleotides: **A**. ODN1 – DNA target A (5′-CGTAAAATGC); **B**. 2′-OMe–(ORN1) – RNA target A 5′-CGUAAAAUGC; **C**. unlabelled ODN4 (5′-GCAG**PA**GTACG); **D**. labelled ODN4 (5′-GCAG**PA-TO**$_{\mathbf{Q6}}$GTACG); **E**. unlabelled 2′-OMe–(ORN1) (5′-GCAU**C6**UUACG); **F**. labelled 2′-OMe–(ORN1) (5′-GCAU**C6-TO**$_{\mathbf{B6}}$UUACG).

Table S1 Masses of oligonucleotide probes (ODNs) labelled with $TO_{B/Q6}$ and DNA targets.

ODN	X =	Sequence	Mass calculated	Mass found
ODN1 5′-GCATXTTACG	PA	5′-GCATPATTACG	3057	3057
	AE	5′-GCATAETTACG	3077	3077
	C6	5′-GCATC6TTACG	3172	3172
	PA-TO_{B6}	5′-GCATPA-TO_{B6}TTACG	3444	3444
	AE-TO_{B6}	5′-GCATAE-TO_{B6}TTACG	3464	3464
	C6-TO_{B6}	5′-GCATC6-TO_{B6}TTACG	3559	3559
	PA-TO_{Q6}	5′-GCATPA-TO_{Q6}TTACG	3444	3444
	AE-TO_{Q6}	5′-GCATAE-TO_{Q6}TTACG	3464	3464
	C6-TO_{Q6}	5′-GCATC6-TO_{Q6}TTACG	3559	3560
Unmodified ODN1	T	5′-GCATTTTACG	3018	3018
ODN1 – T target	A	5′-CGTAA**T**ATGC	3027	3027
ODN1 – A target	T	5′-CGTAA**A**ATGC	3036	3036
ODN2 5′-GCAAXATACG	PA	5′-GCAAPAATACG	3075	3075
	AE	5′-GCAAAEATACG	3095	3096
	C6	5′-GCAAC6ATACG	3190	3190
	PA-TO_{B6}	5′-GCAAPA-TO_{B6}ATACG	3462	3462
	AE-TO_{B6}	5′-GCAAAE-TO_{B6}ATACG	3482	3482
	C6-TO_{B6}	5′-GCAAC6-TO_{B6}ATACG	3577	3577
	PA-TO_{Q6}	5′-GCAAPA-TO_{Q6}ATACG	3462	3462
	AE-TO_{Q6}	5′-GCAAAE-TO_{Q6}ATACG	3482	3482
	C6-TO_{Q6}	5′-GCAAC6-TO_{Q6}ATACG	3577	3577
Unmodified ODN2	T	5′-GCAATATACG	3036	3036
ODN2 – T target	A	5′-CGTAT**T**TTGC	3009	3009
ODN2 – A target	T	5′-CGTAT**A**TTGC	3018	3018
ODN 3 5′-GCACXCTACG	PA	5′-GCACPACTACG	3027	3027
	AE	5′-GCACAECTACG	3047	3048
	AE	5′-GCAGC6GTACG	3142	3142
	PA-TO_{B6}	5′-GCACPA-TO_{B6}CTACG	3414	3413
	AE-TO_{B6}	5′-GCACAE-TO_{B6}CTACG	3434	3433
	C6-TO_{B6}	5′-GCACC6-TO_{B6}CTACG	3529	3529
	PA-TO_{Q6}	5′-GCACPA-TO_{Q6}CTACG	3414	3414
	AE-TO_{Q6}	5′-GCACAE-TO_{Q6}CTACG	3434	3434
	C6-TO_{B6}	5′-GCACC6-TO_{Q6}CTACG	3529	3529
Unmodified ODN3	T	5′-GCACTCTACG	2988	2988
ODN3 – T target (0)	A	5′-CGTAG**T**GTGC	3059	3059
ODN3 – A target	T	5′-CGTAG**A**GTGC	3068	3068
ODN3 – target (-2)		5′-CGTAGAG**A**GC	3077	3077
ODN3 – target (-1)		5′-CGTAGA**T**TGC	3043	3043
ODN3 – target (+1)		5′-CGTA**T**AGTGC	3043	3043
ODN3 – target (+2)		5′-CGT**T**GAGTGC	3059	3059

ODN4 5′-GCAGXGTACG	PA	5′-GCAGPAGTACG	3107	3108
	AE	5′-GCAGAEGTACG	3127	3128
	C6	5′-GCAGC6GTACG	3222	3221
	PA-TO_{B6}	5′-GCAGPA-TO_{B6}GTACG	3494	3494
	AE-TO_{B6}	5′-GCAGAE-TO_{B6}GTACG	3514	3514
	C6-TO_{B6}	5′-GCAGC6-TO_{B6}GTACG	3609	3609
	PA-TO_{Q6}	5′-GCAGPA-TO_{Q6}GTACG	3494	3494
	AE-TO_{Q6}	5′-GCAGAE-TO_{Q6}GTACG	3514	3514
	C6-TO_{Q6}	5′-GCAGC6-TO_{Q6}GTACG	3609	3609
ODN4′	C6	5′-GCAIC6ITACG	3192	3192
5′-GCAIXITACG	C6-TO_{B6}	5′-GCAIC6-TO_{B6}ITACG	3579	3579
I = Inosine	C6-TO_{Q6}	5′-GCAIC6-TO_{Q6}ITACG	3579	3579
Unmodified ODN4	T	5′-GCAGTGTACG	3068	3068
ODN4 – T target	A	5′-CGTAC**T**CTGC	2979	2979
ODN4 – A target	T	5′-CGTAC**A**CTGC	2988	2988

Table S2 Masses of oligonucleotide probes (2′-OMe–(ORNs)) labelled with $TO_{B/Q6}$ and RNA targets.

2′-OMe–(ORN)	X =	Sequence	Mass calculated	Mass found
2′-OMe–(ORN1) 5′-GCAUXUUACG	AE	5′-GCAUAEUUACG	3304	3305
	C6	5′-GCAUC6UUACG	3400	3400
	AE-TO_{B6}	5′-GCAUAE-TO_{B6}UUACG	3691	3692
	C6-TO_{B6}	5′-GCAUC6-TO_{B6}UUACG	3787	3787
	AE-TO_{Q6}	5′-GCAUAE-TO_{Q6}UUACG	3691	3692
	C6-TO_{Q6}	5′-GCAUC6-TO_{Q6}UUACG	3787	3786
	AE-TO_{B10}	5′-GCAUAE-TO_{B10}UUACG	3747	3748
	C6-TO_{B10}	5′-GCAUC6-TO_{B10}UUACG	3843	3843
	AE-TO_{Q10}	5′-GCAUAE-TO_{Q10}UUACG	3747	3748
	C6-TO_{Q10}	5′-GCAUC6-TO_{Q10}UUACG	3843	3843
2′-OMe– 5′-GCAXUUXACG	2 x AE	5′-GCAAEUUAEACG	3348	3348
	2 x AE-TO_{Q6}	5′-GCAAE-TO_{Q6}UUAE-TO_{Q6}ACG	4122	4122
2′-OH–(ORN1)	U	5′-GCAU**U**UUACG	3121	3122
(ORN1) – RNA target A	A	5′-CGUAA**A**AUGC	3167	3167
(ORN1) – RNA target U	U	5′-CGUAA**U**AUGC	3144	3146
2′-OMe–(ORN2) 5′-GCAAXAUACG	AE	5′-GCAAAEAUACG	3351	3352
	C6	5′-GCAAC6AUACG	3446	3446
	AE-TO_{B6}	5′-GCAAAE-TO_{B6}AUACG	3738	3738
	C6-TO_{B6}	5′-GCAAC6-TO_{B6}AUACG	3833	3833
	AE-TO_{Q6}	5′-GCAAAE-TO_{Q6}AUACG	3738	3739
	C6-TO_{Q6}	5′-GCAAC6-TO_{Q6}AUACG	3833	3833
2′-OH–(ORN2)	U	5′-GCAA**U**AUACG	3168	3167
(ORN2) – RNA target A	A	5′-CGUAU**A**UUGC	3121	3122
(ORN2) – RNA target U	U	5′-CGUAU**U**UUGC	3098	3099
2′-OMe–(ORN3) 5′-GCACXCUACG	AE	5′-GCACAECUACG	3302	3303
	C6	5′-GCACC6CUACG	3398	3399
	AE-TO_{B6}	5′-GCACAE-TO_{B6}CUACG	3689	3690
	C6-TO_{B6}	5′-GCACC6-TO_{B6}CUACG	3785	3785
	AE-TO_{Q6}	5′-GCACAE-TO_{Q6}CUACG	3689	3690
	C6-TO_{Q6}	5′-GCACC6-TO_{Q6}CUACG	3785	3786
2′-OH–(ORN3)	U	5′-GCAC**U**CUACG	3120	3119
(ORN3) – RNA target A	A	5′-CGUAG**A**GUGC	3200	3200
(ORN3) – RNA target U	U	5′-CGUAG**U**GUGC	3177	3178
2′-OMe–(ORN4) 5′-GCAGXGUACG	AE	5′-GCAGAEGUACG	3383	3384
	C6	5′-GCAGC6GUACG	3478	3478
	AE-TO_{B6}	5′-GCAGAE-TO_{B6}GUACG	3770	3771
	C6-TO_{B6}	5′-GCAGC6-TO_{B6}GUACG	3865	3864
	AE-TO_{Q6}	5′-GCAGAE-TO_{Q6}GUACG	3770	3771
	C6-TO_{Q6}	5′-GCAGC6-TO_{Q6}GUACG	3865	3864
2′-OH–(ORN4)	U	5′-GCAG**U**GUACG	3200	3200
(ORN4) – RNA target A	A	5′-CGUAC**A**CUGC	3120	3119
(ORN4) – RNA target U	U	5′-CGUAC**U**CUGC	3097	3096

Steady state fluorescence measurements

Fluorescence studies were performed on a Perkin Elmer LS50B luminescence spectrometer fitted with Perkin Elmer PTP-1 Peltier temperature controller. FLWinlabTempScan software was used with settings of 400 nm s^{-1} scan speed. The emission wavelength was recorded from 510 nm to 700 nm, excitation wavelength 484 nm, gain: high (900V), excitation slit width 7.0 nm, emission slit width 7.0 nm.

Experiment: Samples were prepared in 250 µL cuvettes with 0.25 µM probe containing TO, 200 mM NaCl, 10 mM Na-phosphate buffer pH = 7.0 at 20 °C. Spectra were recorded for the single stranded oligonucleotide probe, and then titrated with 1.1 eq of the desired target oligonucleotide and recorded again. F_{ds}/F_{ss} was calculated by comparison of the surface area under both curves (from 510 to 650 nm unless stated otherwise), each probe – target pair was recorded at least in duplicate and values are given in the charts as an averaged values (Table S8-12). Examples of fluorescence emission spectra are given in Figure S22.

UV melting studies

UV melting measurements were made on a Varian Cary 4000 UV-Vis spectrophotometer with a Cary temperature controller. Cary Win UV Thermal software was used with an absorbance wavelength of 260 nm. Samples were analysed in 1 mL quartz cuvettes (1 cm path length) and were made to 3.0 µM oligonucleotide probe concentration (X = $TO_{Q/B6}$ strand) and 3.3 µM oligonucleotide target concentration (target strand: A-match, T/U-mismatch) in phosphate buffer (NaH_2PO_4, 10 mM, NaCl 200 mM at pH 7.0, unless stated otherwise). The samples were initially denatured by heating to 85 °C at 10 °C min^{-1} then cooled to 15 °C at -1 °C min^{-1} and then maintained at 15 °C for 2 min before heating to 85 °C at 1 °C min^{-1}. UV absorption was recorded every 0.1 °C. The melting temperature (T_m) values were derived from the derivatives of the melting curves and calculated at 260 nm using Cary Win UV Thermal application software. Four successive melting curves were measured and averaged (Table S3-7). Examples of melting curves and derivatives are given in Figures S23-26.

Quantum yield measurements

Method 1

Quantum yield measurements were made using UV-Vis and fluorescence spectrometers as described above. Samples were prepared in 250 µL 10 mm pathlength cuvettes with increasing concentration of oligonucleotide in 200 mM NaCl, 10 mM Na-phosphate buffer pH = 7.0 at 20 °C. Fluorescence emission and UV absorption spectra were recorded for five concentrations (from 0.1 to 1.25 µM range). The oligonucleotide probe was treated with 1.1 eq of the desired target and recorded again for five dilution steps. Values of Φ yields were calculated by comparison of linear plot of surface area under fluorescence curve to absorption (R^2 ≥0.98) at 484 nm at given concentration, with comparison to fluorescein in 0.1 M NaOH as a standard.

Method 2

The absolute fluorescence quantum yields were measured using a SC-30 integrating sphere module (Edinburgh Instruments) and the re-absorption effect was corrected when possible. For all ODNs/2′-OMe–(ORNs) and double stranded duplexes with DNA or RNA targets, the excitation wavelength was 488 nm, the wavelength step size 0.1 nm and integration dwell time 0.25 s. All fluorescence samples were prepared with optical densities under 0.1 under ambient conditions (1 µM of ODN/2′-OMe–ORN in 1000 µL of buffer, 200 mM NaCl, 10 mM Na-phosphate pH = 7.0). Sample spectra were recorded for the single stranded ODNs/2′-OMe–(ORNs) and then titrated with 1.1 eq of the target (DNA or RNA), allowed to anneal for 2 min and recorded again at 20 °C. Three scans were repeated for both the ss and ds sample solutions and the buffer. The scattering region between 483 nm and 593 nm, and emission region between 500 and 700 nm were chosen for the calculation of the observed quantum yields.

Both method 1 and method 2 were found to give comparable values, with results within 0.4% of each other.

CD spectroscopy

CD spectra (200−360 nm) were recorded on a Jasco J-815 spectropolarimeter using a Quartz optical cell with a path length of 5.0 mm. Scans were performed at 20 °C using a step size of 0.2 nm, a time per point of 1.0 s and a bandwidth of 1 nm, with 100 nm min^{-1} scanning speed; the average of eight scans is shown (Figure S27-31). Samples from UV melting studies (3.0 µM of each oligonucleotide probe and 3.3 µM of DNA/RNA target in a 10 mM Na-phosphate buffer containing 200 mM NaCl at pH 7.0) were used directly. The average trace was smoothed (20 points) using in-built software. A CD spectrum of only buffer was also recorded and was subtracted from the collected data.

Oligonucleotide duplex melting temperatures

Table S3 Melting temperatures for TO-modified ODN1-4 duplexes with DNA targets (T_m), with comparison to unmodified duplexes (ΔT_m). Mismatch target refers to a T mismatch opposite the TO-modified base.

ODN	X =	T_m DNA match target	ΔT_m match target	T_m DNA mismatch target	ΔT_m mismatch target
	PA-TO$_{Q6}$	42.9 ± 0.3	1.3	27.2 ± 0.5	2.7
	PA-TO$_{B6}$	48.2 ± 0.2	6.6	33.5 ± 0.4	9.0
ODN1	AE-TO$_{Q6}$	49.3 ± 0.4	7.7	37.4 ± 0.5	12.9
5′-GCATXTTACG	AE-TO$_{B6}$	47.9 ± 0.4	6.3	36.7 ± 0.5	12.2
	C6-TO$_{Q6}$	43.9 ± 0.4	2.3	36.3 ± 0.5	11.8
	C6-TO$_{B6}$	50.0 ± 0.2	8.4	36.7 ± 0.3	12.2
	PA-TO$_{Q6}$	45.6 ± 0.5	6.0	35.1 ± 0.6	9.8
	PA-TO$_{B6}$	52.4 ± 0.4	12.8	39.9 ± 0.4	14.6
ODN2	AE-TO$_{Q6}$	50.4 ± 0.5	10.8	37.6 ± 0.4	12.3
5′-GCAAXATACG	AE-TO$_{B6}$	50.9 ± 0.3	11.3	38.0 ± 0.2	12.7
	C6-TO$_{Q6}$	44.4 ± 0.5	4.8	38.8 ± 0.4	13.5
	C6-TO$_{B6}$	49.5 ± 0.7	9.9	39.1 ± 0.1	13.8
	PA-TO$_{Q6}$	54.7 ± 0.4	7.1	43.2 ± 0.6	9.4
	PA-TO$_{B6}$	62.2 ± 0.6	14.6	48.9 ± 0.1	15.1
ODN3	AE-TO$_{Q6}$	56.9 ± 0.4	9.3	48.3 ± 0.4	14.5
5′-GCACXCTACG	AE-TO$_{B6}$	55.5 ± 0.7	7.9	45.2 ± 0.3	11.4
	C6-TO$_{Q6}$	52.3 ± 0.4	4.7	51.8 ± 0.4	18.0
	C6-TO$_{B6}$	59.1 ± 0.1	11.5	49.4 ± 0.4	15.6
	PA-TO$_{Q6}$	55.4 ± 0.5	6.3	42.9 ± 0.8	10.5
	PA-TO$_{B6}$	61.4 ± 0.6	12.3	47.0 ± 0.7	14.6
ODN4	AE-TO$_{Q6}$	58.6 ± 0.5	9.5	45.9 ± 0.7	13.5
5′-GCAGXGTACG	AE-TO$_{B6}$	58.0 ± 0.2	8.9	46.0 ± 0.1	13.6
	C6-TO$_{Q6}$	53.1 ± 0.4	4.0	47.7 ± 0.3	15.3
	C6-TO$_{B6}$	59.4 ± 0.2	10.3	47.4 ± 0.5	15.0
ODN4′	C6-TO$_{Q6}$	39.7 ± 0.4	-9.4	35.7 ± 0.4	3.3
5′-GCAIXITACG<br>I = Inosine	C6-TO$_{B6}$	44.8 ± 0.4	-4.3	33.8 ± 0.5	1.4
	Y = T	41.6 ± 0.7	-	24.5 ± 0.3	-
Unmodified	Y = A	39.6 ± 0.6	-	25.3 ± 0.5	-
5′-GCAYTYTACG	Y = C	47.6 ± 0.6	-	33.8 ± 0.7	-
	Y = G	49.1 ± 0.2	-	32.4 ± 0.4	-

Table S4 Melting temperatures for TO-modified 2′-OMe–(ORN1-4) duplexes with DNA targets (T_m), with comparison to unmodified duplexes (ΔT_m). Mismatch target refers to a T mismatch opposite the TO-modified base.

2′-OMe–(ORN)	X =	T_m DNA match target	ΔT_m DNA match target	T_m DNA mismatch target	ΔT_m DNA mismatch target
2′-OMe–(ORN1) 5′-GCAUXUUACG	AE-TO$_{Q6}$	43.2 ± 0.4	18.0	31.6 ± 0.3	-[b]
	AE-TO$_{B6}$	44.5 ± 0.4	19.3	32.1 ± 0.4	-[b]
	C6	24.9 ± 0.1	-0.3	-[c]	
	C6-TO$_{Q6}$	34.9 ± 0.2	9.7	27.1 ± 0.1	-[b]
	C6-TO$_{B6}$	39.5 ± 0.5	14.3	28.7 ± 0.4	-[b]
	AE-TO$_{Q10}$	43.0 ± 0.4	17.8	-[c]	
	AE-TO$_{B10}$	-[c]		-[c]	
	C6-TO$_{Q10}$	30.5 ± 0.6	5.3	-[c]	
	C6-TO$_{B10}$	35.7 ± 0.6	10.5	-[c]	
2′-OMe– 5′-GCAXUUXACG	2 × AE	30.9 ± 0.7	5.7	-[c]	-[b]
	2 × AE-TO$_{Q6}$	54.3 ± 0.4	29.1	46.0 ± 0.6	-[b]
2′-OMe–(ORN2) 5′-GCAAXAUACG	AE-TO$_{Q6}$	46.1 ± 0.9	10.3	31.9 ± 0.6	-[b]
	AE-TO$_{B6}$	44.6 ± 0.3	8.8	29.5 ± 0.2	-[b]
	C6-TO$_{Q6}$	40.1 ± 0.1	4.3	30.9 ± 0.4	-[b]
	C6-TO$_{B6}$	42.6 ± 0.3	6.8	31.2 ± 0.2	-[b]
2′-OMe–(ORN3) 5′-GCACXCUACG	AE-TO$_{Q6}$	54.3 ± 0.9	11.1	44.4 ± 0.5	15.9
	AE-TO$_{B6}$	58.2 ± 0.5	15.0	45.2 ± 0.7	16.7
	C6-TO$_{Q6}$	51.2 ± 0.2	8.0	42.1 ± 0.7	13.6
	C6-TO$_{B6}$	55.9 ± 0.5	12.7	45.1 ± 0.2	16.6
2′-OMe–(ORN4) 5′-GCAGXGUACG	AE-TO$_{Q6}$	57.5 ± 0.5	8.4	44.8 ± 0.4	12
	AE-TO$_{B6}$	57.6 ± 0.6	8.5	43.3 ± 0.5	10.5
	C6-TO$_{Q6}$	52.8 ± 0.6	3.7	41.2 ± 0.2	8.4
	C6-TO$_{B6}$	58.8 ± 0.4	9.7	43.4 ± 0.1	10.6
Unmodified 2′-OH–RNA 5′-GCAYUYUACG	Y = U	25.2 ± 0.5	-	-[a]	-
	Y = A	35.8 ± 0.5	-	-[a]	-
	Y = C	43.2 ± 0.6	-	28.5 ± 0.5	-
	Y = G	49.1 ± 0.3	-	32.8 ± 0.3	-

[a]-no melting observed, [b]-no reference melting curve, [c]-No data obtained/measurment wasn't performed

Table S5 Melting temperatures for TO-modified ODN1-4 duplexes with RNA targets (T_m), with comparison to unmodified duplexes (ΔT_m). Mismatch target refers to a U mismatch opposite the TO-modified base.

ODN	X =	T_m RNA match target	ΔT_m RNA match target	T_m RNA mismatch target	ΔT_m RNA mismatch target
	AE-TO$_{Q6}$	41.6 ± 0.2	5.6	27.8 ± 0.4	-[b]
ODN1	AE-TO$_{B6}$	-[c]		-[c]	-
5′-GCATXTTACG	C6-TO$_{Q6}$	39.2 ± 0.3	3.3	25.0 ± 0.4	-[b]
	C6-TO$_{B6}$	44.1 ± 0.1	8.1	27.7 ± 0.5	-[b]
	AE-TO$_{Q6}$	-[c]		-[c]	
ODN2	AE-TO$_{B6}$	-[c]		-[c]	
5′-GCAAXATACG	C6-TO$_{Q6}$	33.3 ± 0.4	1.3	22.9 ± 0.5	-[b]
	C6-TO$_{B6}$	37.2 ± 0.7	5.2	23.2 ± 0.5	-[b]
	AE-TO$_{Q6}$	-[c]		-[c]	
ODN3	AE-TO$_{B6}$	-[c]		38.0 ± 0.9	4.8
5′-GCACXCTACG	C6-TO$_{Q6}$	49.5 ± 0.5	0.6	34.4 ± 0.4	1.2
	C6-TO$_{B6}$	54.8 ± 0.7	5.9	38.6 ± 0.4	5.4
	AE-TO$_{Q6}$	-[c]		-[c]	
ODN4	AE-TO$_{B6}$	-[c]		-[c]	
5′-GCAGXGTACG	C6-TO$_{Q6}$	45.4 ± 0.6	0.8	32.6 ± 0.7	5.6
	C6-TO$_{B6}$	50.9 ± 0.4	6.3	34.8 ± 0.9	7.8
ODN4′	C6-TO$_{Q6}$	33.2 ± 0.5	-11.4	24.2 ± 0.9	-2.8
5′-GCAIXITACG<br>I = Inosine	C6-TO$_{B6}$	37.2 ± 0.6	-7.4	24.1 ± 0.1	-2.9
	Y = T	35.9 ± 0.3	-	-[a]	-
Unmodified	Y = A	32.0 ± 0.2	-	-[a]	-
5′-GCAYTYTACG	Y = C	48.9 ± 0.3	-	33.2 ± 0.3	-
	Y = G	44.6 ± 0.5	-	27.0 ± 0.7	-

[a]-no melting observed, [b]-no reference melting curve, [c]-no data obtained/measurement wasn't performed

Table S6 Melting temperatures for TO-modified 2′-OMe–(ORN1-4) duplexes with RNA targets (T_m), with comparison to unmodified duplexes (ΔT_m). Mismatch target refers to a U mismatch opposite the TO-modified base.

2′-OMe–(ORN)	X =	T_m RNA match target	ΔT_m RNA match target	T_m RNA mismatch target	ΔT_m RNA mismatch target
2′-OMe–(ORN1) 5′-GCAUXUUACG	AE-TO$_{Q6}$	50.4 ± 0.3	7.8	39.9 ± 0.4	9.5
	AE-TO$_{B6}$	50.3 ± 0.4	7.7	40.4 ± 0.4	10
	C6	47.4 ± 0.6	4.8	-[a]	
	C6-TO$_{Q6}$	47.3 ± 0.5	4.7	36.7 ± 0.5	6.3
	C6-TO$_{B6}$	50.0 ± 0.2	7.4	39.2 ± 0.3	8.8
	AE-TO$_{Q10}$	44.8 ± 1.1	2.2	-[a]	
	AE-TO$_{B10}$	-[a]		-[a]	
	C6-TO$_{Q10}$	43.2 ± 0.6	0.6	-[a]	
	C6-TO$_{B10}$	47.1 ± 0.03	4.5	-[a]	
2′-OMe– 5′-GCAXUUXACG	2 × AE	48.7 ± 0.7	6.1	-[a]	
	2 × AE-TO$_{Q6}$	57.2 ± 0.4	14.6	48.5 ± 0.3	18.1
2′-OMe–(ORN2) 5′-GCAAXAUACG	AE-TO$_{Q6}$	50.8 ± 0.5	6.2	37.4 ± 0.6	6.4
	AE-TO$_{B6}$	49.5 ± 0.6	4.9	36.5 ± 0.5	5.5
	C6-TO$_{Q6}$	44.8 ± 0.4	0.2	32.2 ± 0.7	1.2
	C6-TO$_{B6}$	45.3 ± 0.5	0.7	32.7 ± 0.6	1.7
2′-OMe–(ORN3) 5′-GCACXCUACG	AE-TO$_{Q6}$	64.1 ± 0.6	4.0	53.4 ± 0.7	7.3
	AE-TO$_{B6}$	64.9 ± 0.4	4.8	52.7 ± 0.7	6.6
	C6-TO$_{Q6}$	60.6 ± 0.5	0.5	49.0 ± 0.8	2.9
	C6-TO$_{B6}$	64.7 ± 0.6	4.6	53.0 ± 0.6	6.9
2′-OMe–(ORN4) 5′-GCAGXGUACG	AE-TO$_{Q6}$	65.4 ± 0.8	6.5	52.6 ± 0.6	6.8
	AE-TO$_{B6}$	64.2 ± 0.7	5.3	52.2 ± 0.7	6.4
	C6-TO$_{Q6}$	59.3 ± 0.6	0.4	46.9 ± 0.1	1.1
	C6-TO$_{B6}$	62.8 ± 0.5	3.9	48.9 ± 0.4	3.1
Unmodified 2′-OH–RNA 5′-GCAYUYUACG	Y = U	42.6 ± 0.5	-	30.4 ± 0.6	-
	Y = A	44.6 ± 0.6	-	31.0 ± 0.1	-
	Y = C	60.1 ± 0.2	-	46.1 ± 0.1	-
	Y = G	58.9 ± 0.3	-	45.8 ± 0.3	-

[a] -no data obtained/measurement wasn't performed

Table S7 Effect of the mismatch position on melting temperatures of TO-modified 2′-OMe–(ORN3) (5′-GCACXCUACG) duplexes at 1.0 µM with DNA targets at 1.1 µM (T_m), with comparison to fully match target (ΔT_m). Position of mismatch refers to a mismatch (Table S1) in the target strand counting from the TO-modified base, e.g. position "0" is directly opposite the TO modification and "-2" is two nucleobases towards the 5′ end of the probe sequence. ΔT_m wasn't referenced as previously to completely unmodified duplexes due to lack of detectable T_m of some of mismatched duplexes, data not shown.

	X =	Position of mismatch	T_m of appropriate DNA target			ΔT_m of appropriate DNA target to match target
2′-OMe–(ORN3) 5′-GCACXCUACG	$AE\text{-}TO_{Q6}$	-2	37.5	±	0.9	14.1
		-1	32.1	±	0.9	19.6
		0	42.4	±	0.2	9.2
		+1	30.0	±	0.7	21.7
		+2	41.2	±	0.3	10.5
		match	51.7	±	0.4	-
	$C6\text{-}TO_{B6}$	-2	30.8	±	0.5	21.7
		-1	24.3	±	0.8	28.2
		0	40.4	±	0.8	12.1
		+1	30.4	±	0.3	22.1
		+2	41.1	±	0.7	11.4
		match	52.5	±	0.6	-

Fluorescence data

Table S8 Fluorescence emssion data for TO-modified ODN1-4 probes against DNA targets. F_{ds}/F_{ss} – ratio of integrated fluorescence emission of oligonucleotide duplexes to single stranded, I_0 – fluorescence emission intensity at $\lambda_{em,\ max}$ of single stranded TO-modified oligonucleotides probes, I_{max} – fluorescence emission intensity at $\lambda_{em,\ max}$ of TO-modified oligonucleotide probe-target duplexes. Mismatch target refers to a T mismatch opposite the TO-modified base.

ODN	X =	DNA target	Average F_{ds}/F_{ss}	Average I_0			Average I_{max}		
ODN1 5′-GCATXTTACG	PA-TO_{Q6}	match	1.0	130.4	±	6.0	126.0	±	8.2
		mismatch	0.9	139.2	±	12.3	118.7	±	8.6
	PA-TO_{B6}	match	3.7	48.4	±	1.7	199.4	±	6.6
		mismatch	1.1	51.2	±	6.4	59.4	±	7.8
	AE-TO_{Q6}	match	2.8	49.8	±	0.3	159.6	±	1.9
		mismatch	1.5	48.4	±	0.7	78.2	±	1.8
	AE-TO_{B6}	match	0.8	30.8	±	1.1	22.4	±	1.2
		mismatch	0.9	31.7	±	0.1	26.2	±	0.9
	C6-TO_{Q6}	match	1.0	99.0	±	8.6	83.3	±	1.4
		mismatch	0.5	111.4	±	7.5	56.8	±	9.6
	C6-TO_{B6}	match	6.6	48.3	±	2.8	393.8	±	21.1
		mismatch	2.3	47.5	±	3.9	120.3	±	7.2
ODN2 5′-GCAAXATACG	PA-TO_{Q6}	match	1.4	14.6	±	0.5	20.3	±	0.9
		mismatch	1.3	14.7	±	0.4	17.4	±	0.8
	PA-TO_{B6}	match	5.5	28.4	±	0.8	179.2	±	4.3
		mismatch	3.8	26.0	±	1.0	101.8	±	0.7
	AE-TO_{Q6}	match	7.4	12.4	±	0.1	102.0	±	0.5
		mismatch	3.2	12.3	±	0.1	38.1	±	1.6
	AE-TO_{B6}	match	1.0	6.5	±	1.7	7.1	±	1.8
		mismatch	1.2	6.3	±	1.5	9.1	±	3.8
	C6-TO_{Q6}	match	3.7	19.4	±	2.7	75.6	±	7.1
		mismatch	2.5	20.2	±	0.5	58.0	±	0.4
	C6-TO_{B6}	match	8.6	21.4	±	1.3	228.4	±	4.2
		mismatch	5.5	19.3	±	1.3	122.4	±	2.7
ODN3 5′-GCACXCTACG	PA-TO_{Q6}	match	1.5	32.1	±	0.5	45.9	±	1.4
		mismatch	2.6	30.8	±	0.1	61.7	±	1.2
	PA-TO_{B6}	match	5.3	11.7	±	1.1	64.2	±	1.4
		mismatch	3.3	12.4	±	1.7	49.5	±	3.2
	AE-TO_{Q6}	match	4.1	33.7	±	0.2	199.9	±	2.7
		mismatch	1.5	34.1	±	0.6	56.8	±	1.1
	AE-TO_{B6}	match	1.9	13.9	±	1.3	24.6	±	1.3
		mismatch	1.3	14.8	±	0.8	17.4	±	0.6
	C6-TO_{Q6}	match	3.1	37.5	±	2.1	116.4	±	4.8
		mismatch	0.9	36.2	±	2.4	33.8	±	4.2
	C6-TO_{B6}	match	6.9	25.7	±	1.6	200.1	±	11.0
		mismatch	2.6	26.8	±	0.3	70.7	±	3.8

ODN4<br>5'-GCAGXGTACG	PA-TO_{Q6}	match	0.9	62.1	±	0.1	54.8	±	0.6
		mismatch	1.3	56.6	±	0.4	60.7	±	0.6
	PA-TO_{B6}	match	2.7	61.9	±	1.3	194.9	±	2.3
		mismatch	1.3	65.9	±	1.8	94.1	±	2.3
	AE-TO_{Q6}	match	1.3	66.2	±	2.0	88.2	±	1.0
		mismatch	0.6	65.0	±	2.5	41.1	±	1.8
	AE-TO_{B6}	match	0.8	120.1	±	3.7	108.0	±	0.6
		mismatch	0.6	121.4	±	3.4	70.7	±	0.8
	C6-TO_{Q6}	match	1.4	49.0	±	7.0	77.3	±	8.4
		mismatch	0.6	48.4	±	7.1	36.0	±	5.2
	C6-TO_{B6}	match	3.0	87.5	±	5.5	332.7	±	1.8
		mismatch	1.1	71.3	±	4.4	86.6	±	14.2
ODN4'<br>5'-GCAIXITACG<br>I = Inosine	C6-TO_{Q6}	match	1.1	47.5	±	0.9	62.7	±	0.4
		mismatch	1.0	46.2	±	1.2	39.6	±	2.0
	C6-TO_{B6}	match	6.2	35.7	±	0.7	273.2	±	8.3
		mismatch	1.1	34.8	±	0.8	62.3	±	2.2

Table S9 Fluorescence emssion data for TO-modified 2′-OMe–(ORN1-4) probes against DNA targets. F_{ds}/F_{ss} – ratio of integrated fluorescence emission of oligonucleotide duplexes to single stranded, I_0 – fluorescence emission intensity at $\lambda_{em, max}$ of single stranded TO-modified oligonucleotides probes, I_{max} – fluorescence emission intensity at $\lambda_{em, max}$ of TO-modified oligonucleotide probe-target duplexes. Mismatch target refers to a T mismatch opposite the TO-modified base.

2′-OMe–(ORN)	X =	DNA target	Average F_{ds}/F_{ss}	Average I_0	Average I_{max}
2′-OMe–(ORN1) 5′-GCAUXUUACG	AE-TO_{Q6}	match	21.5	16.2 ± 1.5	472.8 ± 57.8
		mismatch	8.9	15.2 ± 0.9	169.4 ± 3.8
	AE-TO_{B6}	match	1.3	24.9 ± 0.5	31.7 ± 2.1
		mismatch	1.6	26.7 ± 0.4	41.0 ± 1.8
	C6-TO_{Q6}	match	4.2	20.4 ± 2.0	81.6 ± 2.2
		mismatch	1.7	22.5 ± 2.3	36.1 ± 3.8
	C6-TO_{B6}	match	14.6	14.5 ± 1.3	270.7 ± 17.0
		mismatch	2.7	15.1 ± 0.9	45.3 ± 7.2
	AE-TO_{Q10}	match	21.8	9.6 ± 4.0	273.5 ± 3.5
		mismatch	17.0	9.6 ± 4.0	193.2 ± 9.0
	AE-TO_{B10}	match	1.9	4.8 ± 0.01	8.6 ± 0.1
		mismatch	no data obtained/measurement wasn't performed		
	C6-TO_{Q10}	match	2.6	15.2 ± 0.1	41.4 ± 1.7
		mismatch	1.3	15.2 ± 0.1	19.4 ± 1.9
	C6-TO_{B10}	match	7.6	27.6 ± 0.5	241.4 ± 34.5
		mismatch	1.7	27.6 ± 0.5	50.1 ± 0.6
2′-OMe– 5′-GCAXUUXACG	2× AE-TO_{Q6}	match	14.1	9.5 ± 0.5	239.3 ± 22.1
		mismatch	9.0	9.5 ± 0.6	128.7 ± 4.2
2′-OMe–(ORN2) 5′-GCAAXAUACG	AE-TO_{Q6}	match	7.6	44.9 ± 1.7	450.8 ± 15.0
		mismatch	3.0	46.0 ± 1.0	172.1 ± 1.4
	AE-TO_{B6}	match	0.9	43.2 ± 1.4	34.5 ± 1.2
		mismatch	1.6	44.1 ± 1.7	78.1 ± 4.9
	C6-TO_{Q6}	match	9.1	11.0 ± 0.2	114.6 ± 2.9
		mismatch	2.8	11.3 ± 0.2	34.2 ± 0.5
	C6-TO_{B6}	match	17.5	11.2 ± 0.4	226.9 ± 7.2
		mismatch	8.6	10.9 ± 0.4	119.1 ± 0.8
2′-OMe–(ORN3) 5′-GCACXCUACG	AE-TO_{Q6}	match	33.9	9.3 ± 0.1	425.4 ± 8.6
		mismatch	17.0	9.7 ± 0.2	192.2 ± 1.9
	AE-TO_{B6}	match	3.5	12.1 ± 0.2	41.0 ± 1.2
		mismatch	2.6	11.8 ± 0.4	30.4 ± 0.5
	C6-TO_{Q6}	match	6.4	9.3 ± 1.1	58.4 ± 5.1
		mismatch	2.0	10.6 ± 0.4	20.1 ± 0.6
	C6-TO_{B6}	match	17.2	12.5 ± 1.4	251.7 ± 30.9
		mismatch	3.9	15.8 ± 3.2	65.4 ± 10.6
2′-OMe–(ORN4) 5′-GCAGXGUACG	AE-TO_{Q6}	match	3.1	90.2 ± 1.0	355.6 ± 1.7
		mismatch	1.4	96.4 ± 2.0	167.0 ± 0.8
	AE-TO_{B6}	match	0.2	226.8 ± 3.2	56.1 ± 16.5
		mismatch	0.3	239.6 ± 4.8	79.5 ± 0.8
	C6-TO_{Q6}	match	1.0	52.3 ± 6.0	55.2 ± 6.3
		mismatch	0.7	64.4 ± 0.8	42.9 ± 1.7
	C6-TO_{B6}	match	3.7	94.3 ± 3.1	420.5 ± 5.4
		mismatch	1.2	87.1 ± 1.1	107.3 ± 3.5

Table S10 Fluorescence emssion data for TO-modified ODN1-4 probes against RNA targets. F_{ds}/F_{ss} – ratio of integrated fluorescence emission of oligonucleotide duplexes to single stranded, I_0 – fluorescence emission intensity at $\lambda_{em,\ max}$ of single stranded TO-modified oligonucleotides probes, I_{max} – fluorescence emission intensity at $\lambda_{em,\ max}$ of TO-modified oligonucleotide probe-targetduplexes. Mismatch target refers to a U mismatch opposite the TO-modified base.

ODN	X =	RNA target	Average F_{ds}/F_{ss}	Average I_0			Average I_{max}		
ODN1 5′-GCATXTTACG	AE-TO$_{Q6}$	match	2.5	49.5	±	0.2	127.0	±	2.1
		mismatch	1.0	48.2	±	0.3	47.1	±	0.9
	AE-TO$_{B6}$	match	2.5	30.5	±	0.4	76.1	±	0.2
		mismatch	1.3	30.5	±	0.2	41.0	±	1.6
	C6-TO$_{Q6}$	match	1.6	115.0	±	7.6	171.6	±	16.6
		mismatch	1.0	102.2	±	0.7	100.2	±	3.4
	C6-TO$_{B6}$	match	6.4	53.1	±	4.1	404.0	±	24.8
		mismatch	2.0	48.4	±	0.6	99.7	±	2.8
ODN2 5′-GCAAXATACG	AE-TO$_{Q6}$	match	5.2	14.0	±	0.1	76.2	±	7.4
		mismatch	1.8	14.0	±	0.1	26.7	±	2.4
	AE-TO$_{B6}$	match	3.3	6.2	±	1.6	17.0	±	3.8
		mismatch	1.2	7.8	±	1.5	10.0	±	3.5
	C6-TO$_{Q6}$	match	2.8	13.6	±	0.3	37.1	±	5.4
		mismatch	1.3	16.6	±	1.7	17.2	±	1.4
	C6-TO$_{B6}$	match	7.2	13.9	±	0.1	111.5	±	8.9
		mismatch	2.6	14.8	±	0.4	40.7	±	1.3
ODN3 5′-GCACXCTACG	AE-TO$_{Q6}$	match	3.8	34.5	±	1.2	139.1	±	0.3
		mismatch	1.8	34.5	±	1.0	61.3	±	1.0
	AE-TO$_{B6}$	match	3.8	18.8	±	1.4	77.3	±	2.6
		mismatch	3.7	18.9	±	1.5	76.3	±	5.3
	C6-TO$_{Q6}$	match	2.3	23.9	±	6.8	56.7	±	0.1
		mismatch	2.6	24.0	±	7.2	68.7	±	3.4
	C6-TO$_{B6}$	match	5.2	28.0	±	0.6	158.5	±	6.8
		mismatch	3.1	28.1	±	0.2	90.2	±	2.9
ODN4 5′-GCAGXGTACG	AE-TO$_{Q6}$	match	2.2	65.0	±	3.1	150.2	±	4.0
		mismatch	1.1	66.2	±	2.2	71.1	±	3.0
	AE-TO$_{B6}$	match	1.0	112.3	±	4.2	118.3	±	7.9
		mismatch	0.8	112.3	±	4.4	93.7	±	2.8
	C6-TO$_{Q6}$	match	1.8	37.9	±	8.9	69.8	±	3.7
		mismatch	1.0	38.4	±	8.0	41.4	±	8.8
	C6-TO$_{B6}$	match	2.8	73.7	±	14.6	196.0	±	9.9
		mismatch	1.4	73.5	±	17.4	85.9	±	6.8
ODN4′ 5′-GCAIXITACG I = Inosine	C6-TO$_{Q6}$	match	1.1	47.6	±	0.9	63.1	±	0.3
		mismatch	0.9	46.3	±	1.2	47.2	±	3.0
	C6-TO$_{B6}$	match	3.1	35.8	±	0.7	119.1	±	7.6
		mismatch	1.0	34.7	±	0.8	44.8	±	1.5

Table S11 Fluorescence emssion data for TO-modified 2′-OMe–(ORN1-4) probes against RNA targets. F_{ds}/F_{ss} – ratio of integrated fluorescence emission of oligonucleotide duplexes to single stranded, I_0 – fluorescence emission intensity at $\lambda_{em,\ max}$ of single stranded TO-modified oligonucleotides probes, I_{max} – fluorescence emission intensity at $\lambda_{em,\ max}$ of TO-modified oligonucleotide probe-target duplexes. Mismatch target refers to a U mismatch opposite the TO-modified base.

2′-OMe–(ORN)	X =	RNA target	Average F_{ds}/F_{ss}	Average I_0	Average I_{max}
2′-OMe–(ORN1) 5′-GCAUXUUACG	AE-TO_{Q6}	match	9.8	14.1 ± 1.6	158.4 ± 9.2
		mismatch	4.8	15.4 ± 3.0	79.5 ± 10.7
	AE-TO_{B6}	match	2.7	24.3 ± 1.7	66.2 ± 12.0
		mismatch	1.6	23.1 ± 1.9	35.6 ± 2.4
	C6-TO_{Q6}	match	5.0	20.3 ± 0.3	94.7 ± 4.1
		mismatch	3.1	20.9 ± 2.1	65.5 ± 3.8
	C6-TO_{B6}	match	15.8	13.4 ± 0.4	254.7 ± 17.6
		mismatch	5.1	15.2 ± 1.1	80.4 ± 2.0
	AE-TO_{Q10}	match	16.0	9.6 ± 4.0	282.7 ± 26.1
		mismatch	11.1	9.6 ± 4.0	108.4 ± 5.1
	AE-TO_{B10}	match	4.2	8.7 ± 0.2	31.4 ± 1.0
		mismatch	no data obtained/measurement wasn’t performed		
	C6-TO_{Q10}	match	4.2	17.8 ± 4.2	68.2 ± 6.2
		mismatch	3.1	15.2 ± 0.1	51.0 ± 1.7
	C6-TO_{B10}	match	8.5	27.6 ± 0.6	246.0 ± 4.8
		mismatch	2.9	27.7 ± 0.5	76.8 ± 0.1
2′-OMe– 5′-GCAXUUXACG	2× AE-TO_{Q6}	match	8.2	9.7 ± 0.7	126.2 ± 3.6
		mismatch	7.0	9.5 ± 0.5	133.4 ± 2.5
2′-OMe–(ORN2) 5′-GCAAXAUACG	AE-TO_{Q6}	match	3.0	46.0 ± 1.0	196.3 ± 21.9
		mismatch	0.8	44.9 ± 1.7	63.2 ± 1.7
	AE-TO_{B6}	match	1.2	44.9 ± 0.8	53.4 ± 2.4
		mismatch	1.1	44.1 ± 0.8	45.1 ± 3.2
	C6-TO_{Q6}	match	8.9	11.6 ± 1.3	117.9 ± 30.1
		mismatch	3.5	12.0 ± 0.4	49.6 ± 1.4
	C6-TO_{B6}	match	11.3	11.2 ± 0.5	136.8 ± 16.3
		mismatch	5.1	12.1 ± 1.7	58.5 ± 3.5
2′-OMe–(ORN3) 5′-GCACXCUACG	AE-TO_{Q6}	match	12.7	9.5 ± 0.4	138.9 ± 1.3
		mismatch	8.4	9.5 ± 0.2	84.9 ± 17.4
	AE-TO_{B6}	match	2.5	11.8 ± 0.2	30.3 ± 1.4
		mismatch	4.7	11.6 ± 0.1	58.8 ± 2.5
	C6-TO_{Q6}	match	3.2	12.0 ± 0.2	38.0 ± 0.3
		mismatch	2.4	12.2 ± 0.2	27.5 ± 0.1
	C6-TO_{B6}	match	6.3	14.9 ± 0.5	101.1 ± 2.6
		mismatch	4.3	14.1 ± 0.2	65.2 ± 2.7
2′-OMe–(ORN4) 5′-GCAGXGUACG	AE-TO_{Q6}	match	3.4	98.4 ± 6.7	408.1 ± 7.0
		mismatch	1.0	119.4 ± 11.2	129.9 ± 2.8
	AE-TO_{B6}	match	0.5	232.4 ± 8.8	108.3 ± 3.3
		mismatch	0.3	227.9 ± 11.7	64.4 ± 5.0
	C6-TO_{Q6}	match	1.0	60.1 ± 1.3	57.3 ± 5.3
		mismatch	0.8	62.7 ± 1.3	48.8 ± 2.2
	C6-TO_{B6}	match	2.4	90.4 ± 1.7	246.3 ± 7.3
		mismatch	1.1	88.7 ± 0.2	93.6 ± 2.2

Table S12 Fluorescence emssion data for TO-modified 2′-OMe–(ORN3) (5′-GCACXCUACG) probe against DNA targets with a range of positional mismatches. F_{ds}/F_{ss} – ratio of integrated fluorescence emission of oligonucleotide probe-target duplexes to single stranded probe, I_0 –fluorescence emission intensity at $\lambda_{em,\ max}$ of single stranded TO-modified oligonucleotides probes, I_{max} – fluorescence emission intensity at $\lambda_{em,\ max}$ of TO-modified oligonucleotide probe-target duplexes. Position of mismatch refers to a mismatch (Table S1) in the target strand counting from the TO-modified base, e.g.: position "0" is directly opposite modification and "-2" is two nucleobases towards 5′ end of the probe sequence. For mismatch values at position "0" or fully matched target: see Table S9.

	X =	Position of mismatch	Average F_{ds}/F_{ss}	Average I_0			Average I_{max}		
2′-OMe–(ORN3) 5′-GCACXCUACG	AE-TO_{Q6}	-2	9.2	8.1	±	0.2	86.2	±	1.2
		-1	22.0	7.6	±	0.2	217.1	±	1.6
		+1	16.5	8.1	±	0.1	178.4	±	1.5
		+2	28.3	8.0	±	0.3	295.3	±	3.1
	C6-TO_{B6}	-2	12.8	12.8	±	0.01	206.6	±	1.1
		-1	4.6	12.7	±	0.1	69.7	±	1.1
		+1	5.8	11.8	±	1.1	73.2	±	1.2
		+2	13.8	9.3	±	0.5	156.2	±	5.9

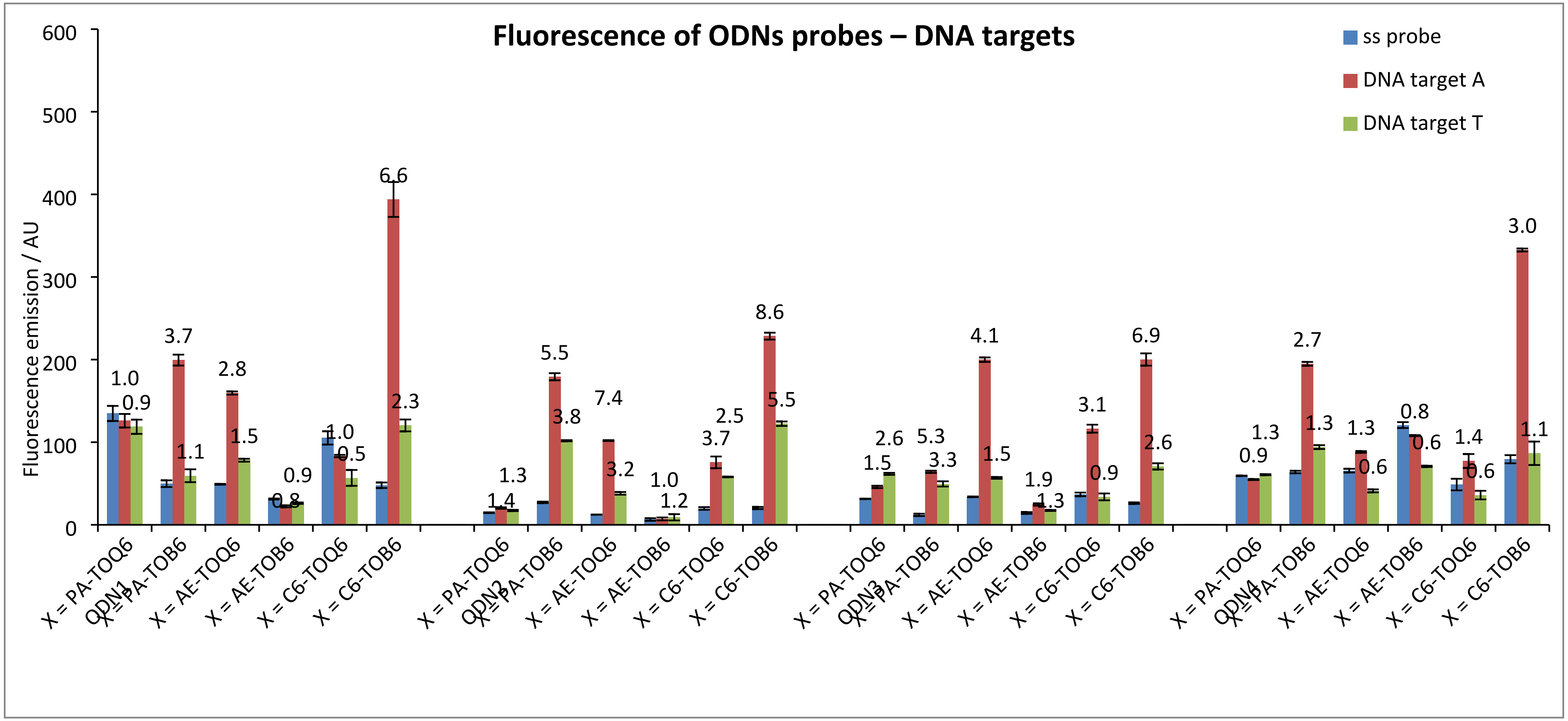

Figure S5 Fluorescence emssion intensity at $\lambda_{em,\ max}$ for TO-modified ODNs (ODN1 (5′-GCATXTTACG), ODN2 (5′-GCAAXATACG), ODN3 (5′-GCACXCTACG), ODN4 (5′-GCAGXGTACG)) probes against DNA targets. The base opposite the TO is either A – match or T – mismatch, ss probe – fluorescence emission intensity of single stranded probe, DNA target A/T - fluorescence emission intensity of probe-target duplex, AU - arbitrary units, number above bar = F_{ds}/F_{ss}. Detailed values: see Table S8.

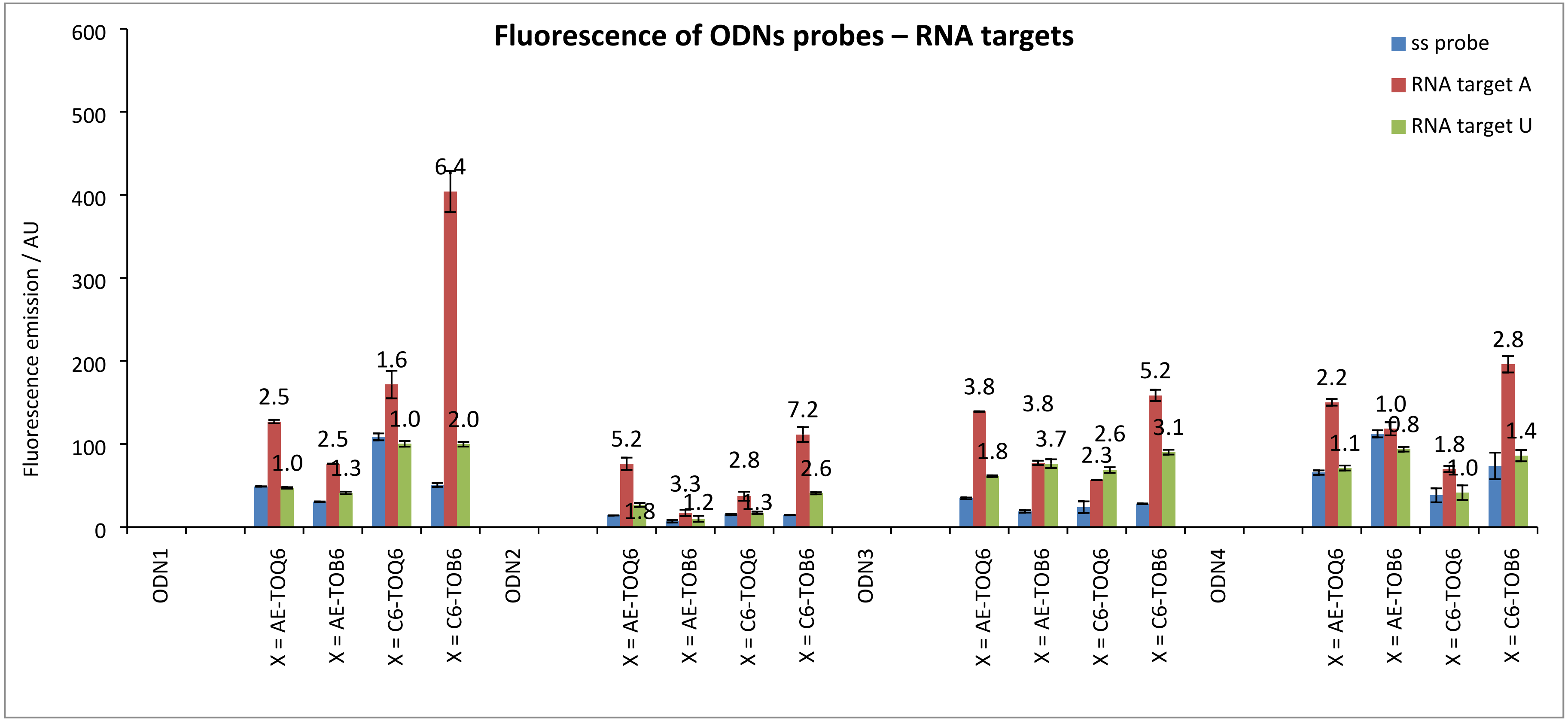

Figure S6 Fluorescence emssion intensity at $\lambda_{em,\ max}$ for TO-modified ODNs (ODN1 (5'-GCATXTTACG), ODN2 (5'-GCAAXATACG), ODN3 (5'-GCACXCTACG), ODN4 (5'-GCAGXGTACG)) probes against 2'-OH–RNA targets. The base opposite the TO is either A – match or U – mismatch, ss probe – fluorescence emission intensity of single stranded probe, RNA target A/U - fluorescence emission intensity of probe-target duplex, AU - arbitrary units, number above bar = F_{ds}/F_{ss}. Detailed values: see Table S10.

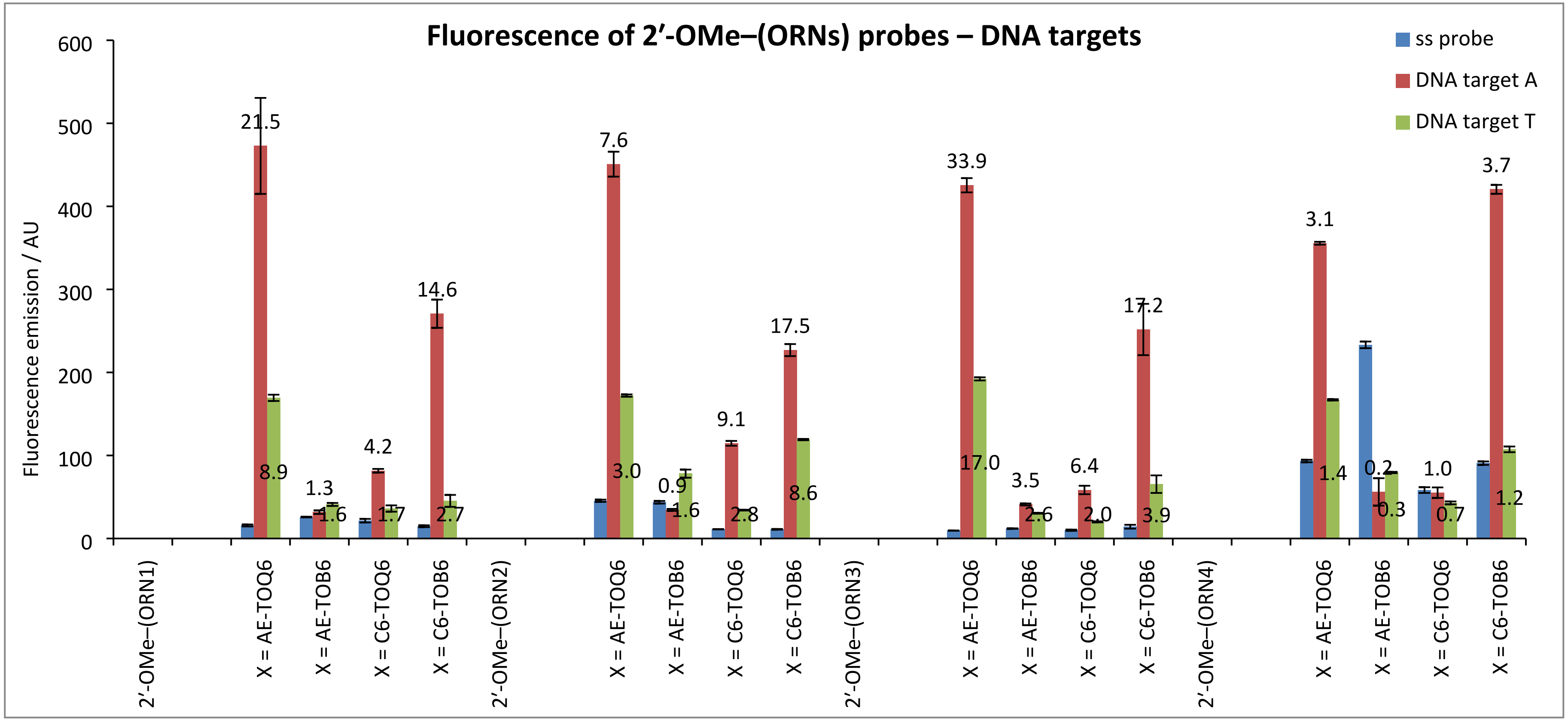

Figure S7 Fluorescence emssion intensity at $\lambda_{em,\ max}$ for TO-modified 2′-OMe–(ORNs) (2′-OMe–(ORN1) (5′-GCAUXUUACG), 2′-OMe–(ORN2) (5′-GCAAXAUACG), 2′-OMe–(ORN3) (5′-GCACXCUACG), 2′-OMe–(ORN4) (5′-GCAGXGUACG)) probes against DNA targets. The base opposite the TO is either A – match or T – mismatch, ss probe – fluorescence emission intensity of single stranded probe, DNA target A/T - fluorescence emission intensity of probe-target duplex, AU - arbitrary units, number above bar = F_{ds}/F_{ss}. Detailed values: see Table S9.

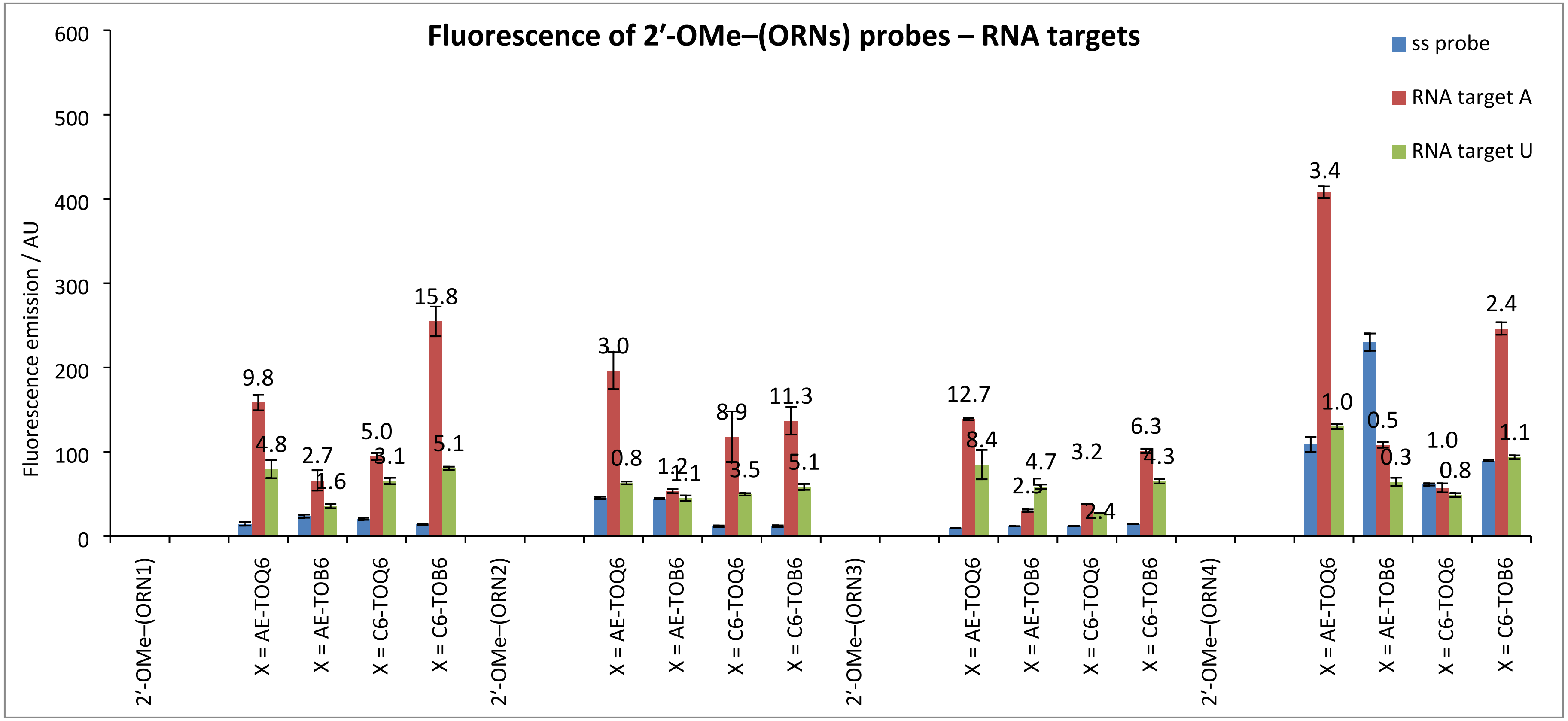

Figure S8 Fluorescence emssion intensity at $\lambda_{em,\ max}$ for TO-modified 2′-OMe–(ORNs) (2′-OMe–(ORN1) (5′-GCAUXUUACG), 2′-OMe–(ORN2) (5′-GCAAXAUACG), 2′-OMe–(ORN3) (5′-GCACXCUACG), 2′-OMe–(ORN4) (5′-GCAGXGUACG)) probes against RNA targets. The base opposite the TO is either A – match or U – mismatch, ss probe – fluorescence emission intensity of single stranded probe, RNA target A/U - fluorescence emission intensity of probe-target duplex, AU - arbitrary units, number above bar = F_{ds}/F_{ss}. Detailed values: see Table S11.

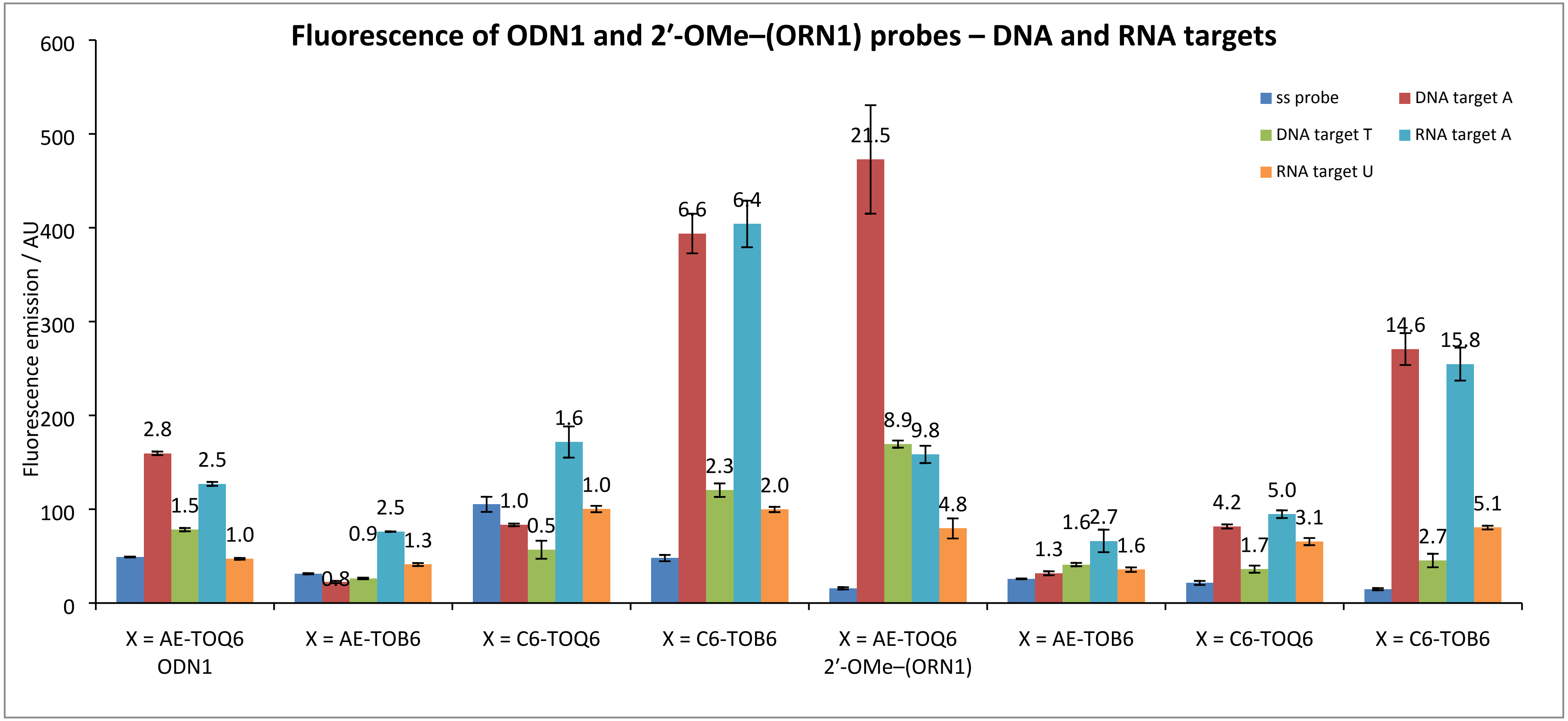

Figure S9 Fluorescence emssion intensity at $\lambda_{em,\ max}$ for TO-modified ODN1 (5'-GCATXTTACG) and 2'-OMe–(ORN1) (5'-GCAUXUUACG) probes against DNA and RNA targets. The base opposite the TO is either A – match or T/U – mismatch, ss probe – fluorescence emission intensity of single stranded probe, DNA target A/T and RNA target A/U - fluorescence emission intensity of probe-target duplex, AU - arbitrary units, number above bar = F_{ds}/F_{ss}. Detailed values: see Table S8-11.

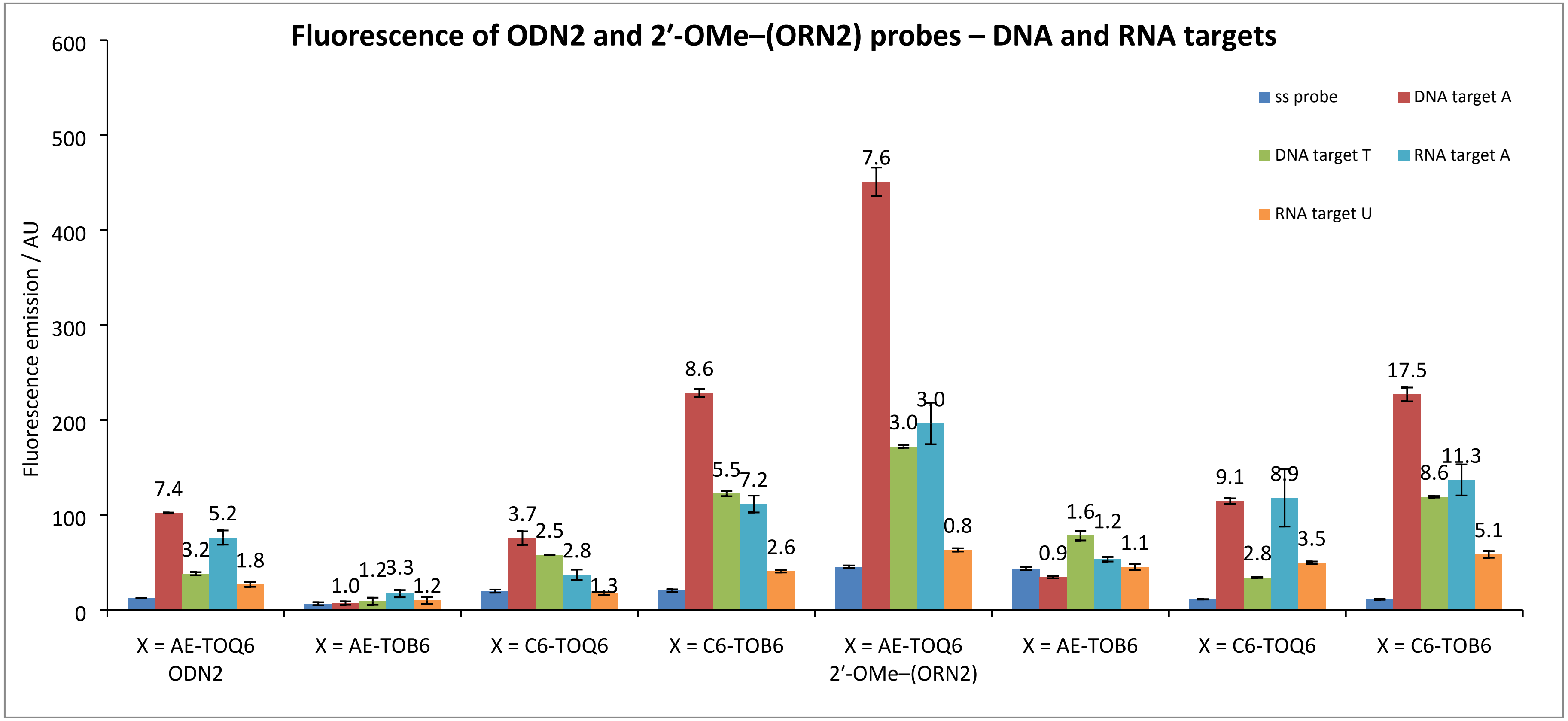

Figure S10 Fluorescence emssion intensity at $\lambda_{em,\ max}$ for TO-modified ODN2 (5′-GCAAXATACG) and 2′-OMe–(ORN2) (5′-GCAAXAUACG) probes against DNA and RNA targets. The base opposite the TO is either A – match or T/U – mismatch, ss probe – fluorescence emission intensity of single stranded probe, DNA target A/T and RNA target A/U - fluorescence emission intensity of probe-target duplex, AU - arbitrary units, number above bar = F_{ds}/F_{ss}. Detailed values: see Table S8-11.

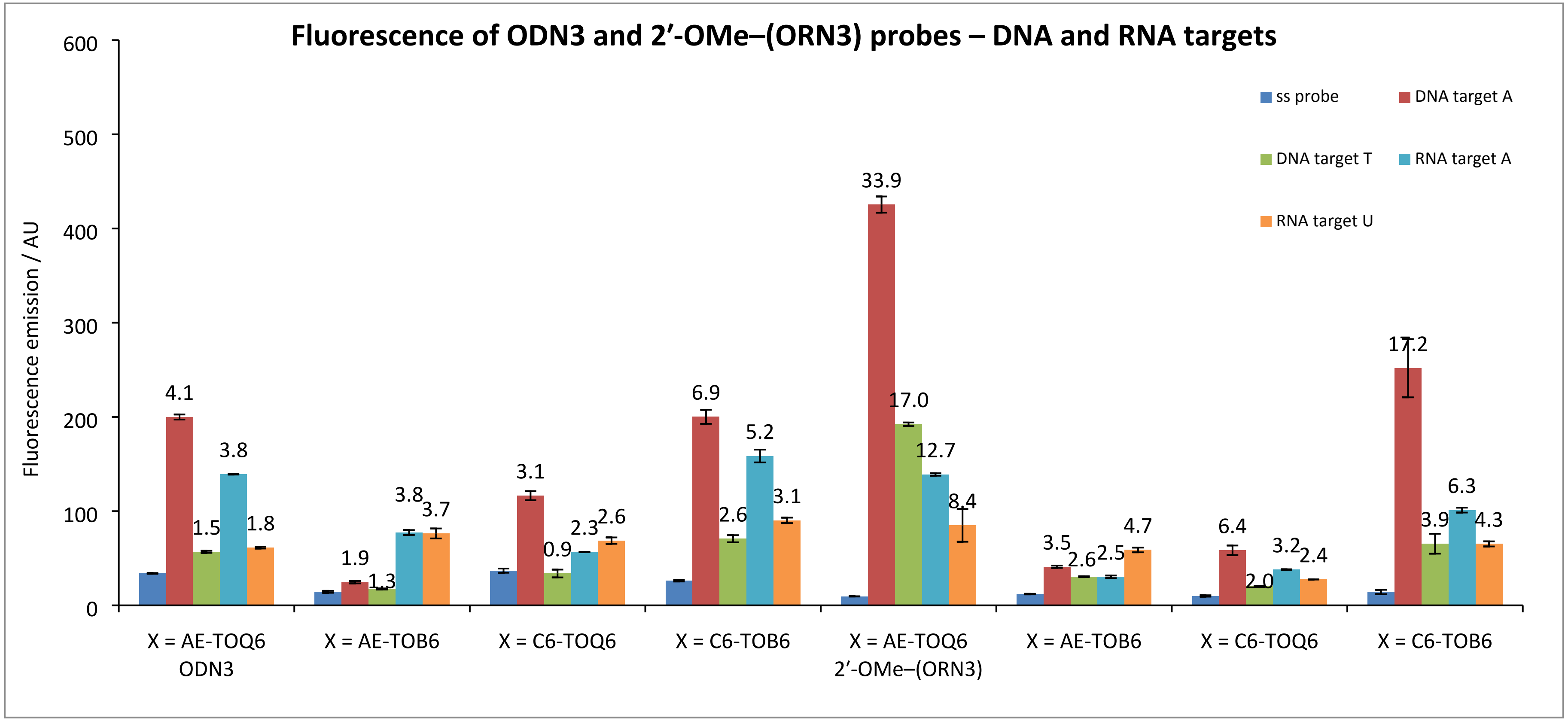

Figure S11 Fluorescence emssion intensity at $\lambda_{em,\ max}$ for TO-modified ODN3 (5′-GCACXCTACG) and 2′-OMe–(ORN3) (5′-GCACXCUACG) probes against DNA and RNA targets. The base opposite the TO is either A – match or T/U – mismatch, ss probe – fluorescence emission intensity of single stranded probe, DNA target A/T and RNA target A/U - fluorescence emission intensity of probe-target duplex, AU - arbitrary units, number above bar = F_{ds}/F_{ss}. Detailed values: see Table S8-11.

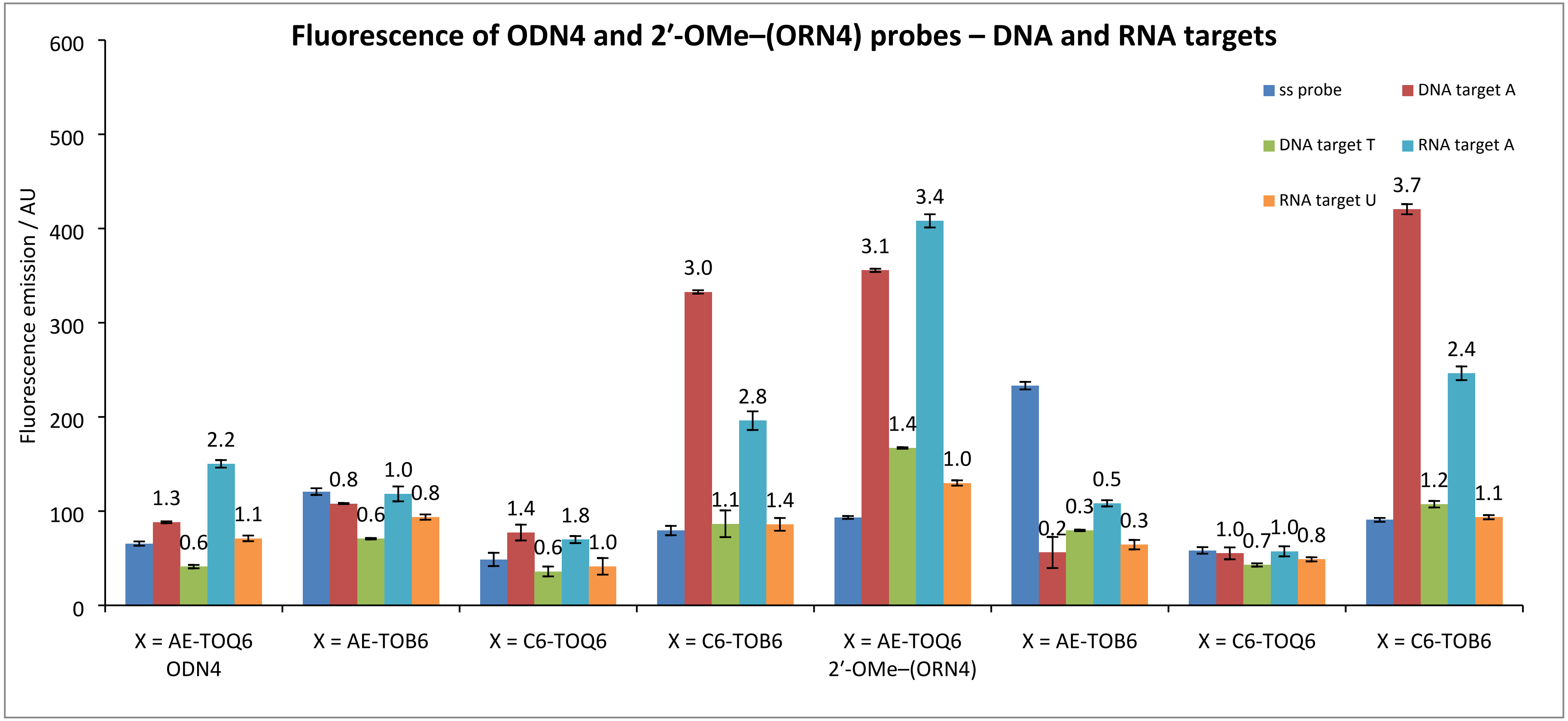

Figure S12 Fluorescence emssion intensity at $\lambda_{em,\ max}$ for TO-modified ODN4 (5′-GCAGXGTACG) and 2′-OMe–(ORN4) (5′-GCAGXGUACG) probes against DNA and RNA targets. The base opposite the TO is either A – match or T/U – mismatch, ss probe – fluorescence emission intensity of single stranded probe, DNA target A/T and RNA target A/U - fluorescence emission intensity of probe-target duplex, AU - arbitrary units, number above bar = F_{ds}/F_{ss}. Detailed values: see Table S8-11.

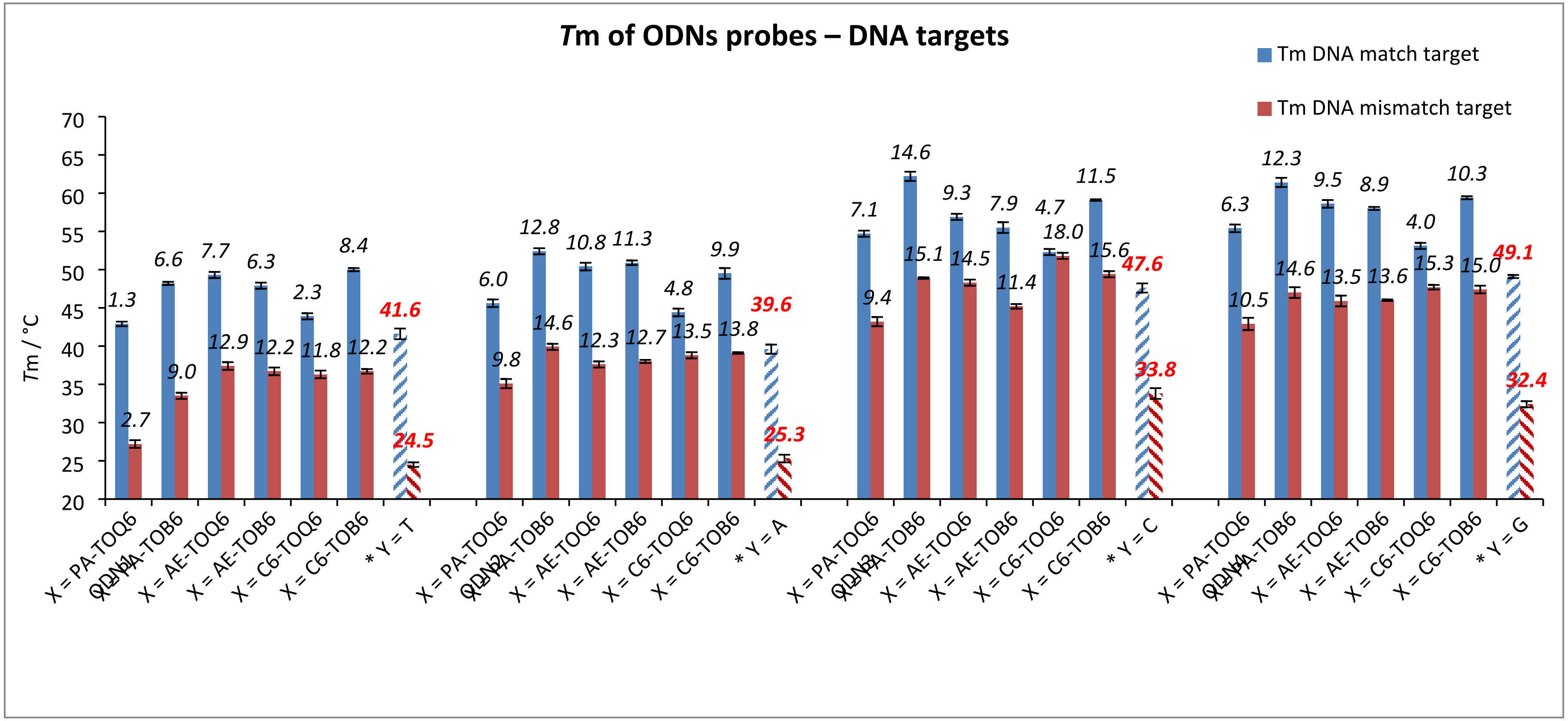

Figure S13 UV melting data for ODNs (ODN1 (5′-GCATXTTACG), ODN2 (5′-GCAAXATACG), ODN3 (5′-GCACXCTACG), ODN4 (5′-GCAGXGTACG)) probes against DNA targets. The base opposite the TO is either A – match or T – mismatch, number above bar = ΔT_m to unmodified target, * = T_m value of unmodified matched and mismatched duplexes. Detailed values: see Table S3.

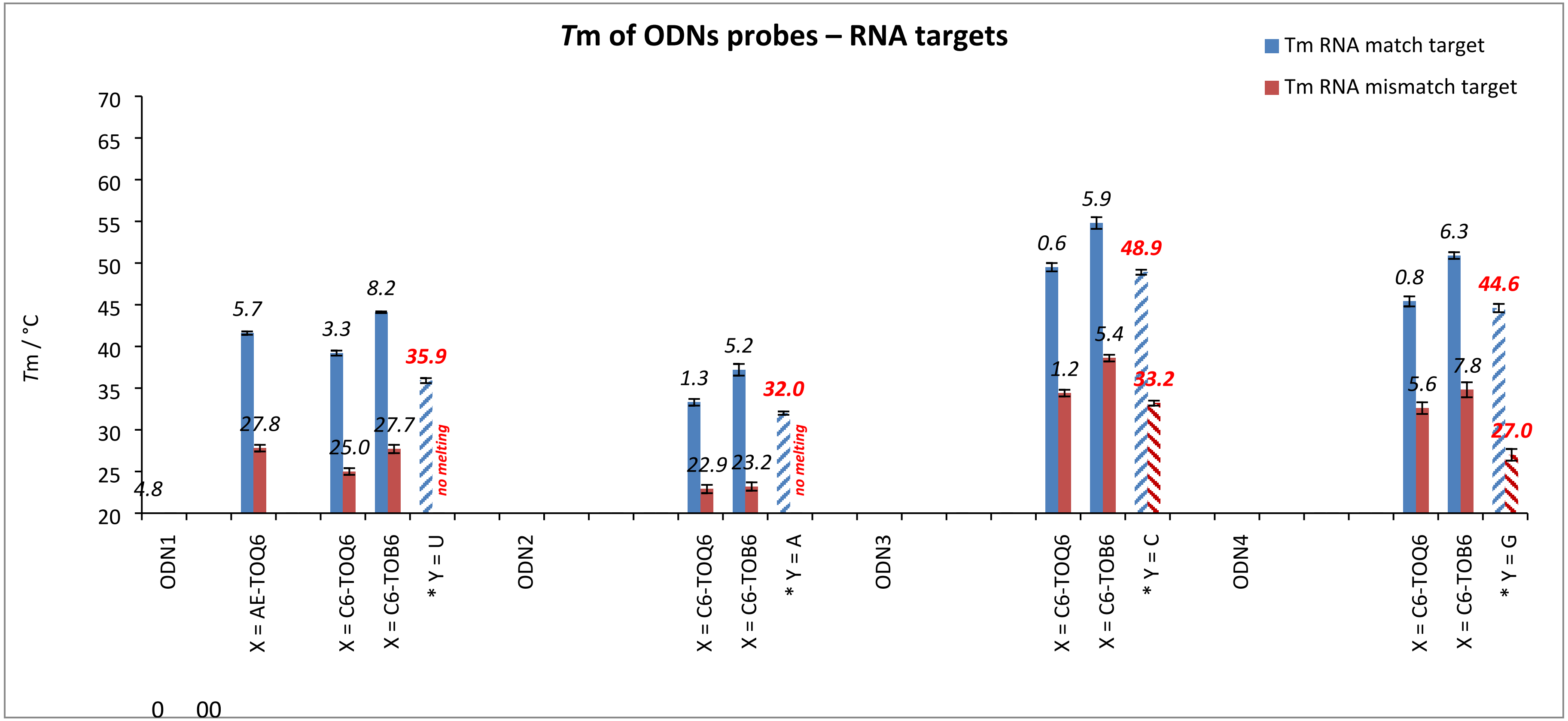

Figure S14 UV melting data for ODNs (ODN1 (5'-GCATXTTACG), ODN2 (5'-GCAAXATACG), ODN3 (5'-GCACXCTACG), ODN4 (5'-GCAGXGTACG)) probes against RNA targets. The base opposite the TO is either A – match or U – mismatch, number above bar = ΔT_m to unmodified target, * = T_m value of unmodified matched and mismatched duplexes. Detailed values: see Table S5.

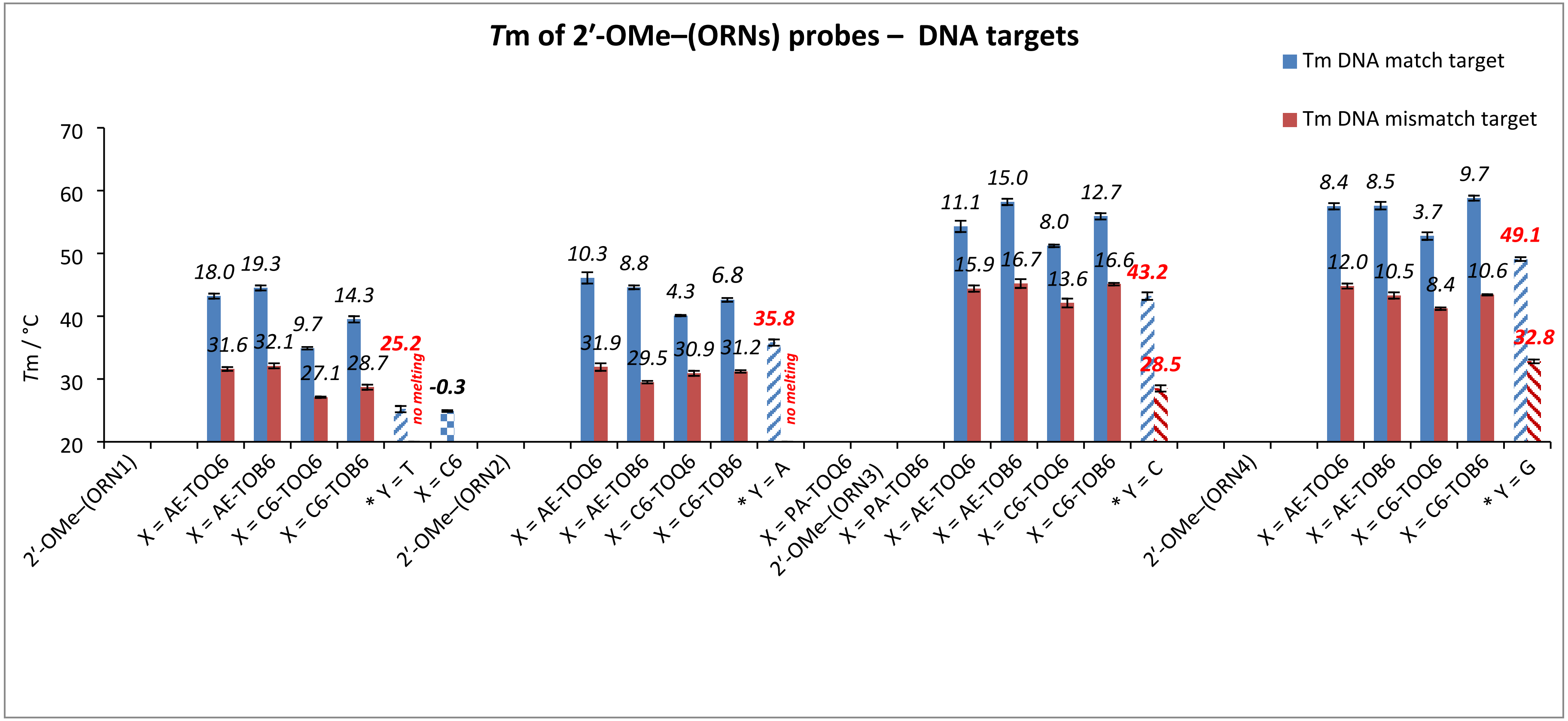

Figure S15 UV melting data for 2′-OMe–(ORNs) (2′-OMe–(ORN1) (5′-GCAUXUUACG), 2′-OMe–(ORN2) (5′-GCAAXAUACG), 2′-OMe–(ORN3) (5′-GCACXCUACG), 2′-OMe–(ORN4) (5′-GCAGXGUACG)) probes against DNA targets. The base opposite the TO is either A – match or T – mismatch, number above bar = ΔT_m to unmodified target, * = T_m value of unmodified matched and mismatched duplexes. Detailed values: see Table S4.

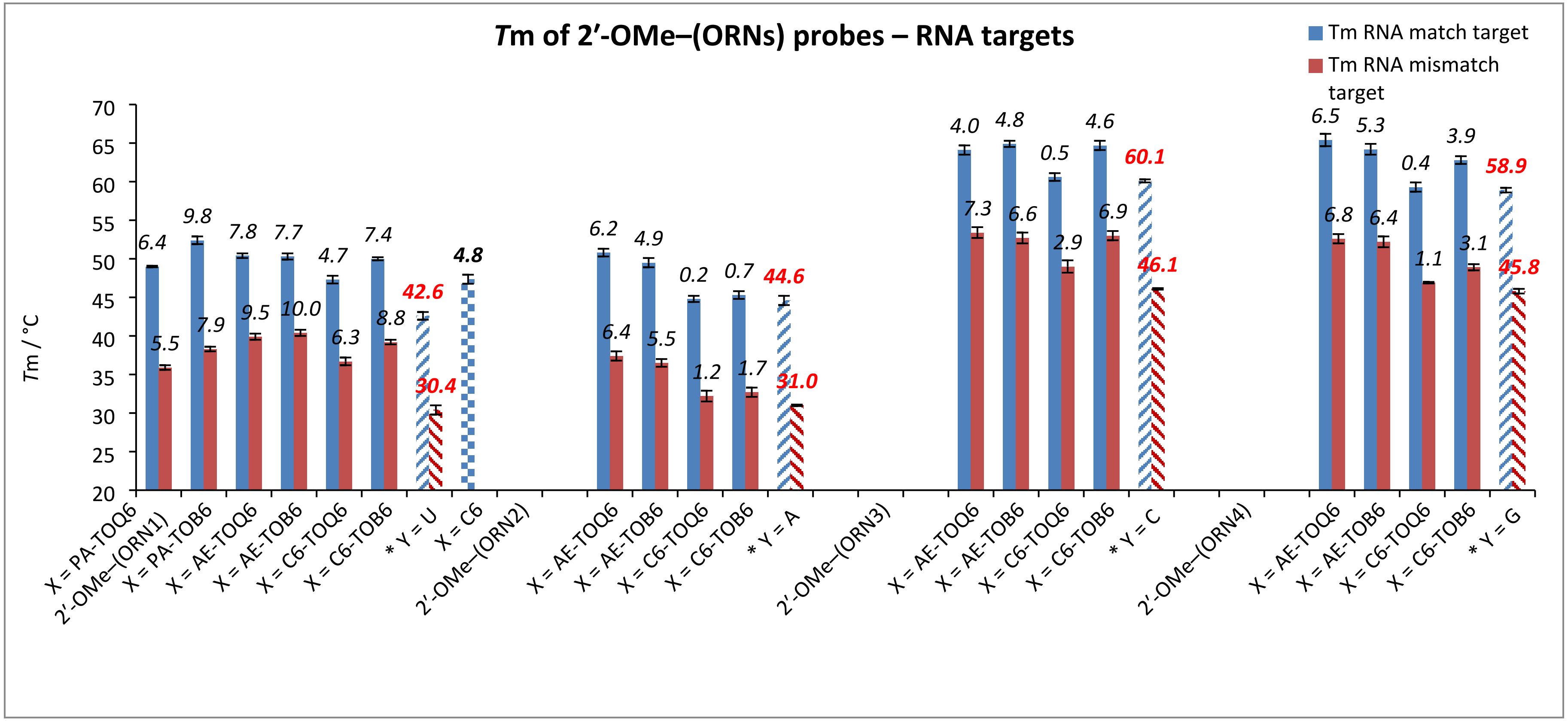

Figure S16 UV melting data for 2′-OMe–(ORNs) (2′-OMe–(ORN1) (5′-GCAUXUUACG), 2′-OMe–(ORN2) (5′-GCAAXAUACG), 2′-OMe–(ORN3) (5′-GCACXCUACG), 2′-OMe–(ORN4) (5′-GCAGXGUACG)) probes against RNA targets. The base opposite the TO is either A – match or U – mismatch, number above bar = ΔT_m to unmodified target, * = T_m value of unmodified matched and mismatched duplexes. Detailed values: see Table S6.

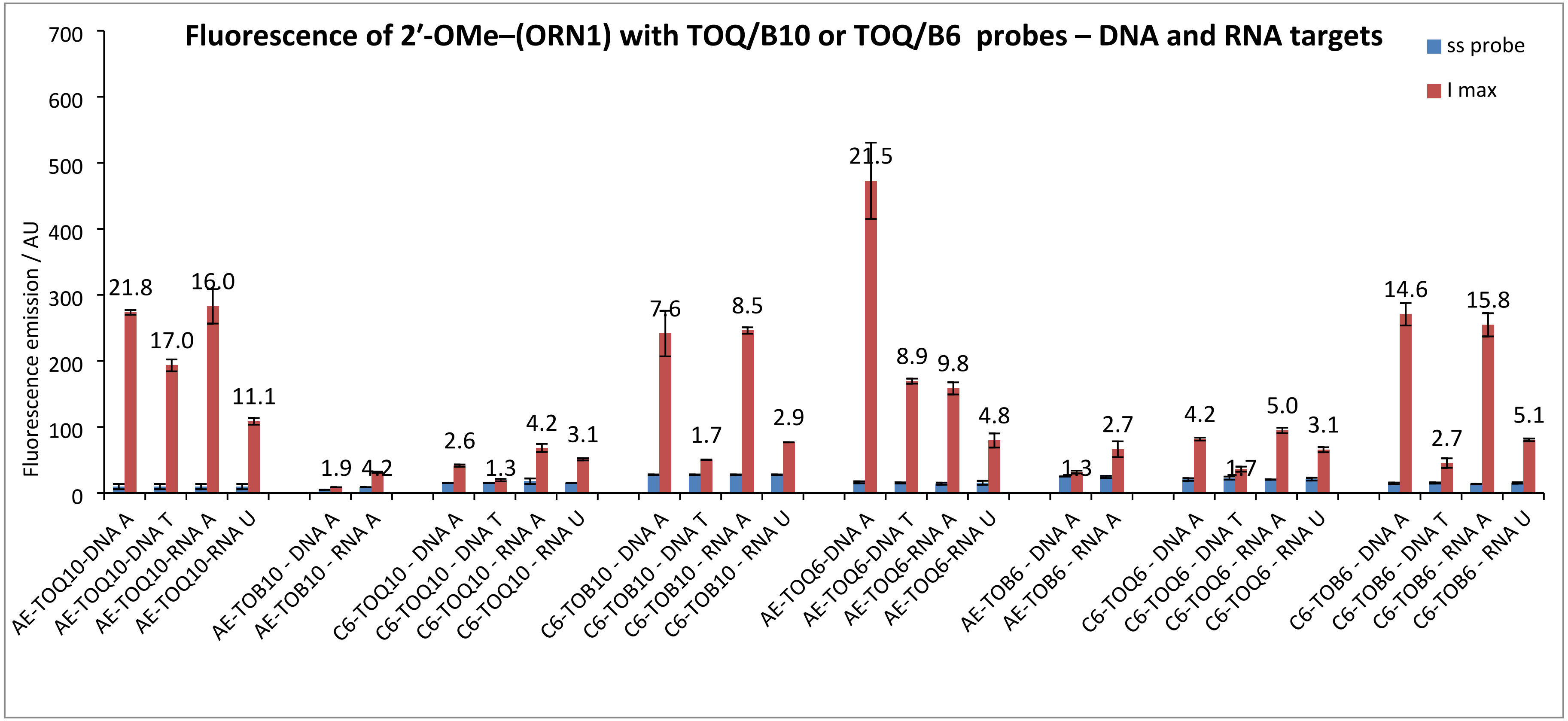

Figure S17 Comparison of fluorescence emssion intensity at $\lambda_{em,\ max}$ data for 2′-OMe–(ORN1) (5′-GCAUXUUACG) with $TO_{B/Q6}$ or $TO_{B/Q10}$ probes against DNA and RNA targets. The base opposite the TO is either A – match or T/U – mismatch, ss probe – fluorescence emission intensity of single stranded probe at $\lambda_{em,\ max}$, I_{max} – fluorescence emission intensity of probe-targetduplex at $\lambda_{em,\ max}$, AU - arbitrary units, number above bar = F_{ds}/F_{ss}. Detailed values: see Table S9 and S11.

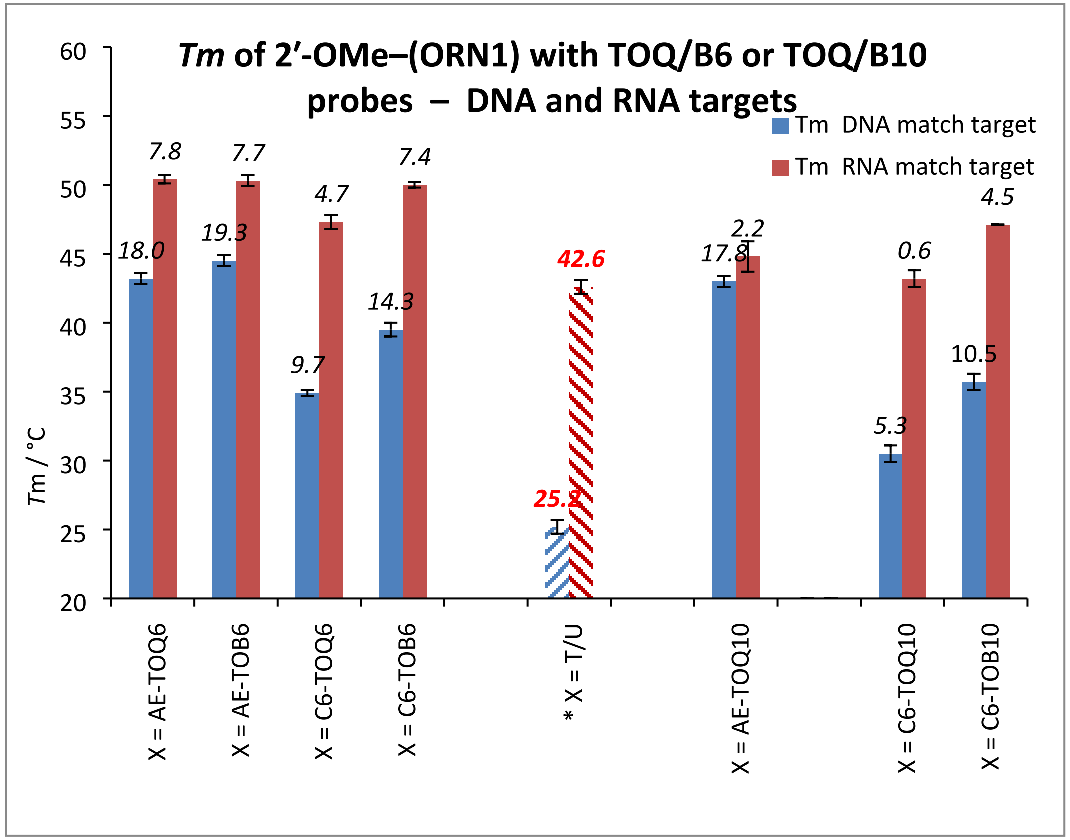

Figure S18 UV melting data for 2′-OMe–(ORN1) (5′-GCAUXUUACG) with $TO_{B/Q6}$ and $TO_{B/Q10}$ probes against DNA and RNA targets. The base opposite the TO is either A – match or T/U – mismatch, number above bar = ΔT_m to unmodified target, * = T_m value of unmodified matched and mismatched duplexes. Detailed values: see Table S4 and S6.

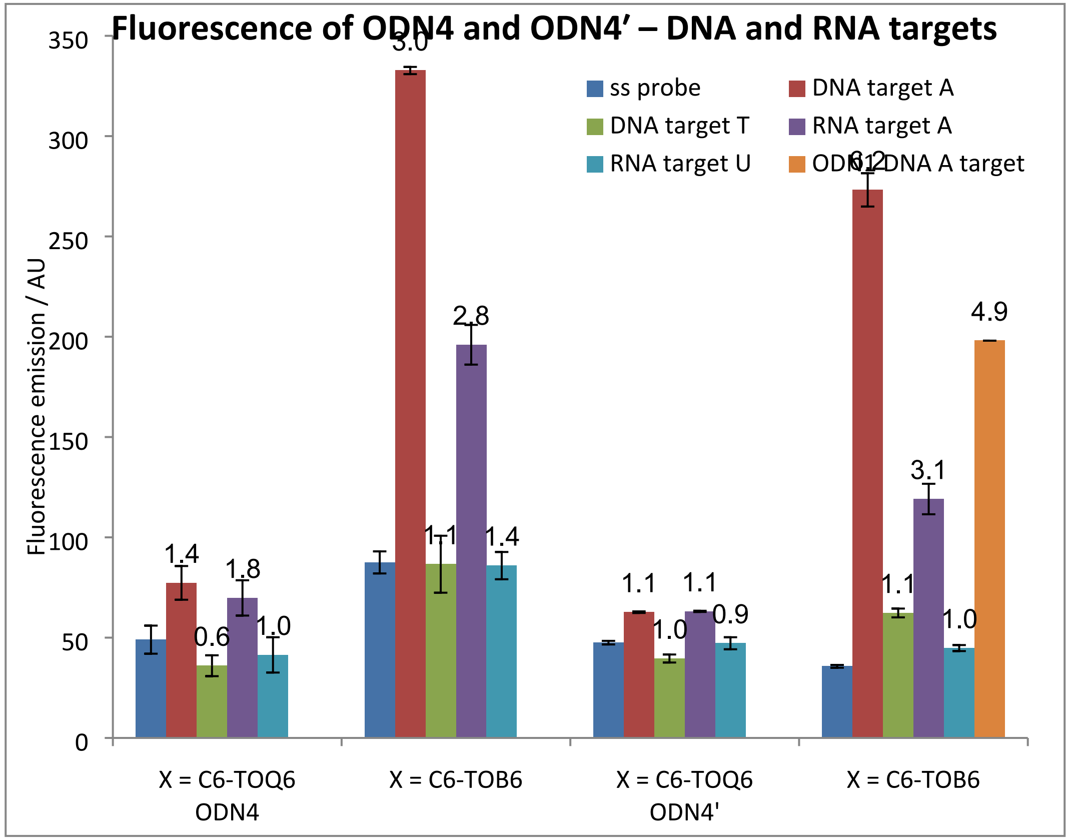

Figure S19 Comparison of fluorescence emssion intensity at $\lambda_{em,\ max}$ data for ODN4 (5'-GCAG**X**GTACG) and ODN4' (5'-GCAI**X**ITACG) with $TO_{B/Q6}$ probes against DNA and RNA targets. The base opposite the TO is either A – match or T/U – mismatch, ss probe – fluorescence emission intensity of single stranded probe, DNA target A/T and RNA target A/U - fluorescence emission intensity of probe-target duplex, AU - arbitrary units, number above bar = F_{ds}/F_{ss}. Detailed values: see Table S8 and S10.

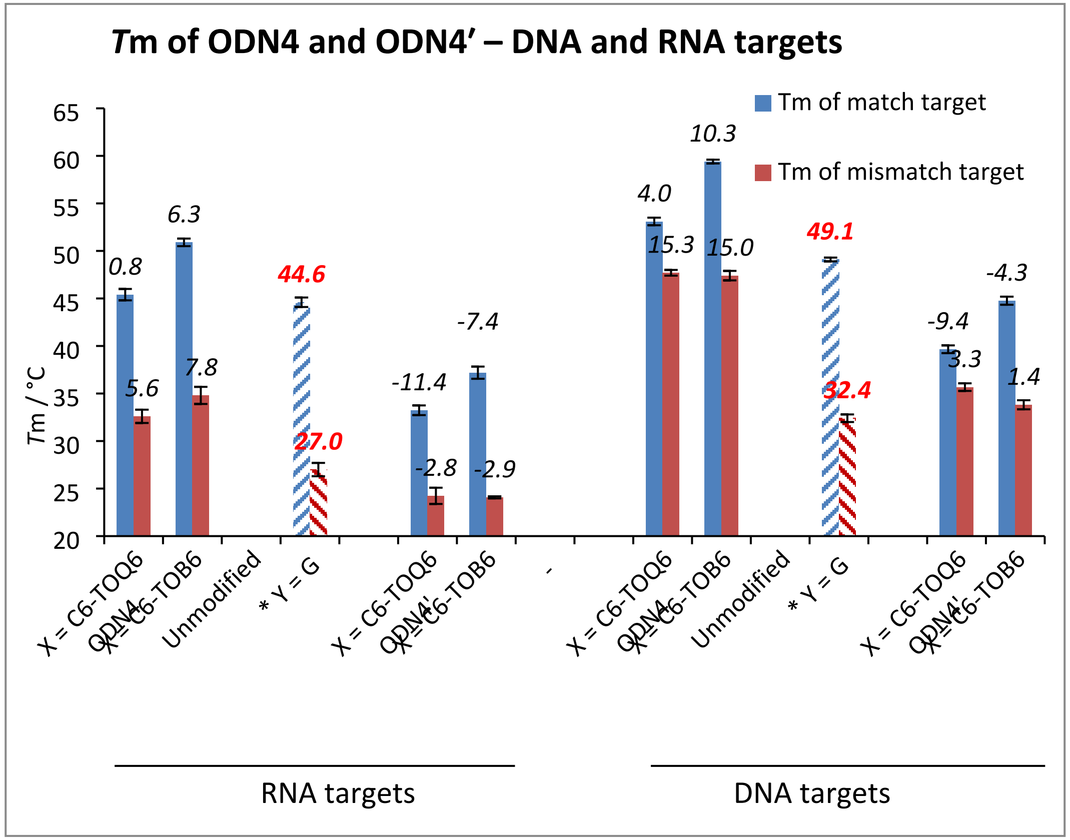

Figure S20 UV melting data for ODN4 (5′-GCAG**X**GTACG) and ODN4′ (5′-GCAI**X**ITACG) with $TO_{B/Q6}$ probes against DNA and RNA targets. The base opposite the TO is either A – match or T/U – mismatch, number above bar = ΔT_m to unmodified target, * = T_m value of unmodified matched and mismatched duplexes. Detailed values: see Table S3 and S5.

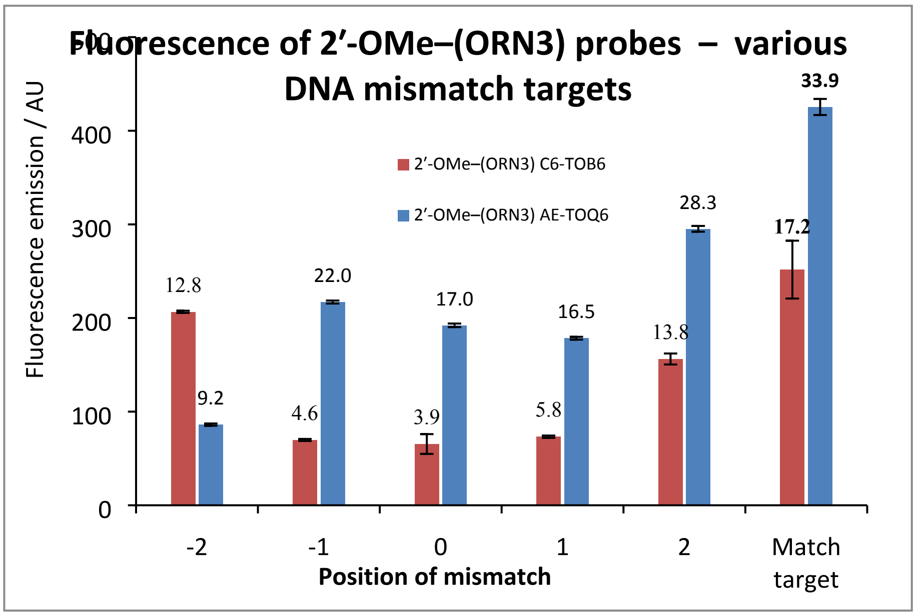

Figure S21 Fluorescence emssion intensity at $\lambda_{em,\ max}$ of TO-modified 2′-OMe–(ORN3) (5′-GCACXCUACG) probes against various DNA targets with mismatches. Position of mismatch refers to a mismatch in the target strand counting from the TO-modified base, e.g.: position "0" is directly opposite modification and "-2" is two nucleobases towards 5′ end of the probe sequence. Value above the bar represents F_{ds}/F_{ss} – ratio of integrated fluorescence emission of oligonucleotide probe-target duplexes to single stranded probes. Detailed values: see Table S9 and S12.

Examples of fluorescence and UV melting curves

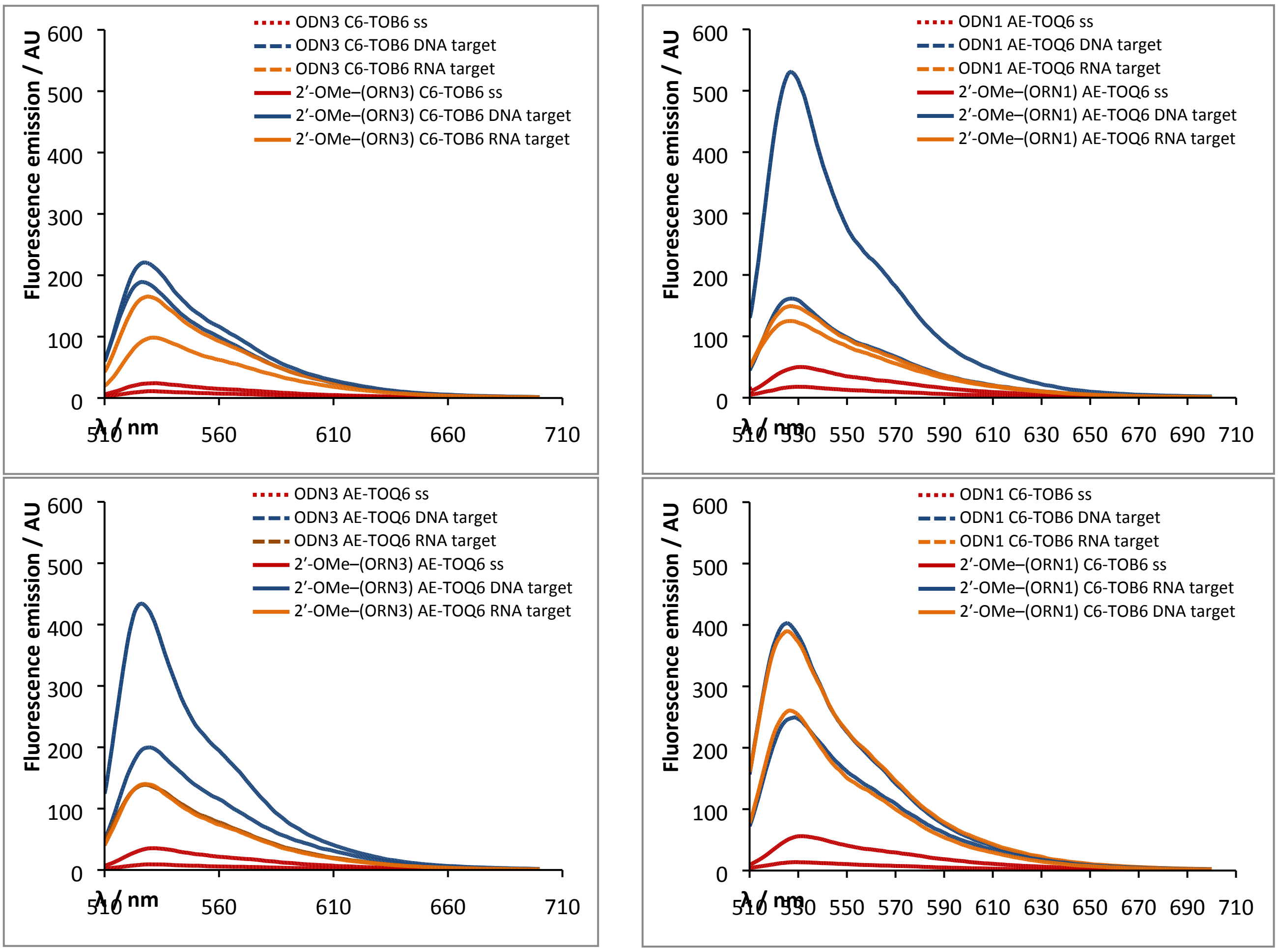

Figure S22 Examples of fluorescence emission spectra of ODN1 (5′GCAT**X**TTACG), 2′-OMe–(ORN1) (5′-GCAU**X**UUACG), ODN3 (5′-GCAC**X**CTACG) and 2′-OMe–(ORN3) (5′-GCAC**X**CUACG)) with C6-TO_{B6} and AE-TO_{Q6} probes with DNA or RNA targets. λ_{ex} = 484 nm, 200mM NaCl, 10 mM phosphate, pH = 7.0 at 20 °C.

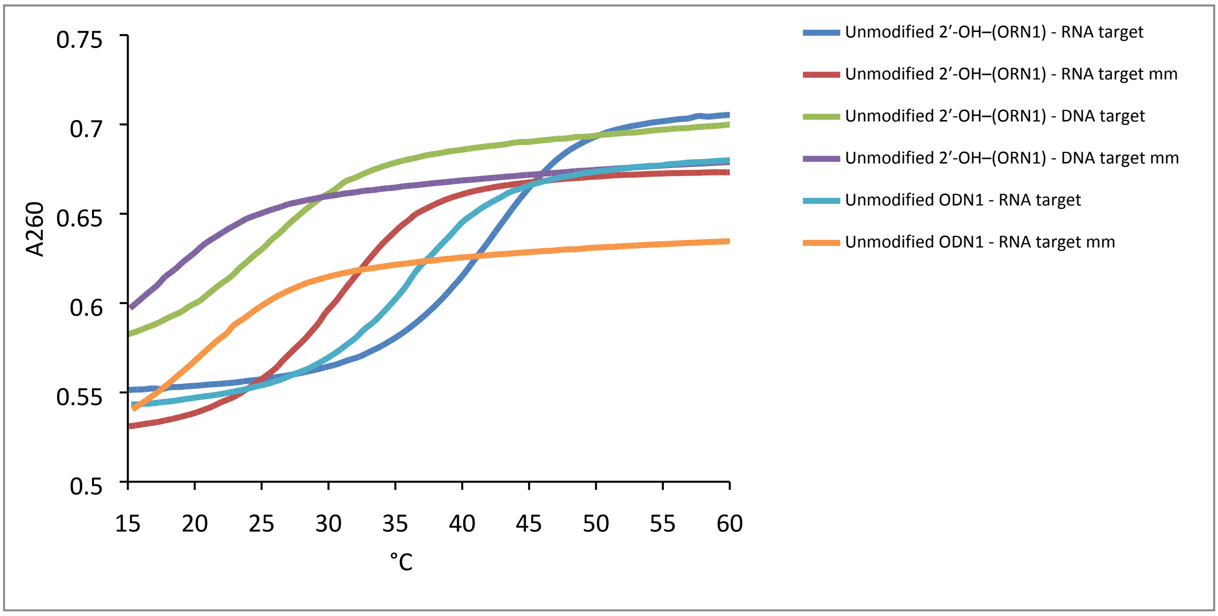

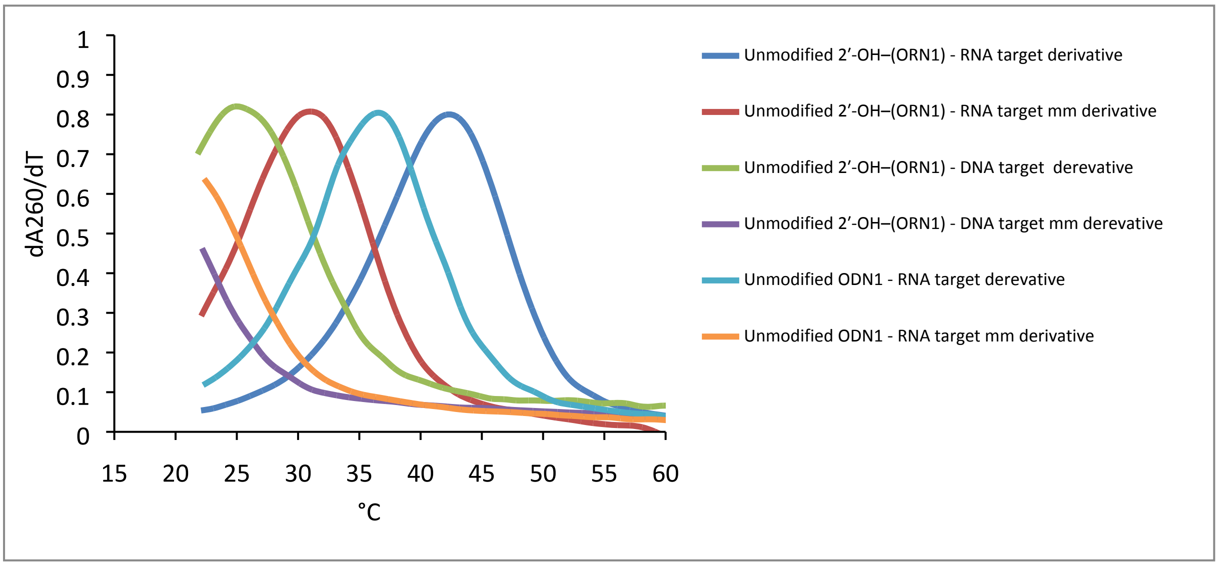

Figure S23 Examples of UV melting curves (top panel) and normalized derivatives (bottom panel) for unmodified 2′-OH–(ORN1) (5′-GCAU**U**UUACG) and ODN1 (5′-GCAT**T**TTACG) unmodified controls against match and mismatch (mm) DNA and RNA targets.

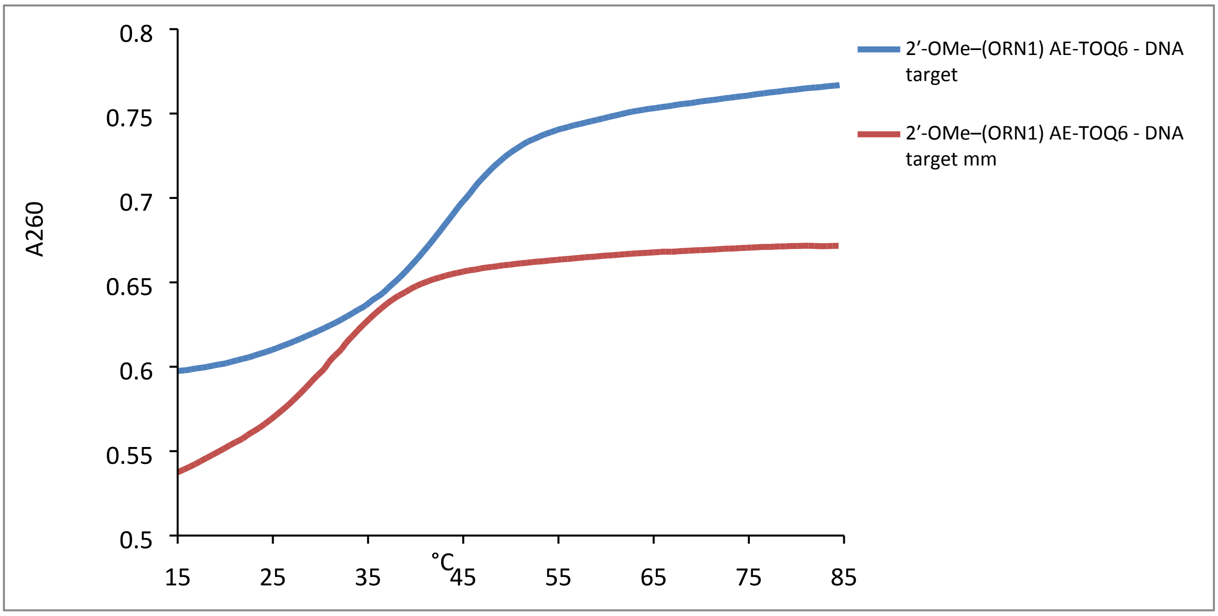

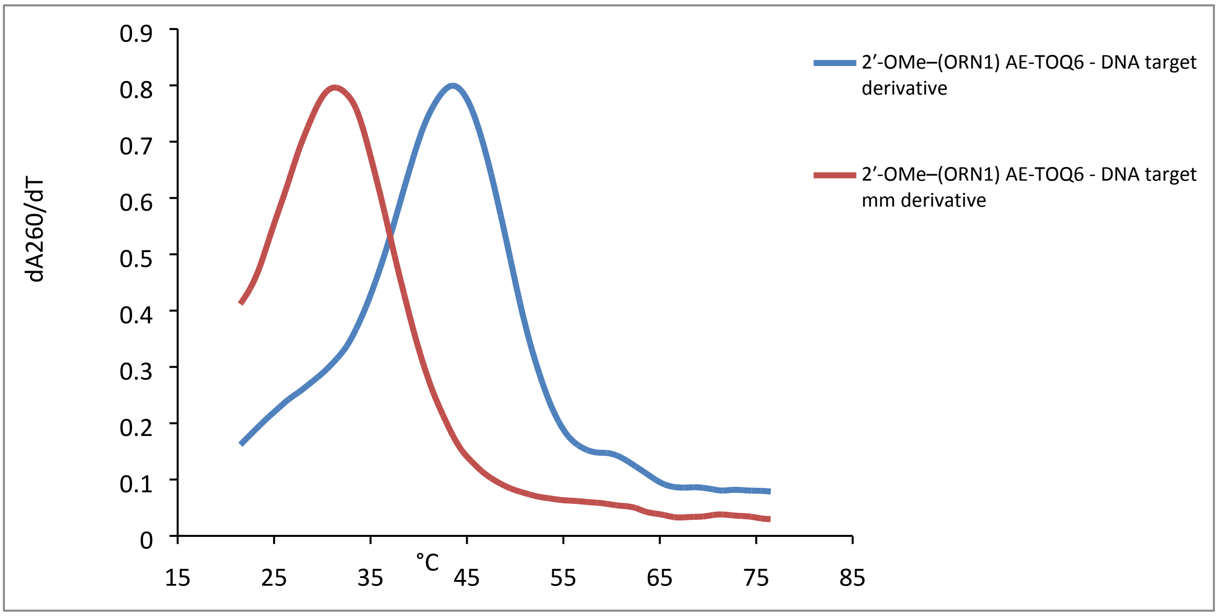

Figure S24 Examples of UV melting curves (top panel) and normalized derivatives (bottom panel) for 2′-OMe–(ORN1) (5′-GCAA**X**AUACG) probes with AE-TO_{Q6} against match and mismatch (mm) DNA targets.

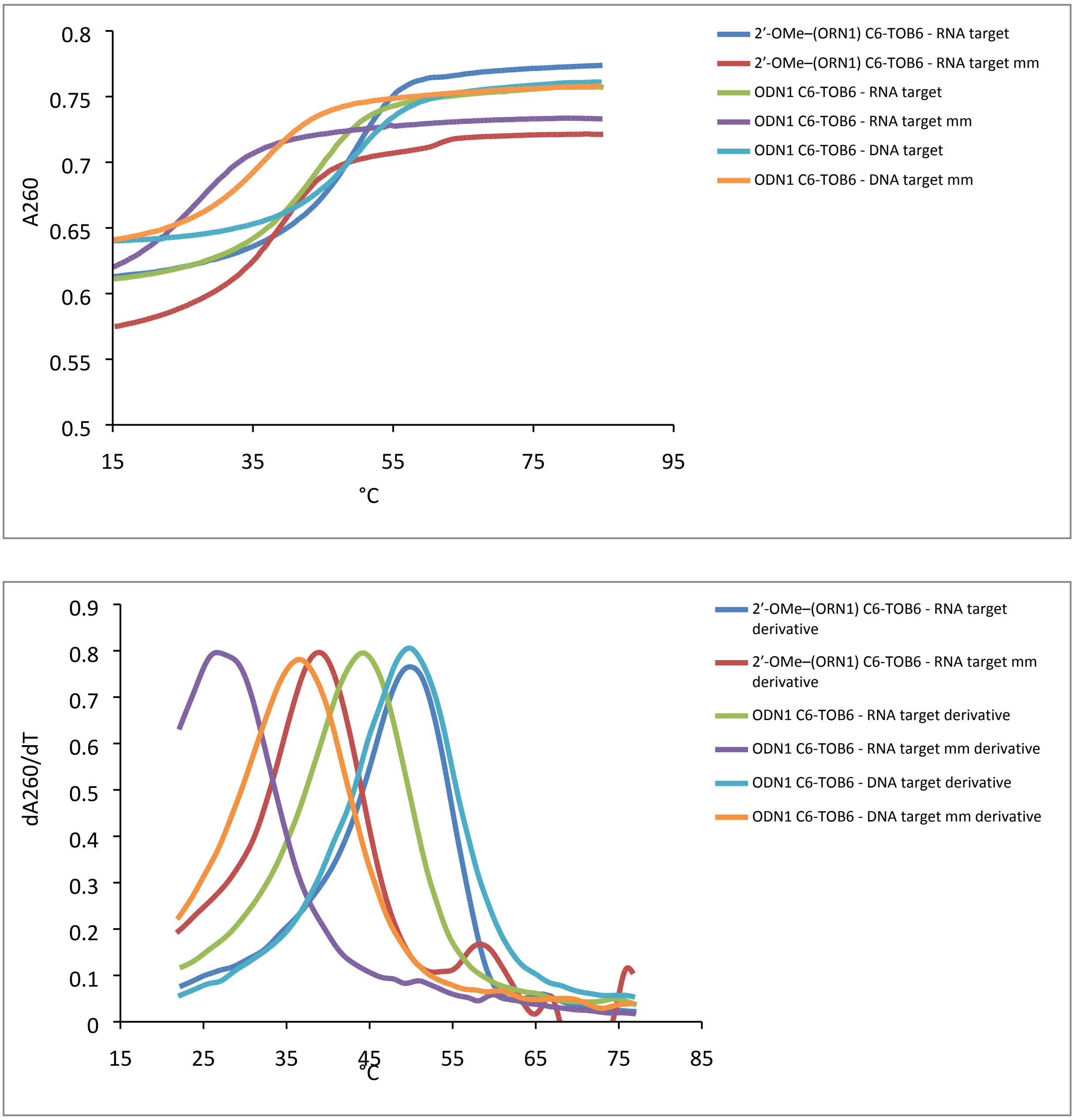

Figure S25 Examples of UV melting curves (top panel) and normalized derivatives (bottom panel) for 2′-OMe–(ORN1) (5′-GCAUXUUACG) and ODN1 (5′-GCATXTTACG) probes with C6-TO_{B6} against match and mismatch (mm) DNA and RNA targets.

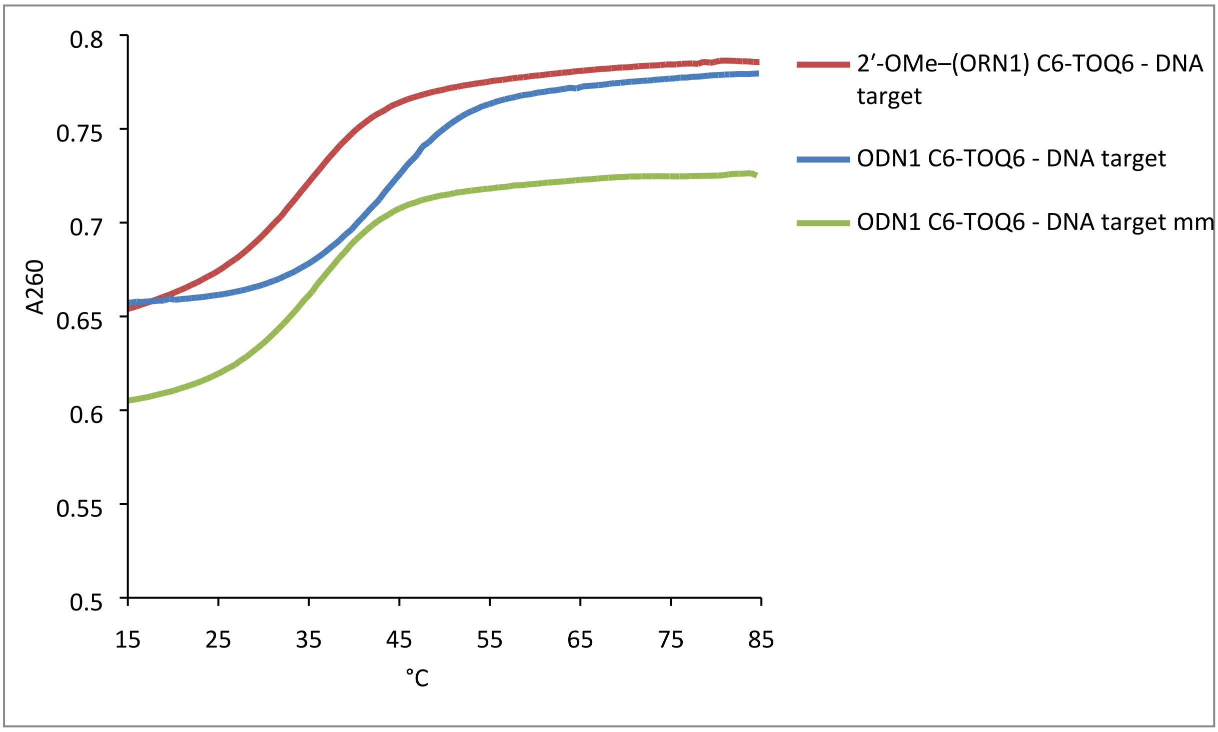

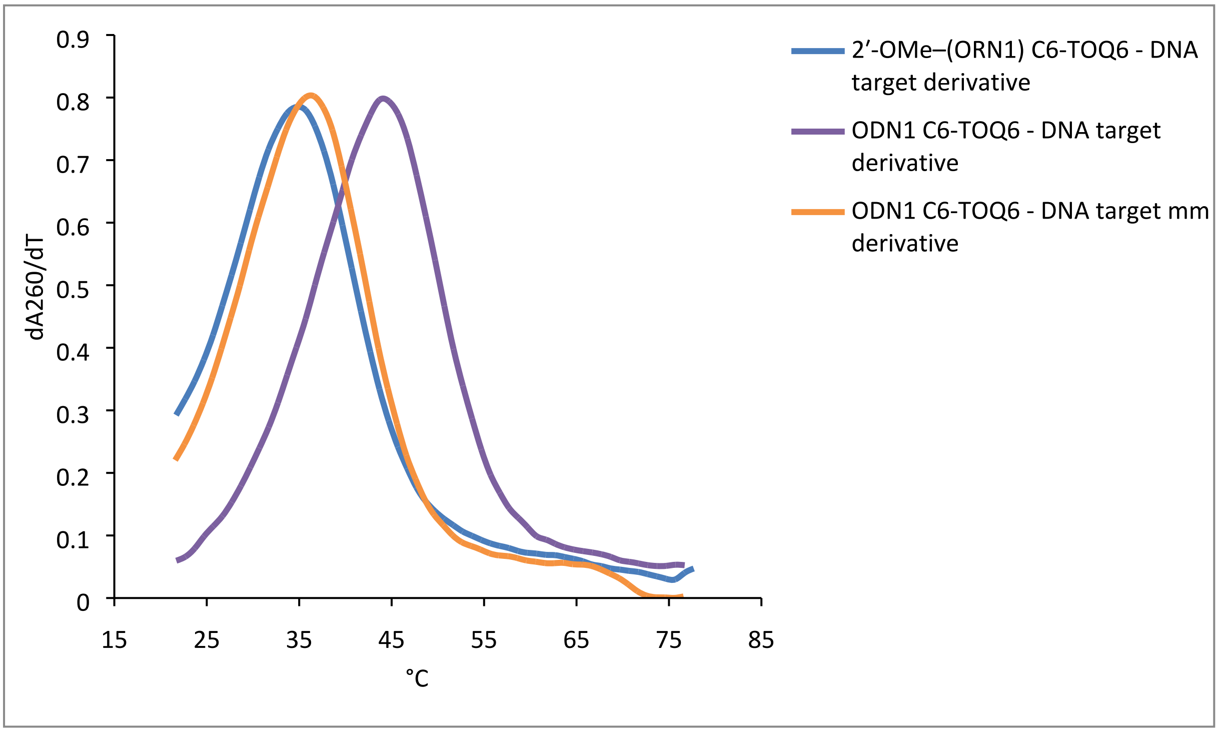

Figure S26 Examples of UV melting curves (top panel) and normalized derivatives (bottom panel) for 2′-OMe–(ORN1) 5′-GCAUXUUACG and ODN1 (5′-GCATXTTACG) probes with C6-TO$_{Q6}$ against match and mismatch (mm) DNA targets.

CD spectra

A

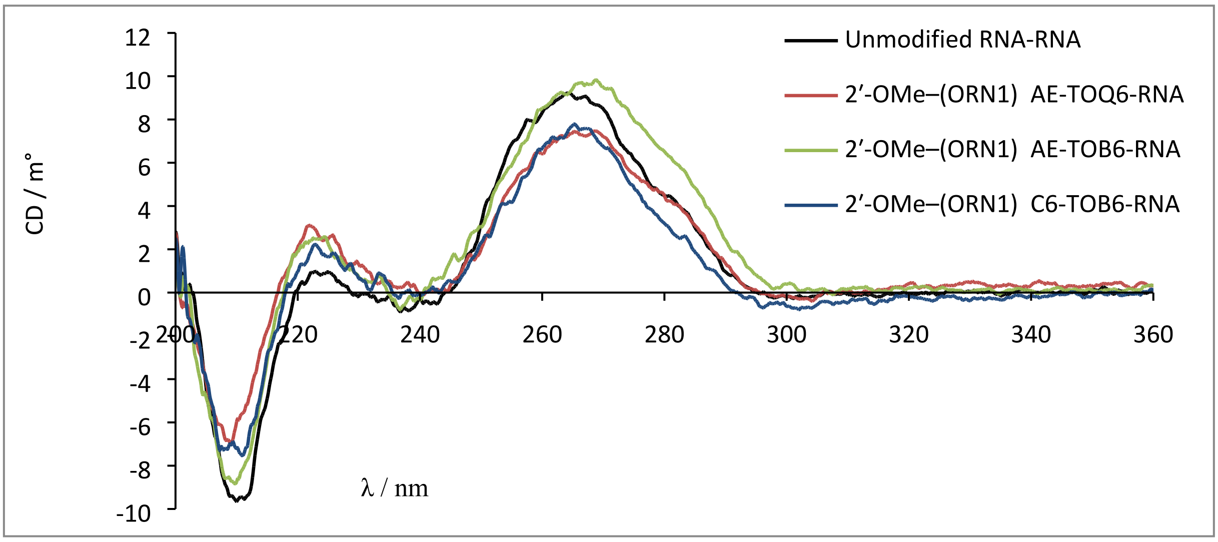

B

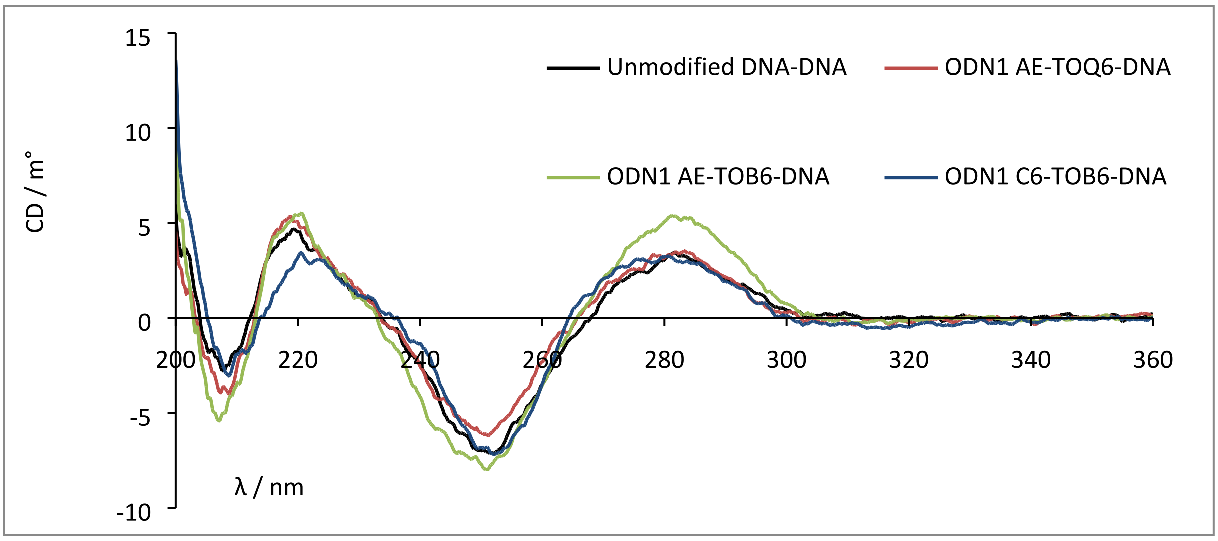

Figure S27 CD spectra of selected TO-labelled probes-DNA/RNA targets duplexes and unmodified DNA/RNA-DNA/RNA target duplexes. A – 2'-OMe–(ORN1) (5'-GCAU**X**UUACG) probes and unmodified RNA 2'-OH–(5'-GCAU**U**UUACG) with RNA target, B – ODN1 (5'-GCAT**X**TTACG) probes and unmodified DNA (5'-GCAT**T**TTACG) with DNA target.

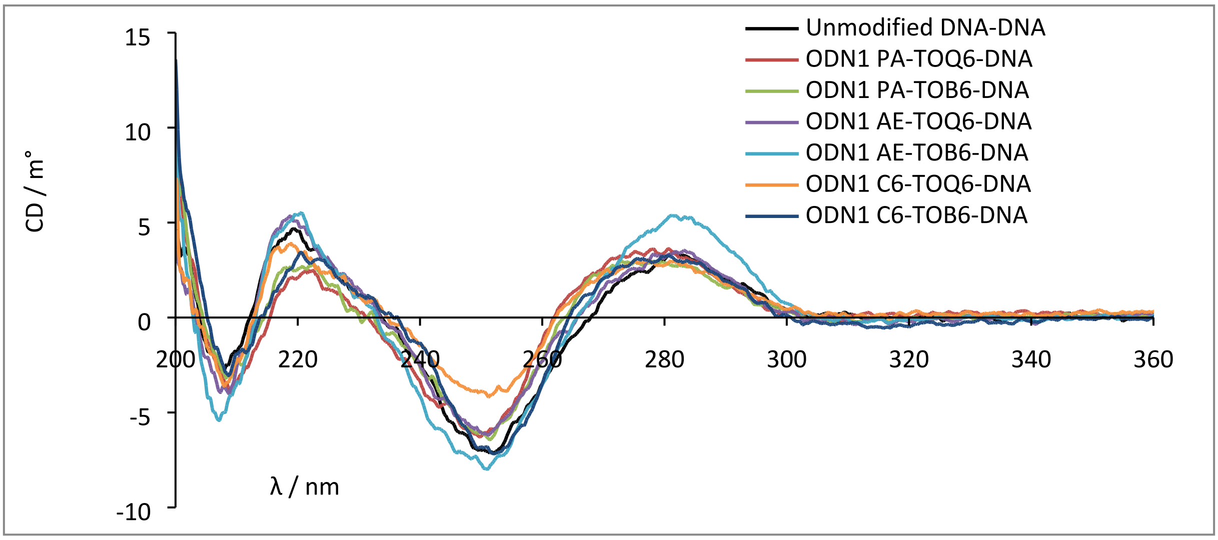

Figure S28 CD spectra of TO-labelled ODN1 (5′-GCAT**X**TTACG) probes-DNA target duplexes with comparison to unmodified DNA (5′-GCAT**T**TTACG) duplex with DNA target.

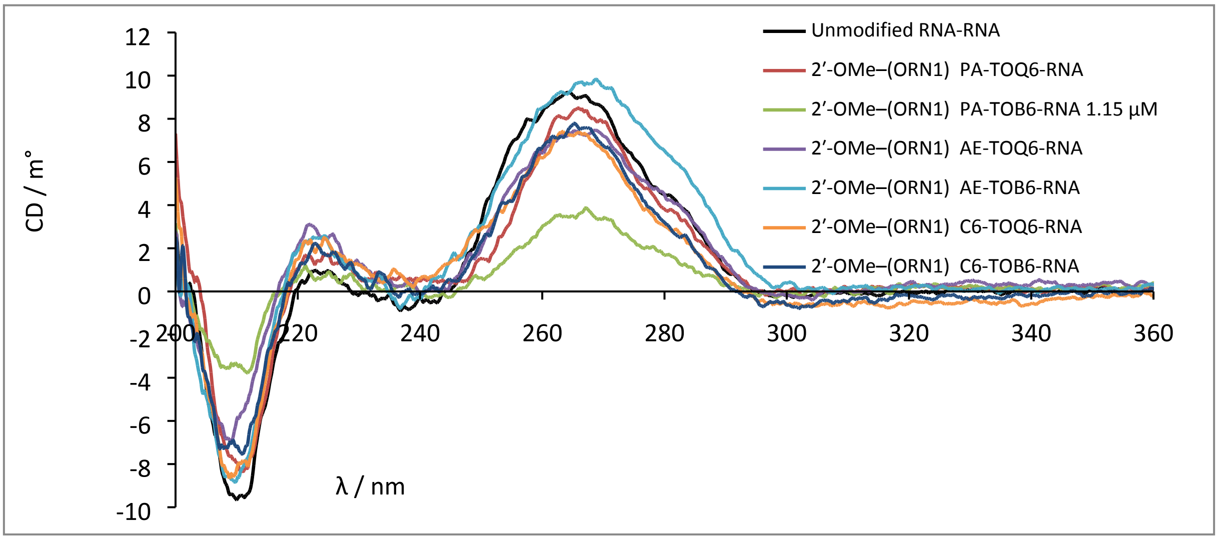

Figure S29 CD spectra of TO-labelled 2′-OMe–(ORN1) (5′-GCAU**X**UUACG) probes-RNA target duplexes with comparison to unmodified RNA 2′OH–(5′-GCU**U**UUACG) duplex with RNA target.

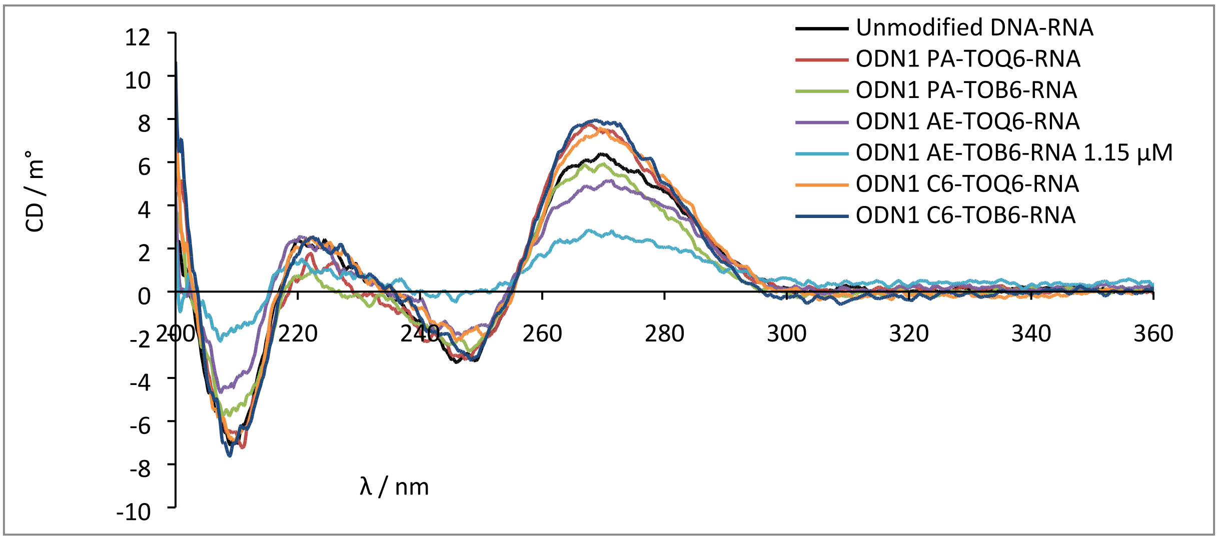

Figure S30 CD spectra of TO-labelled ODN1 (5′-GCAT**X**TTACG) probes-RNA target duplexes with comparison to unmodified DNA (5′-GCT**T**TTACG) duplexe with RNA target.

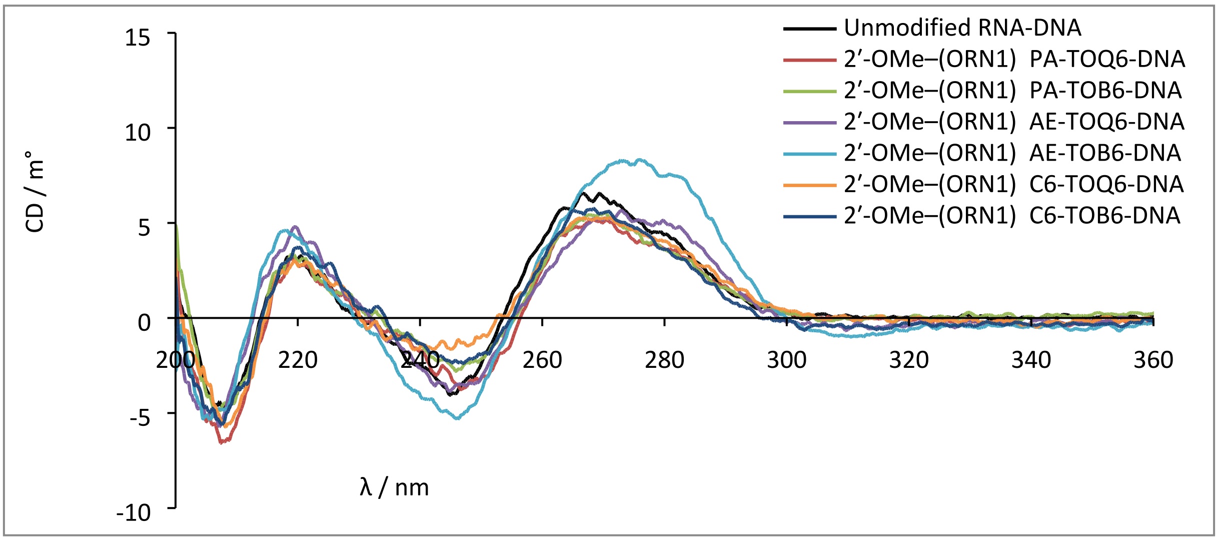

Figure S31 CD spectra of TO-labelled 2′-OMe–(ORN1) (5′-GCAU**X**UUACG) probes-DNA target duplexes with comparison to unmodified RNA 2′OH–(5′-GCU**U**UUACG) duplex with DNA target.

NMR spectra

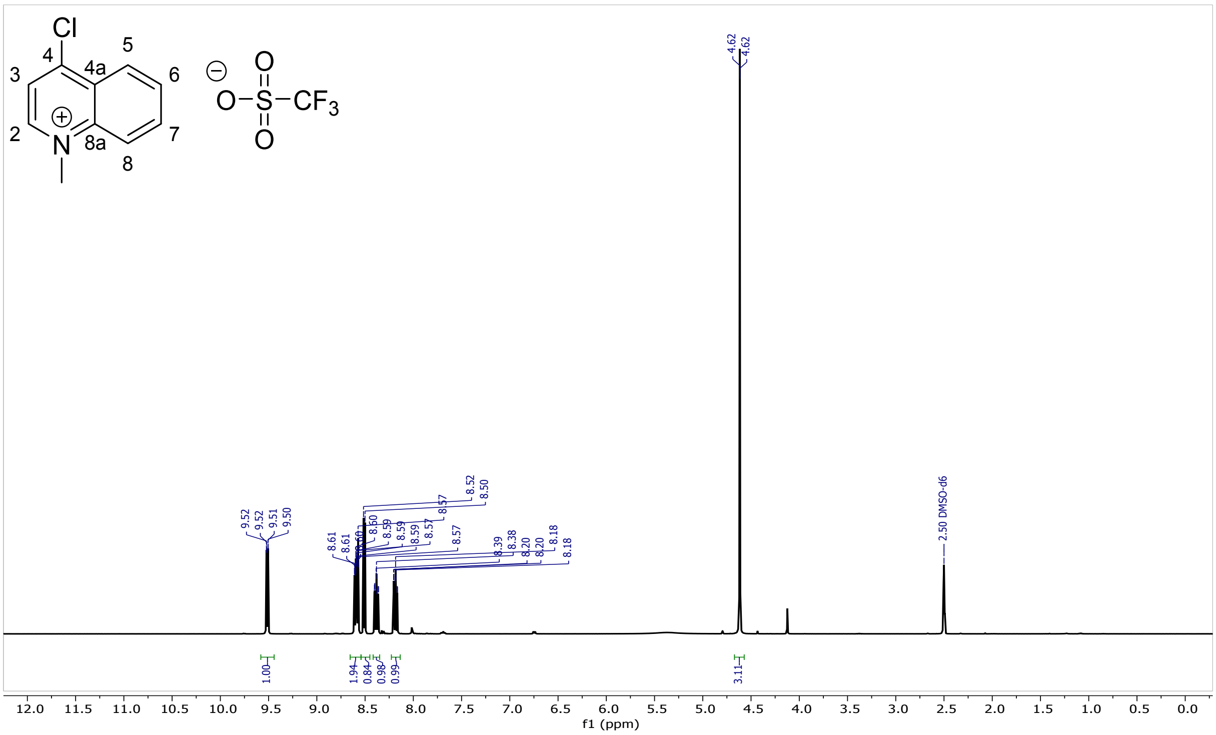

Figure S32 ^{1}H NMR d_6-DMSO spectrum of **2** at 400 MHz

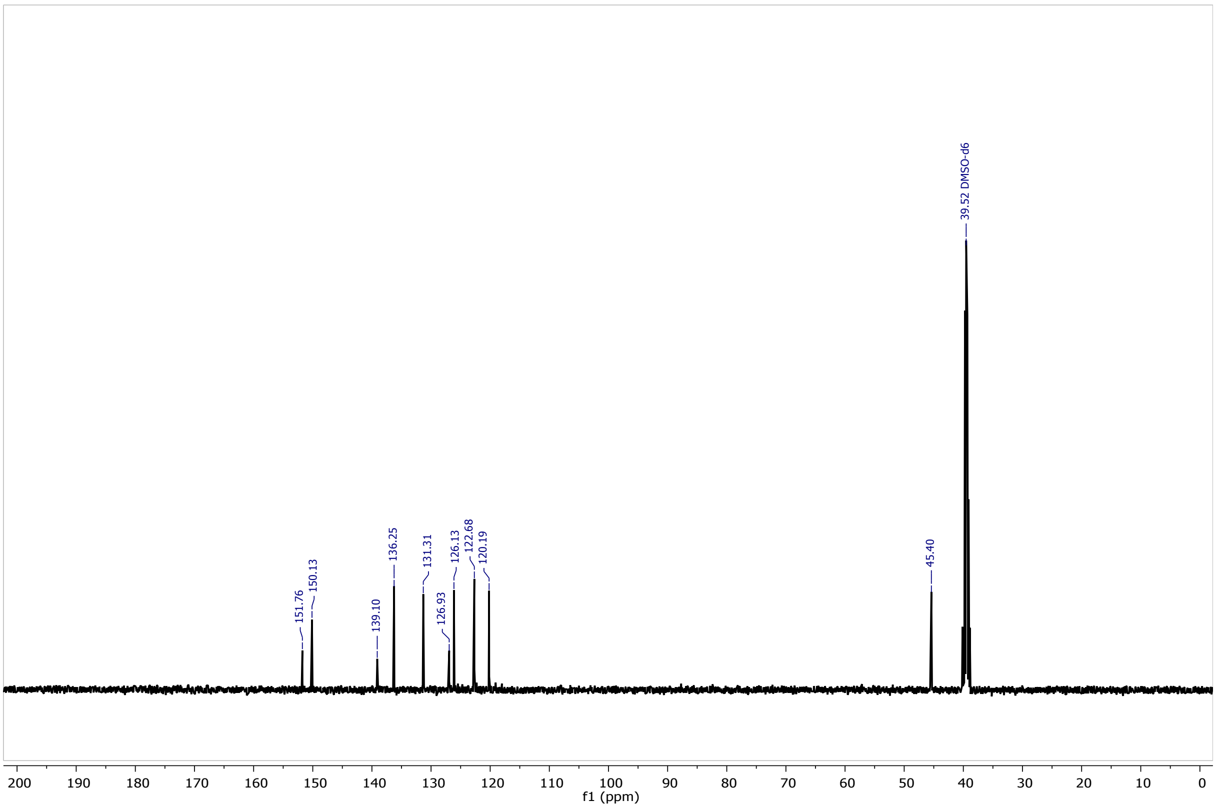

Figure S33 ^{13}C NMR d_6-DMSO spectrum of **2** at 101 MHz

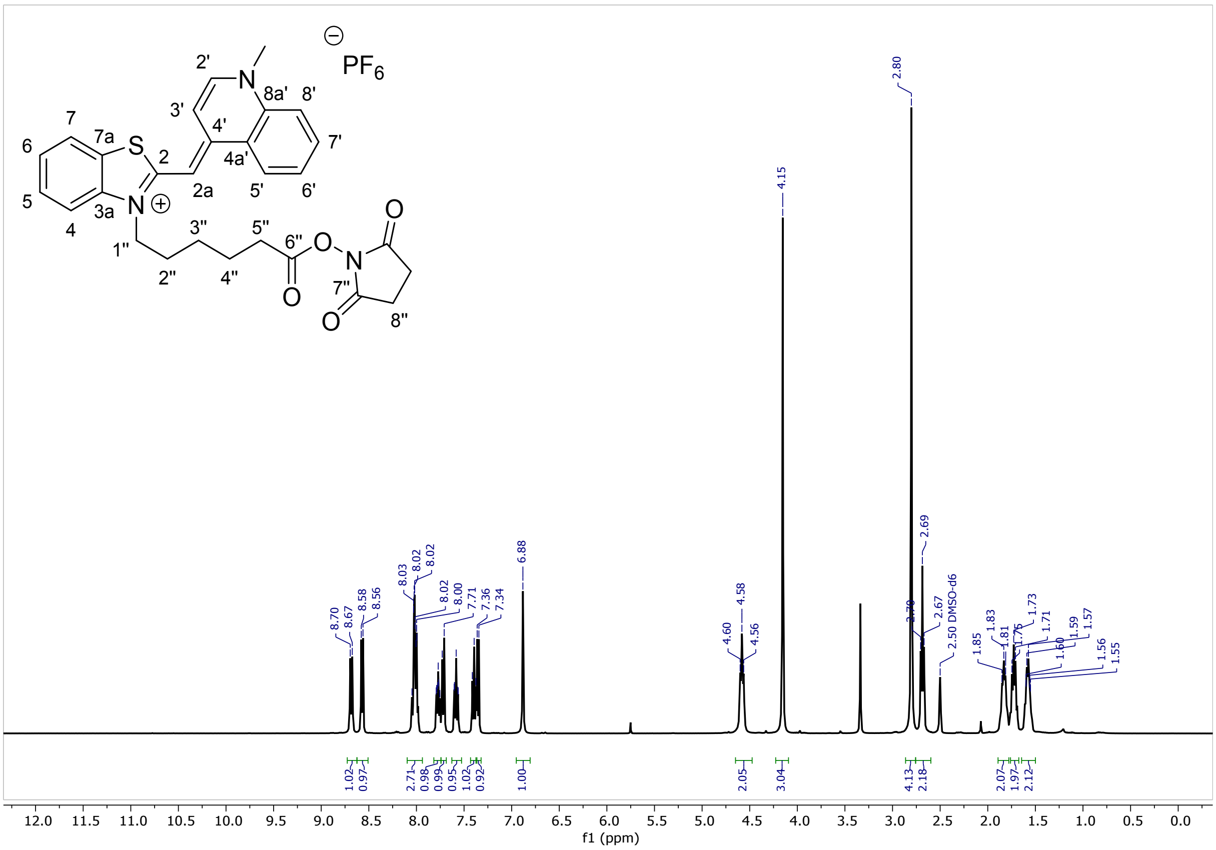

Figure S34 ^{1}H NMR d_6-DMSO spectrum of **4** at 400 MHz

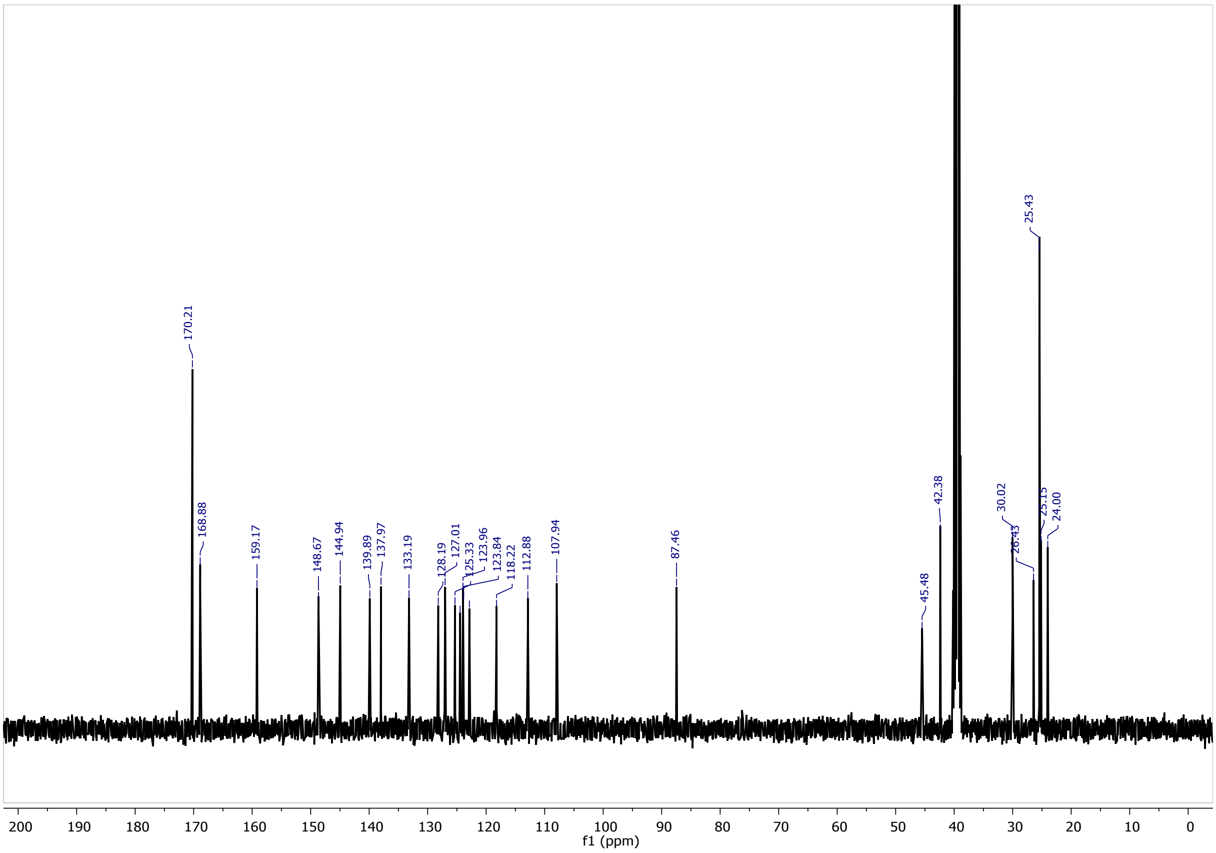

Figure S35 ^{13}C NMR d_6-DMSO spectrum of **4** at 101 MHz

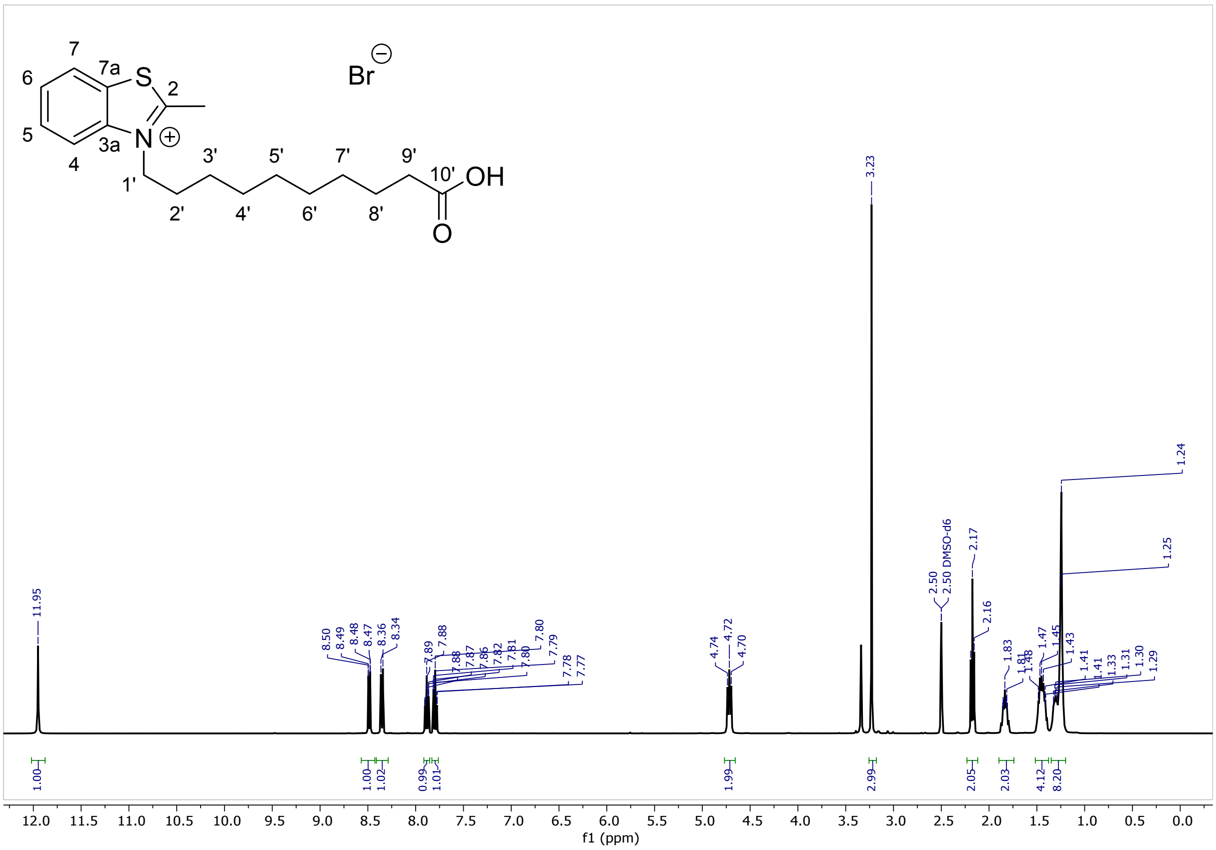

Figure S36 ^{1}H NMR d_6-DMSO spectrum of **9** at 400 MHz

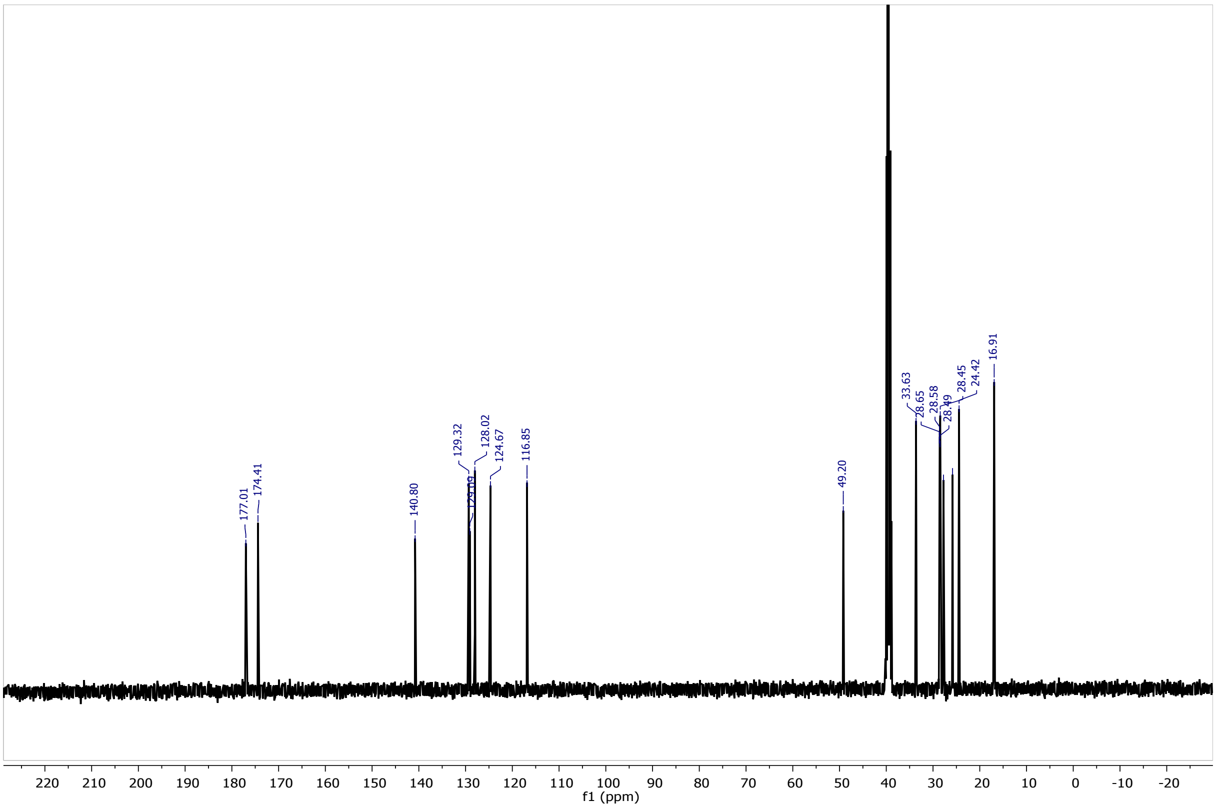

Figure S37 ^{13}C NMR d_6-DMSO spectrum of **9** at 101 MHz

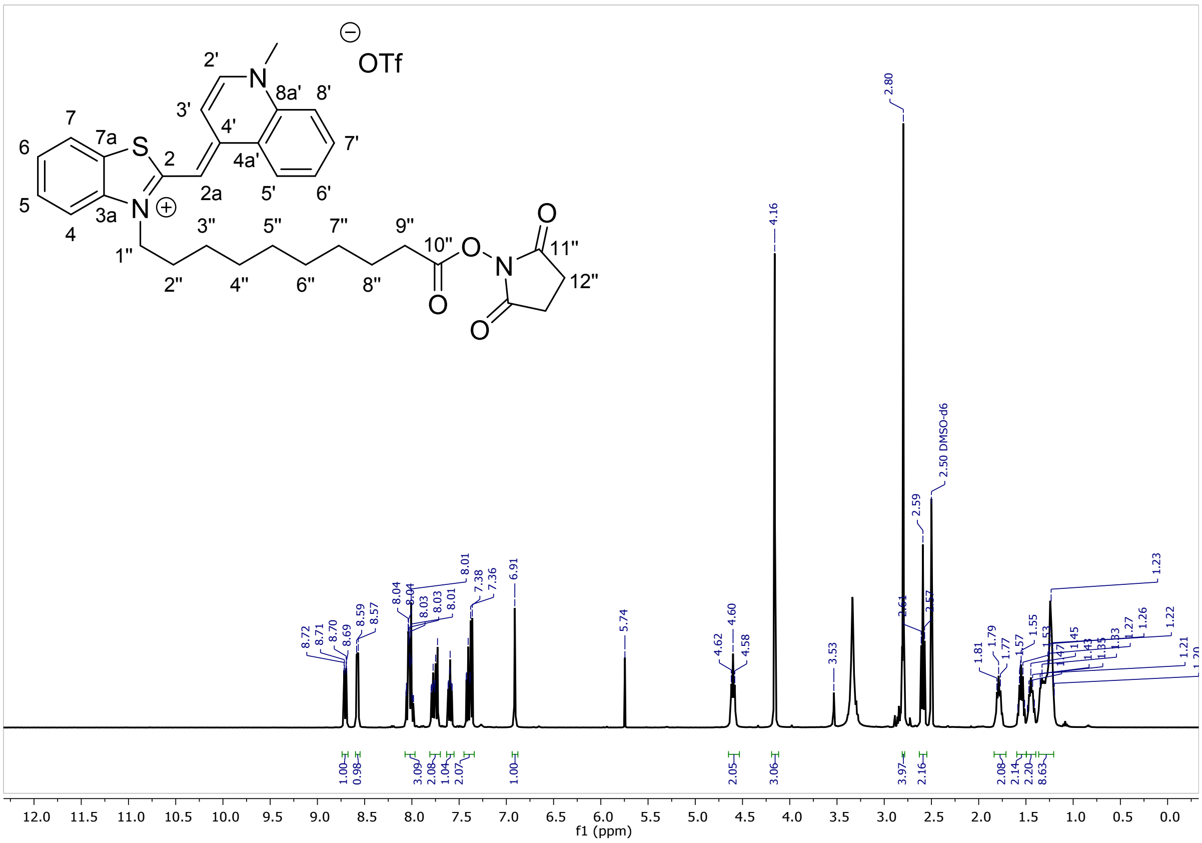

Figure S38 ^{1}H NMR d_6-DMSO spectrum of **11** at 400 MHz

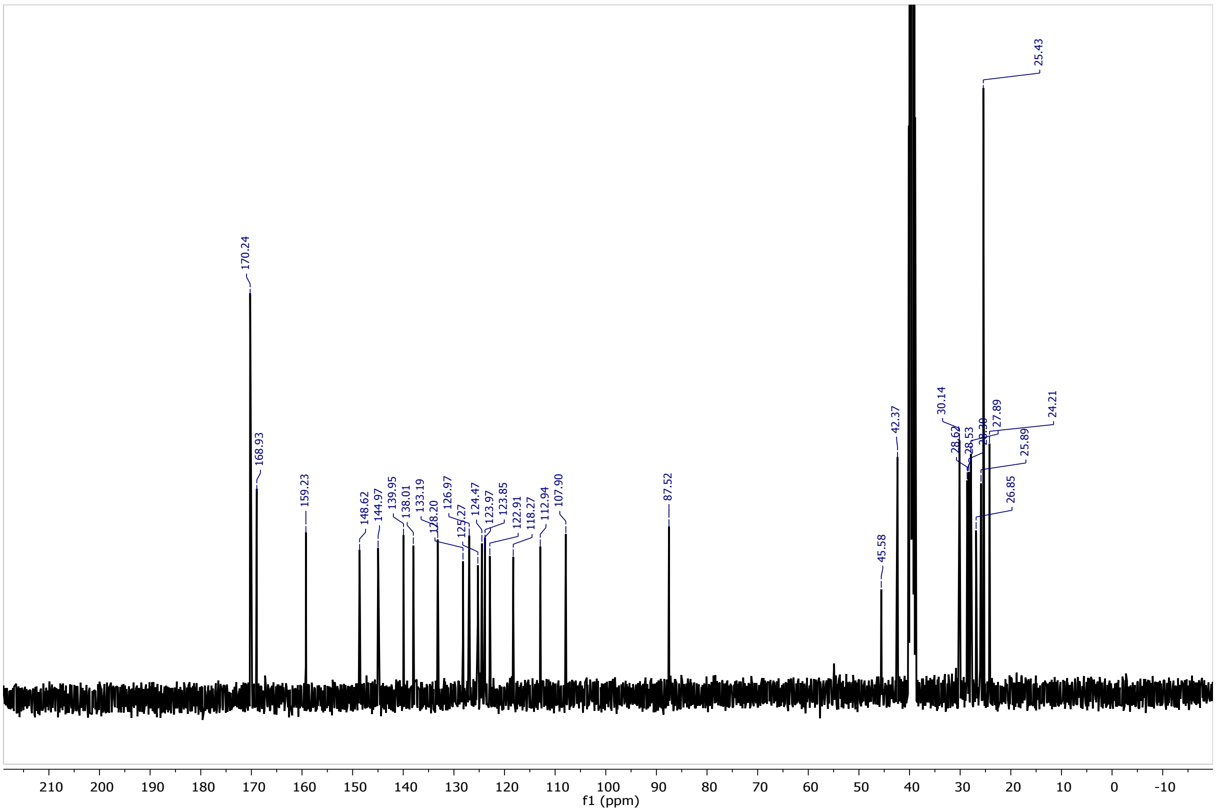

Figure S39 ^{13}C NMR d_6-DMSO spectrum of **11** at 101 MHz

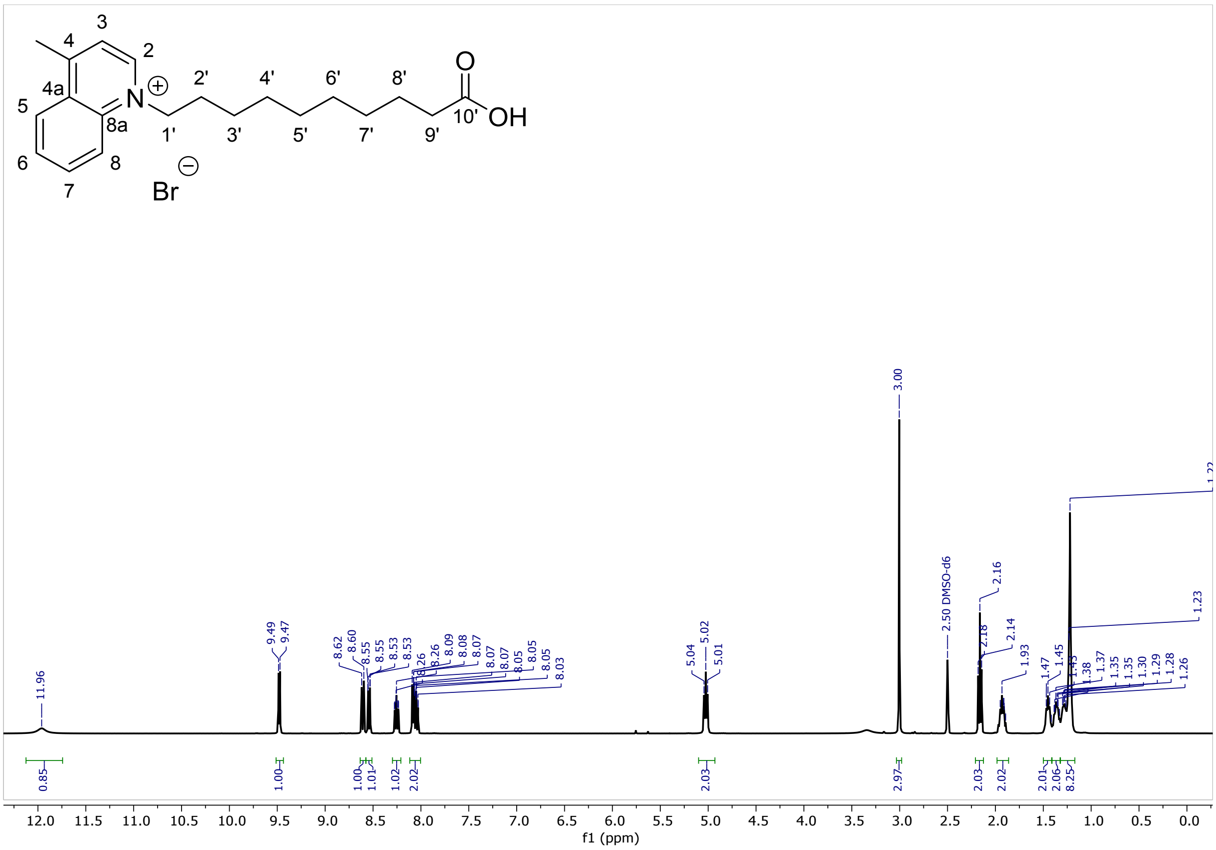

Figure S40 ^{1}H NMR d_6-DMSO spectrum of **12** at 400 MHz

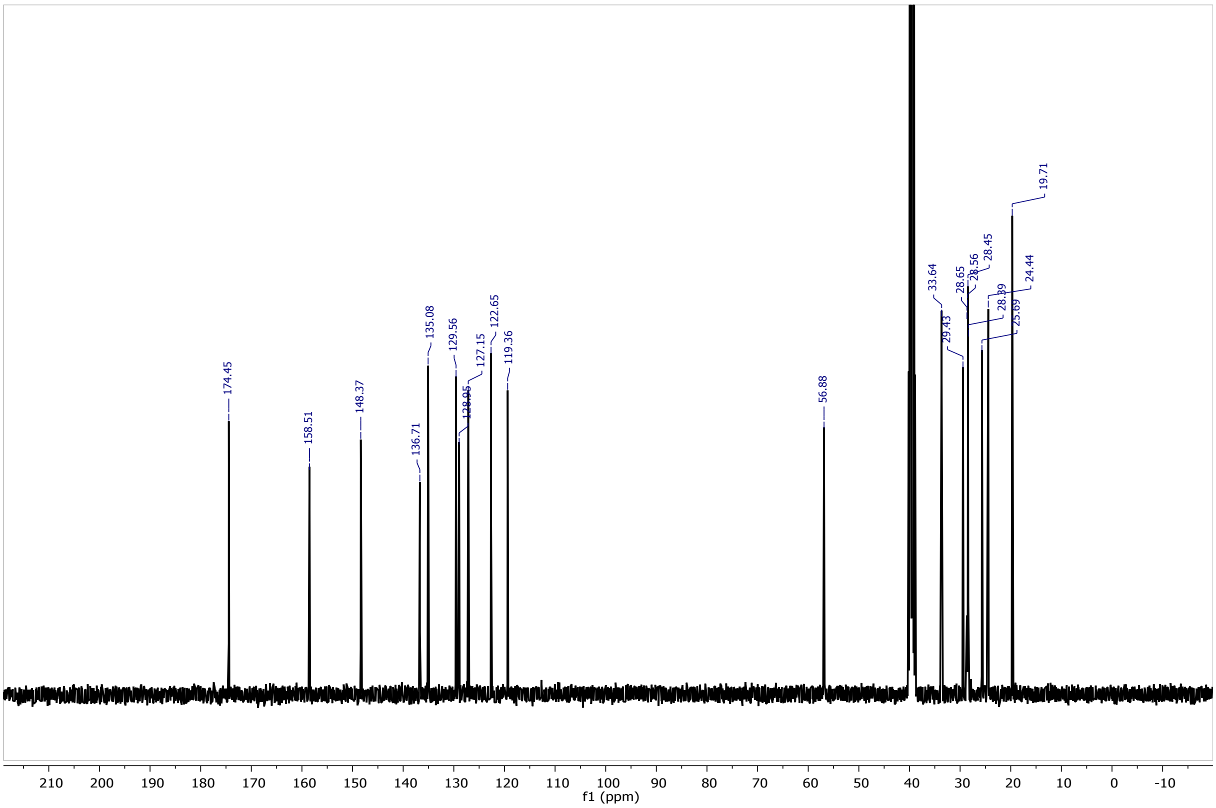

Figure S41 ^{13}C NMR d_6-DMSO spectrum of **12** at 101 MHz

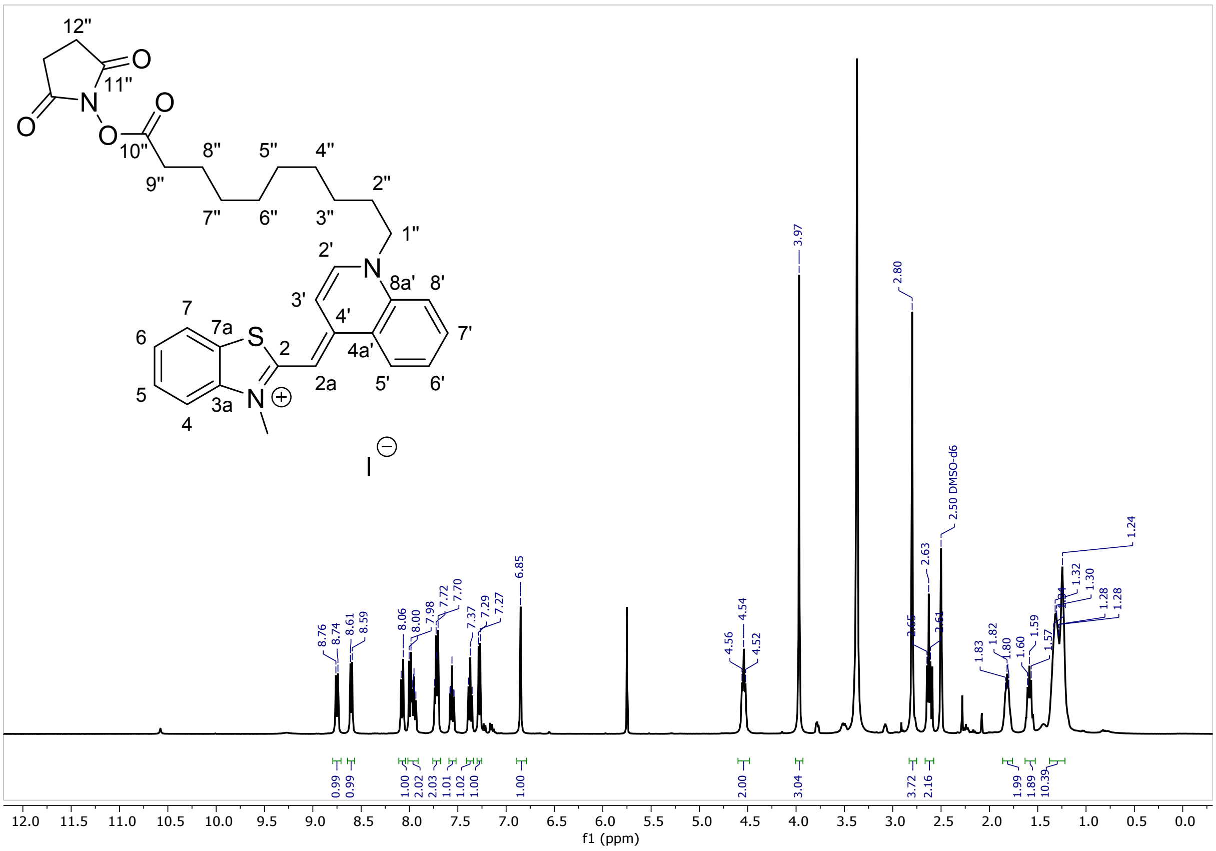

Figure S42 ^{1}H NMR d_6-DMSO spectrum of **14** at 400 MHz

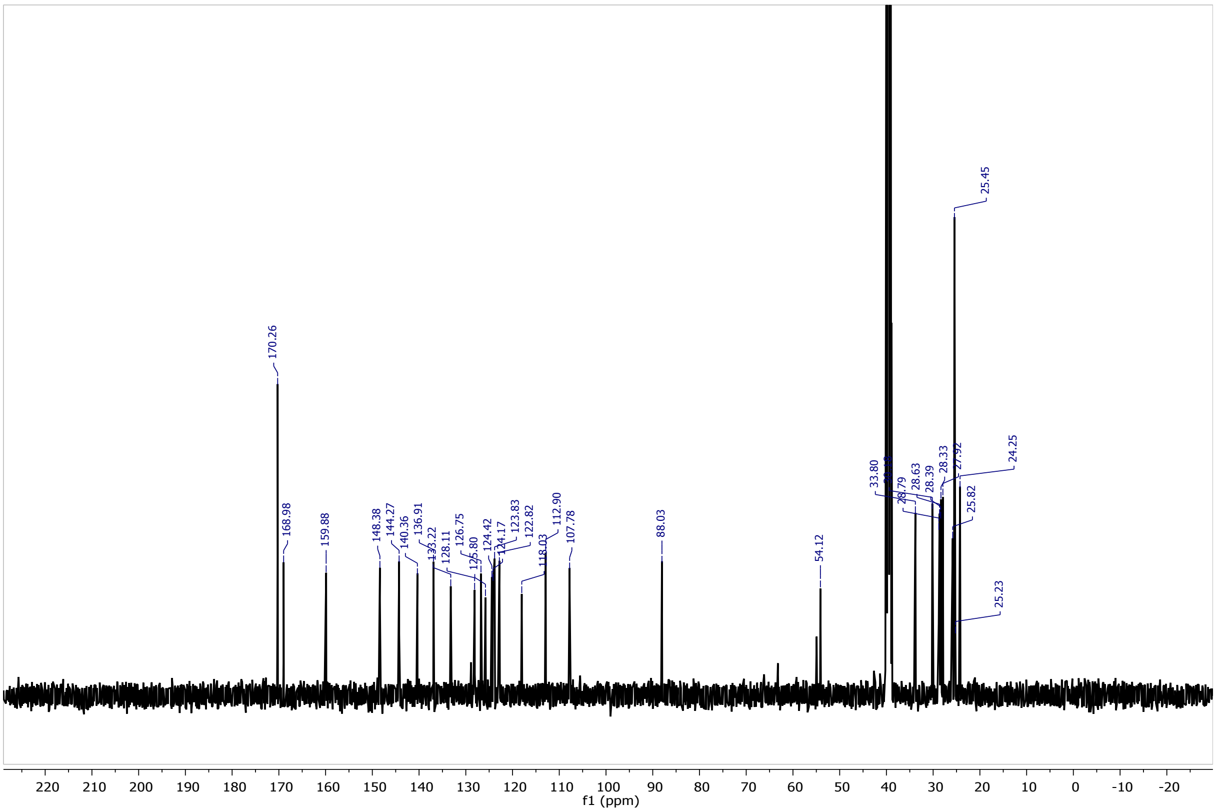

Figure S43 ^{13}C NMR d_6-DMSO spectrum of **14** at 101 MHz

Table S13 Literature on attachment of TO to oligonucleotides and PNAs.

Oligonucleotide based	Ref.
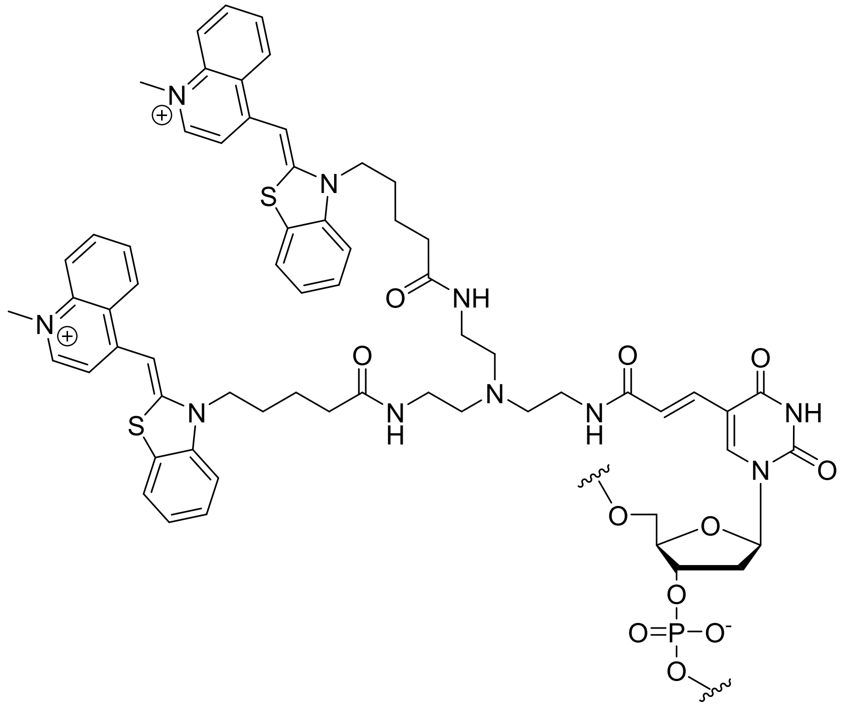	10
Used with DNA backbone	10
Used with 2'-OMe RNA backbone	11
Used with LNA backbone	12
Used with cytidine as a base	13
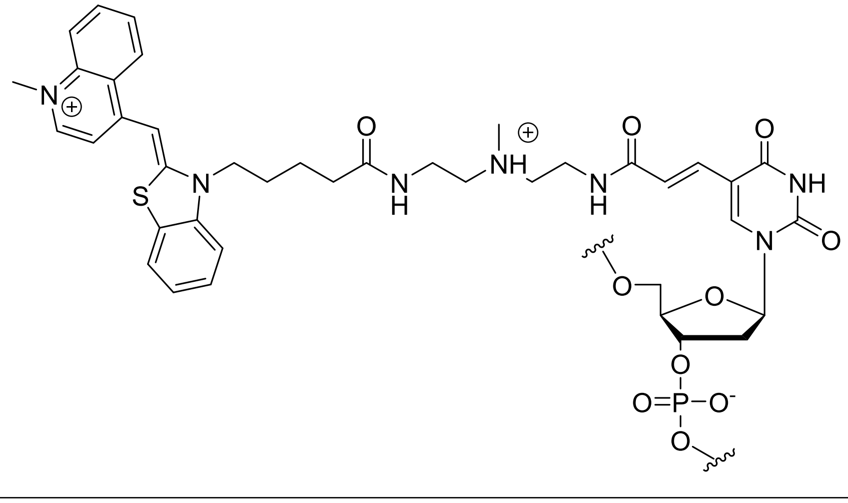	10

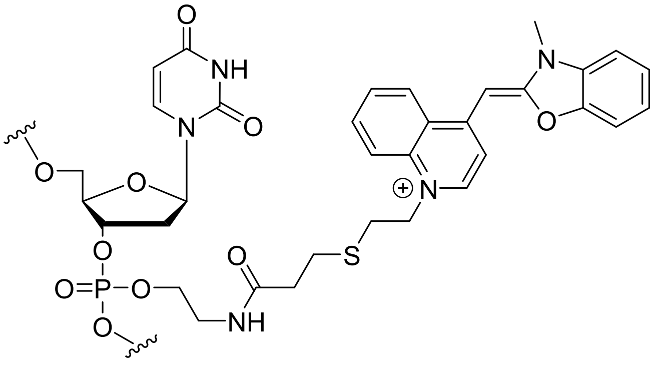	14
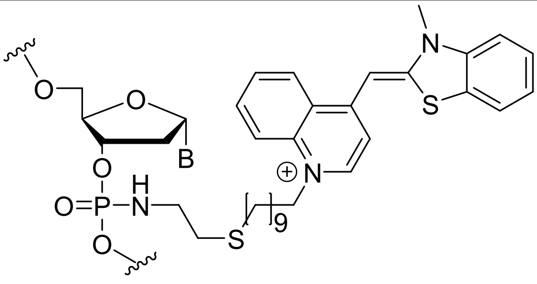	15
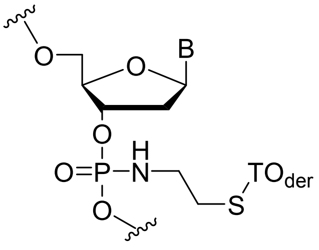	16
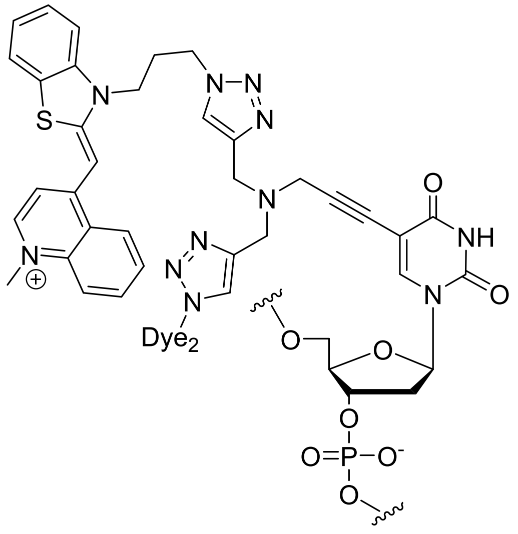	17
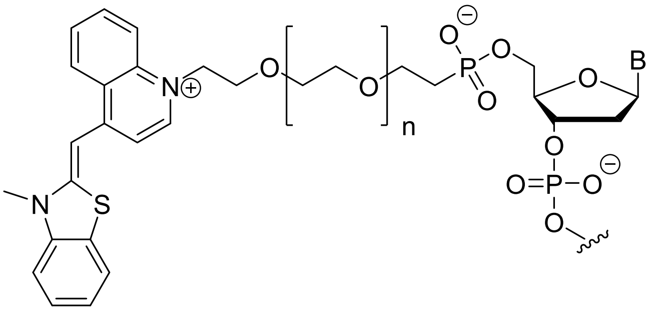	18

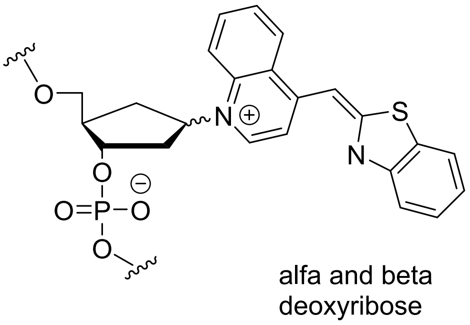	19
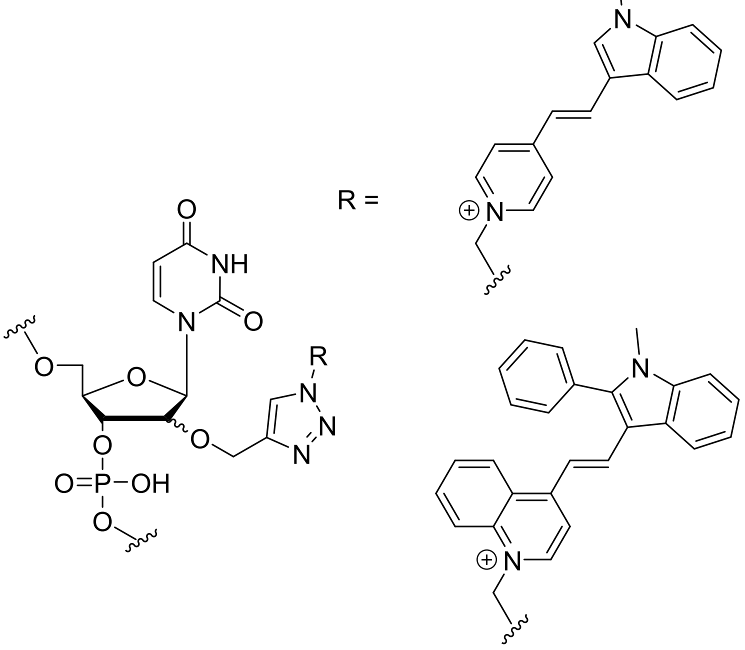	20
PNA based	
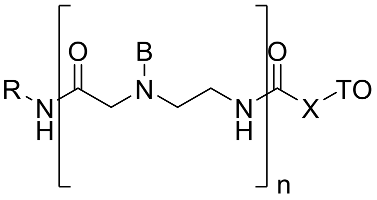 $X = (CH_2)_y–NH–CO–(CH_2)_y$<br>y = 5 or 10<br>TO = TO_B or TO_Q	21
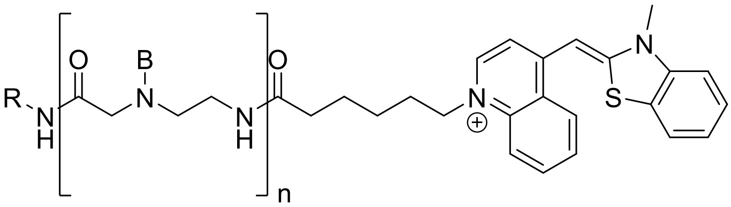	22

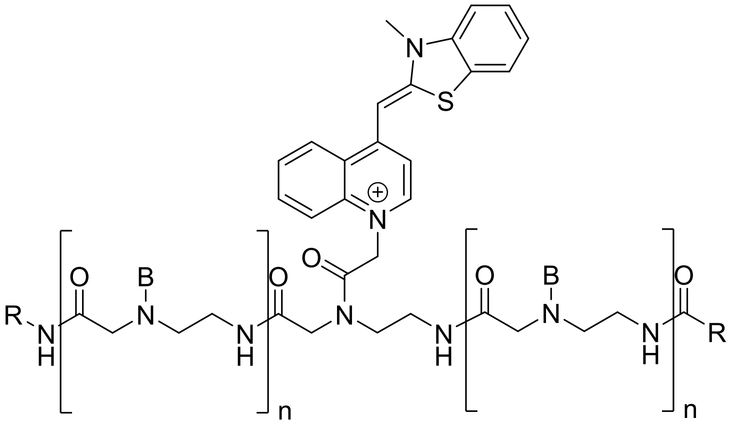	23, 24
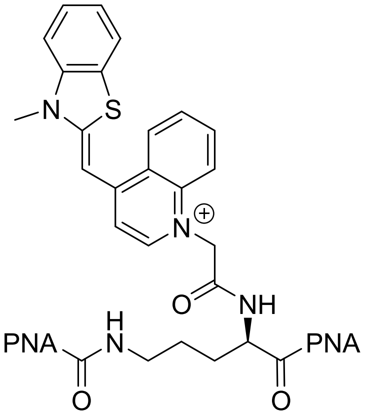 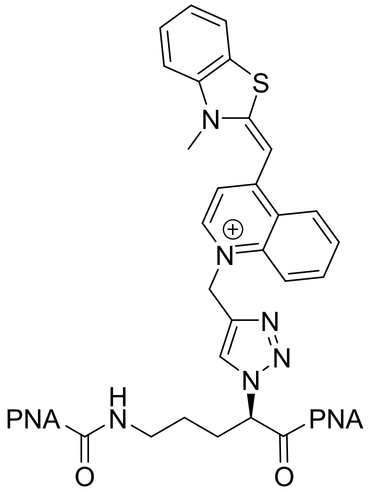	25
Backbone modification	
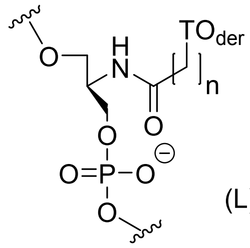 (L) and (D) Serinol	26
With LNA	27
With 2’OMe RNA backbone	27
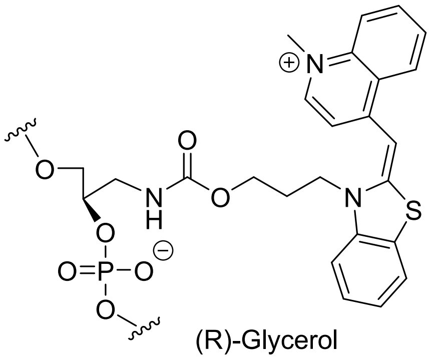 (R)-Glycerol	28, 29

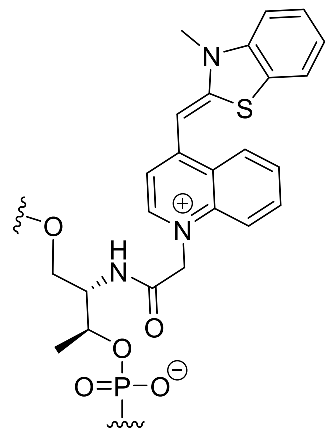	30

References

1. D. S. Pisoni, L. Todeschini, A. C. A. Borges, C. L. Petzhold, F. S. Rodembusch and L. F. Campo, *J. Org. Chem.*, 2014, **79**, 5511-5520.
2. S. Ikeda, T. Kubota, K. Kino and A. Okamoto, *Bioconj. Chem.*, 2008, **19**, 1719-1725.
3. J. R. Carreon, K. P. Mahon and S. O. Kelley, *Org. Lett.*, 2004, **6**, 517-519.
4. X. Fei, R. Li, D. Lin, Y. Gu and L. Yu, *J. Fluorescence*, 2015, **25**, 1251-1258.
5. L. Bethge, D. V. Jarikote and O. Seitz, *Biorg. Med. Chem.*, 2008, **16**, 114-125.
6. T. Sato, Y. Sato and S. Nishizawa, *Chem. Eur. J.*, 2017, **23**, 4079-4088.
7. J. Bijapur, S. Bergqvist, T. Brown, M. D. Keppler and K. R. Fox, *Nucleic Acid Res.*, 1999, **27**, 1802-1809.
8. B. Cuenoud, F. Casset, D. Hüsken, F. Natt, R. M. Wolf, K.-H. Altmann, P. Martin and H. E. Moser, *Angew. Chem. Int. Ed.*, 1998, **37**, 1288-1291.
9. J. A. Richardson, M. Gerowska, M. Shelbourne, D. French and T. Brown, *ChemBioChem*, 2010, **11**, 2530-2533.
10. S. Ikeda and A. Okamoto, *Chem. Asian J.*, 2008, **3**, 958-968.
11. T. Kubota, S. Ikeda, H. Yanagisawa, M. Yuki and A. Okamoto, *Bioconj. Chem.*, 2009, **20**, 1256-1261.
12. K. Sugizaki and A. Okamoto, *Bioconj. Chem.*, 2010, **21**, 2276-2281.
13. S. Ikeda, M. Yuki, H. Yanagisawa and A. Okamoto, *Tetrahedron Lett.*, 2009, **50**, 7191-7195.
14. T. Ishiguro, J. Saitoh, H. Yawata, M. Otsuka and T. Inoue, *Nucleic Acid Res.*, 1996, **24**, 4992-4997.
15. E. Privat, T. Melvin, U. Asseline and P. Vigny, *Photochem. Photobiol.*, 2004, **74**, 532-532.
16. R. Lartia and U. Asseline, *Chem. Eur. J.*, 2006, **12**, 2270-2281.
17. J. Qiu, A. Wilson, A. H. El-Sagheer and T. Brown, *Nucleic Acid Res.*, 2016, **44**, e138.
18. X. Wang and U. J. Krull, *Bioorg. Med. Chem. Lett.*, 2005, **15**, 1725-1729.
19. F. Hövelmann, L. Bethge and O. Seitz, *ChemBioChem*, 2012, **13**, 2072-2081.
20. J. Steinmeyer, H. K. Walter, M. A. Bichelberger, V. Schneider, T. Kubař, F. Rönicke, B. Olshausen, K. Nienhaus, G. U. Nienhaus, U. Schepers, M. Elstner and H. A. Wagenknecht, *Org. Biomol. Chem.*, 2018, **16**, 3726-3731.
21. N. Svanvik, G. Westman, D. Wang and M. Kubista, *Anal. Biochem.*, 2000, **281**, 26-35.
22. A. Tonelli, T. Tedeschi, A. Germini, S. Sforza, R. Corradini, M. C. Medici, C. Chezzi and R. Marchelli, *Molecular BioSystems*, 2011, **7**, 1684-1692.
23. O. Koehler and O. Seitz, *ChemInform*, 2004, **35**, 2938-2939.
24. O. Köhler, D. V. Jarikote and O. Seitz, *ChemBioChem*, 2005, **6**, 69-77.
25. D. Gahtory, M. Murtola, M. M. J. Smulders, T. Wennekes, H. Zuilhof, R. Strömberg and B. Albada, *Org. Biomol. Chem.*, 2017, **15**, 6710-6714.
26. L. Bethge, I. Singh and O. Seitz, *Org. Biomol. Chem.*, 2010, **8**, 2439-2448.
27. F. Hövelmann, I. Gaspar, S. Loibl, E. A. Ermilov, B. Röder, J. Wengel, A. Ephrussi and O. Seitz, *Angew. Chem. Int. Ed.*, 2014, **53**, 11370-11375.
28. S. Berndl and H. A. Wagenknecht, *Angew. Chem. Int. Ed.*, 2009, **48**, 2418-2421.
29. F. Menacher, M. Rubner, S. Berndl and H.-A. Wagenknecht, *J. Org. Chem.*, 2008, **73**, 4263-4266.
30. Y. Hara, T. Fujii, H. Kashida, K. Sekiguchi, X. Liang, K. Niwa, T. Takase, Y. Yoshida and H. Asanuma, *Angew. Chem. Int. Ed.*, 2010, **49**, 5502-5506.
